# Potent mechanism-based sirtuin-2-selective inhibition by an *in situ*-generated occupant of the substrate-binding site, “selectivity pocket” and NAD^+^-binding site†
†Electronic supplementary information (ESI) available: All experimental details, crystallographic data collection and refinement, details of chemical synthesis, additional figures and tables. See DOI: 10.1039/c7sc02738a



**DOI:** 10.1039/c7sc02738a

**Published:** 2017-07-21

**Authors:** Paolo Mellini, Yukihiro Itoh, Hiroki Tsumoto, Ying Li, Miki Suzuki, Natsuko Tokuda, Taeko Kakizawa, Yuri Miura, Jun Takeuchi, Maija Lahtela-Kakkonen, Takayoshi Suzuki

**Affiliations:** a Graduate School of Medical Science , Kyoto Prefectural University of Medicine , 1-5 Shimogamohangi-cho, Sakyo-ku , Kyoto 606-0823 , Japan . Email: suzukit@koto.kpu-m.ac.jp; b Research Team for Mechanism of Aging , Tokyo Metropolitan Institute of Gerontology , 35-2 Sakae-cho, Itabashi-ku , Tokyo , 173-0015 , Japan; c Minase Research Institute , Ono Pharmaceutical Co., Ltd. , 3-1-1 Sakurai Shimamoto-Cho, Mishima-Gun , Osaka 618-8585 , Japan; d Department of Chemistry and Biochemistry , School of Advanced Science and Engineering , Waseda University , Shinjuku , Tokyo 169-8555 , Japan; e School of Pharmacy , University of Eastern Finland , P.O. Box 1627 , 70211 Kuopio , Finland; f CREST , Japan Science and Technology Agency (JST) , 4-1-8 Honcho , Kawaguchi , Saitama 332-0012 , Japan

## Abstract

Sirtuin 2 (SIRT2), a member of the NAD^+^-dependent histone deacetylase family, has recently received increasing attention due to its potential involvement in neurodegenerative diseases and the progression of cancer. Potent and selective SIRT2 inhibitors thus represent desirable biological probes. Based on the X-ray crystal structure of SIRT2 in complex with a previously reported weak inhibitor (**6**), we identified in this study the potent mechanism-based inactivator KPM-2 (**36**), which is selective toward SIRT2. Compound **36** engages in a nucleophilic attack toward NAD^+^ at the active site of SIRT2, which affords a stable **36**-ADP-ribose conjugate that simultaneously occupies the substrate-binding site, the “selectivity pocket” and the NAD^+^-binding site. Moreover, **36** exhibits antiproliferative activity in cancer cells and remarkable neurite outgrowth activity. This strategy for the selective inhibition of SIRT2 should allow further probing of the biology of SIRT2, and promote the development of new disease treatment strategies.

## Abstract 2

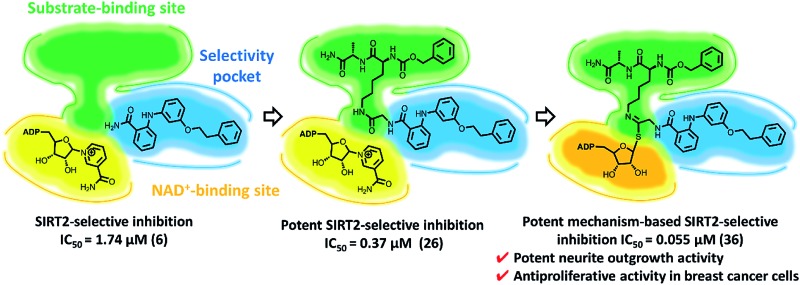
SIRT2 is potently and selectively inhibited by *in situ*-generated KPM-2 (**36**)-ADP ribose conjugate.

## Introduction

Human sirtuins (SIRT1–7) are currently the only known class of histone deacetylases that require NAD^+^ to catalyze the selective deacetylation of lysine in histone and non-histone proteins.^1^ Recently, a growing body of evidence has emerged, which suggests that sirtuins can also efficiently catalyze the removal of long chain fatty acids (SIRT1–3, 6 and 7) and 4-oxononanonyl to ε-amino lysine residues (SIRT2).^2–5^ SIRT5 exhibits desuccinylase enzymatic activity.^6^ The catalytic region of sirtuins consists of a Rossmann-fold domain, a zinc-binding domain and the loops connecting them.^7^ The interface between the loops and the two domains form both NAD^+^ (subdivided by the pockets A–C) and Ac-Lys substrate-binding sites. This region is highly conserved in SIRT1–7 and mutations can drastically reduce the enzymatic activity. Even though the sirtuin subcellular localization is strongly connected to the cell type, stress conditions and interactions with other proteins, SIRT1, 6 and 7 are mainly localized in the nuclei, SIRT3–5 are mitochondrial sirtuins, and SIRT2 is classified as a cytoplasmic isoform.^8^ The ability of sirtuins to deacetylate histones, transcription factors and nuclear receptors reflects their involvement in many physiopathological processes. The activity of sirtuins has been related to metabolic disorders,^9^ cancer progression^10^ (in which they play a Janus-faced role) and neurodegenerative diseases.^11^

Of the seven isoforms, SIRT1 is considered the genome “guardian angel” in normal cells, *i.e.*, it can regulate genomic stability through DNA repair. In cancer cells, its overexpression leads to the negative regulation of p53,^12,13^ FOXO3a,^14^ HIF1-α^15^ and a blockage of Bax translocation to the mitochondria.^16^ Thus, SIRT1 induces cancer cell survival, accumulation of DNA mutations and drug resistance. While it is undisputed that the activity of SIRT1 is correlated to the growth of several kinds of cancer cells, the exact function of SIRT2 within cancer cells was the subject of strong controversy until recently. SIRT2 has been reported to act as a tumor suppressor and as an oncogene.^17^ SIRT2 levels are reduced in various types of cancer such as glioma,^18^ liver cancer,^19^ esophageal adenocarcinoma^20^ and breast cancer,^21^ while they are increased in neuroblastoma,^21^ acute myeloid leukemia^22^ and prostate cancer.^23^ However, recent reports strongly suggest that SIRT2 plays a key role in the invasion and metastasis formation of malignancy by increasing the cell motility of cancer cells.^23^ On the other hand, the function of SIRT2 in neurons remains the subject of controversy. While it has been reported that SIRT2 inhibition impairs neurogenesis and results in depression-like behaviors,^24^ a SIRT2 inhibitor has been reported to induce antidepressant-like action.^25,26^ Thus, potent and selective SIRT2 inhibitors are desirable as biological probes.

**Fig. 1:**
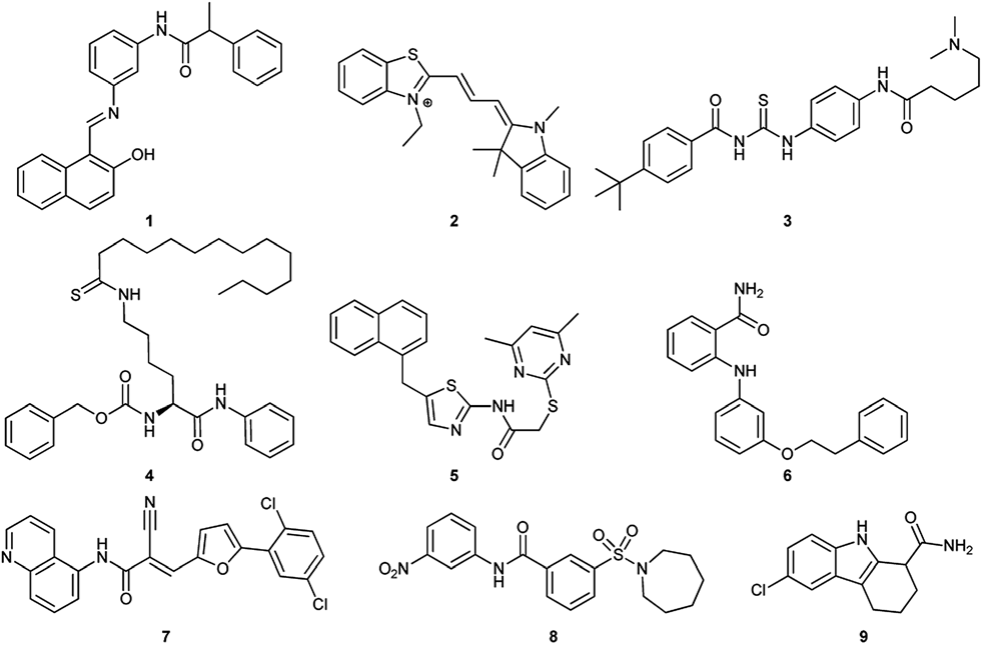
Examples of SIRT1 and SIRT2 inhibitors (**1–9**).

So far, several SIRT1 and SIRT2 inhibitors have been developed (Fig. 1), including *e.g*. salermide (**1**),^27^ AC93253 (**2**),^28^ tenovin-6 (**3**),^29,30^ TM (**4**),^31^ SirReal2 (**5**),^32,33^ 3′-phenethyloxy-2-anilinobenzamide (**6**),^34^ AGK2 (**7**),^35^ AK-1 (**8**)^36^ and EX-527 (**9**).^37^ These compounds have shown antiproliferative activity when tested in cancer cells and some of these have shown beneficial effects in neurological disorder models, but in many cases, the lack of potency and isoform specificity suggests their effect is not solely related to SIRT1/2 inhibition. Therefore, the development of more potent and specific compounds is currently of high interest.

In the field of SIRT2 inhibitor design, a milestone on the elucidation of the mechanism behind the isotype selectivity of SIRT2 inhibitors has recently been reported by Rumpf *et al.*, who discovered that the novel drug-like small molecule SirReal2 (**5**)^32,33^ is able to induce a rearrangement of the SIRT2 active site by creating a binding pocket, called the “selectivity pocket”, which is responsible for isoform specificity, suggesting a potential common mode of action for selective SIRT2 inhibitors. In fact, the “selectivity pocket” is the pocket that accommodates the long chain fatty acyl groups.^3,38,39^

Previously, we proposed a novel class of 2-anilinobenzamides^34^ as selective SIRT2 inhibitors, in which a phenylethyl ether moiety acted as the optimal fragment to improve both potency and isoform selectivity. Herein, with the aim to elucidate the binding mode of this prototype and to further improve the inhibitory activity, we initially solved the single-crystal X-ray diffraction structure of SIRT2 in complex with 3′-phenethyloxy-2-anilinobenzamide (**6**),^34^ which revealed that **6** is located in the selectivity pocket. Then, inspired by this result, we applied structural modifications to the amide moiety in order to target the acetyl–lysine-substrate-binding site and NAD^+^-binding pockets, which finally led to the identification of KPM-2 (**36**) as a novel class of mechanism-based SIRT2 inactivators. Moreover, **36** shows highly potent and selective SIRT2 inhibition with antiproliferative activity in breast cancer cells and potent neurite outgrowth activity in neuro-2a (N2a) cells.

## Results and discussion

### Binding mode analysis of 2-anilinobenzamide **6** in complex with SIRT2

**Fig. 2:**
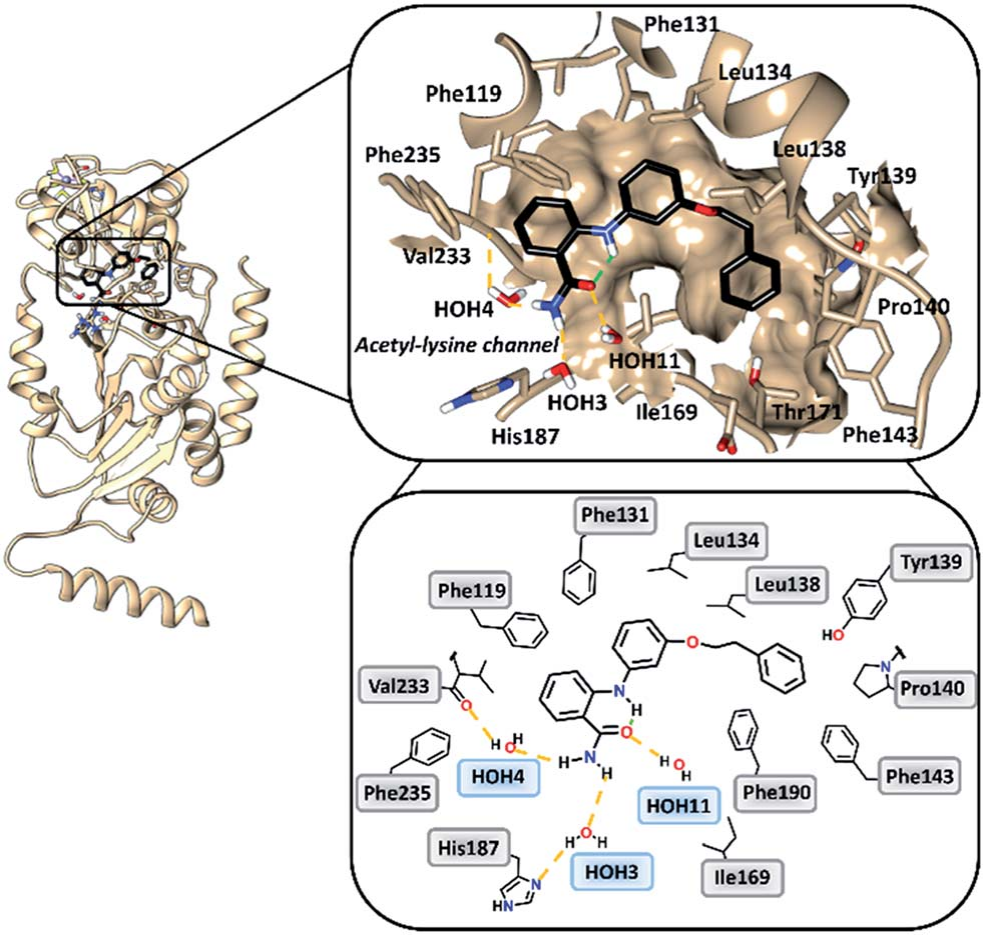
Overall structure of SIRT2/**6** (light brown ribbon; left). The windows on the right show magnifications of the inhibitor-binding mode, wherein **6** (black) occupies the SIRT2 selectivity pocket delineated by the molecular surface. Intramolecular (green) and intermolecular (yellow) hydrogen bonds are illustrated by dashed lines.

To gain insight into the binding mode of 2-anilinobenzamides, we determined the single-crystal X-ray diffraction structure of SIRT2 in complex with **6** (Table S1†). In general, the overall fold of complex SIRT2/**6** is similar to the previously reported SIRT2 structure in complex with the SIRT2-selective SirReal inhibitors (PDB code: ; 5DY5, ; 5DY4, ; 4RMG and ; 4RMH), whereby the main divergences occur at the cofactor binding loop (Gly92-Leu112), which is not well defined, and at the downward-shifted connection loop at β8–α10 (ref. 40) (Thr262-Phe269). The inhibitor binds to a highly lipophilic pocket localized at the interface between the Rossmann-fold domain and a small domain adjacent to the C-pocket of the NAD^+^-binding site. The superimposition of the SIRT2/**6** crystal structure with that of the SIRT2 apo structure (PDB: ; 3ZGO, Fig. S1†) demonstrates that **6** induces a shift of the helix α5 (ref. 40) and the connection loop to α6 (Glu129-Thr146), which is responsible for the accommodation of the inhibitor. The binding mode analysis of **6** (Fig. 2) revealed that its phenoxyethylphenyl moiety is surrounded by Phe131, Leu134, Leu138, Tyr139, Pro140, Phe143 and Ile169, which leads to the establishment of π–π and H–π interactions. The 2-aminobenzamide moiety oriented towards the acetyl–lysine tunnel exhibits hydrogen bonds with water molecules HOH3, HOH4 and HOH11. Of these three water molecules, HOH3 and HOH4 are stabilized by a hydrogen bond network with the side chain of the catalytically active His187 and the main chain of Val233, *i.e.*, the key amino acid residues that stabilize the binding of the acetyl–lysine substrate during the deacetylation reaction.^40^ Furthermore, **6** shows an intramolecular hydrogen bond between the amide (–C=O) and aromatic amine (–NH) moieties. This partially locked conformation renders the speculation feasible that there might be an additional structural requirement to achieve SIRT2 inhibition, as previously observed for SIRT1.^41^

**Fig. 3:**
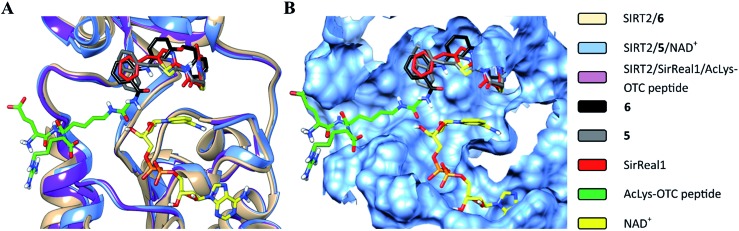
Superimposition of SIRT2/**6** with the complex structures of **5**/NAD^+^ (PDB: ; 4RMG) and the SirReal1/AcLys OTC peptide substrate (PDB: ; 4RMI). (A) Colored ribbons; (B) molecular surface; the binding sites of the acetylated substrate, NAD^+^, **6**, **5** and SirReal1 are shown in cyan. The carbon atoms of **6**, SirReal1, **5**, AcLys-OTC peptide and NAD^+^ are colored in black, grey, red, green and yellow, respectively.

The structural overlay of the SIRT2/**6**, SIRT2/**5**/NAD^+^ (PDB: ; 4RMG) and SIRT2/SirReal1/AcLys-OTC peptide (PDB: ; 4RMI) crystal structures demonstrate that inhibitors **6**, SirReal1 and **5** occupy the same binding site (Fig. 3 and S2†).

### Design of novel SIRT2-selective inhibitors and their *in vitro* biological evaluation

**Fig. 4:**
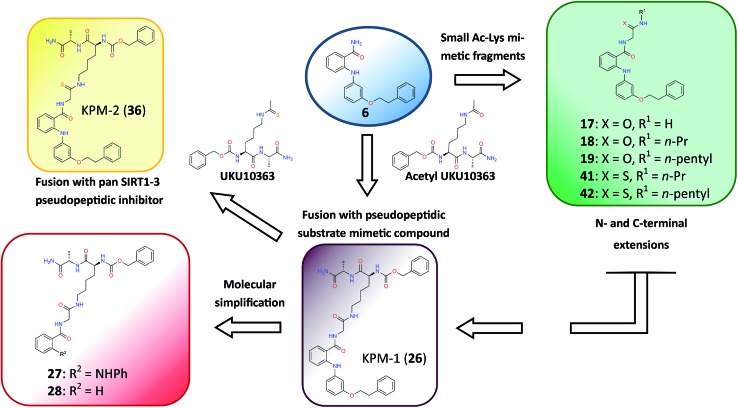
Molecular design applied to **6** in order to mimic acetyl–lysine substrate interactions.

**Fig. 5:**
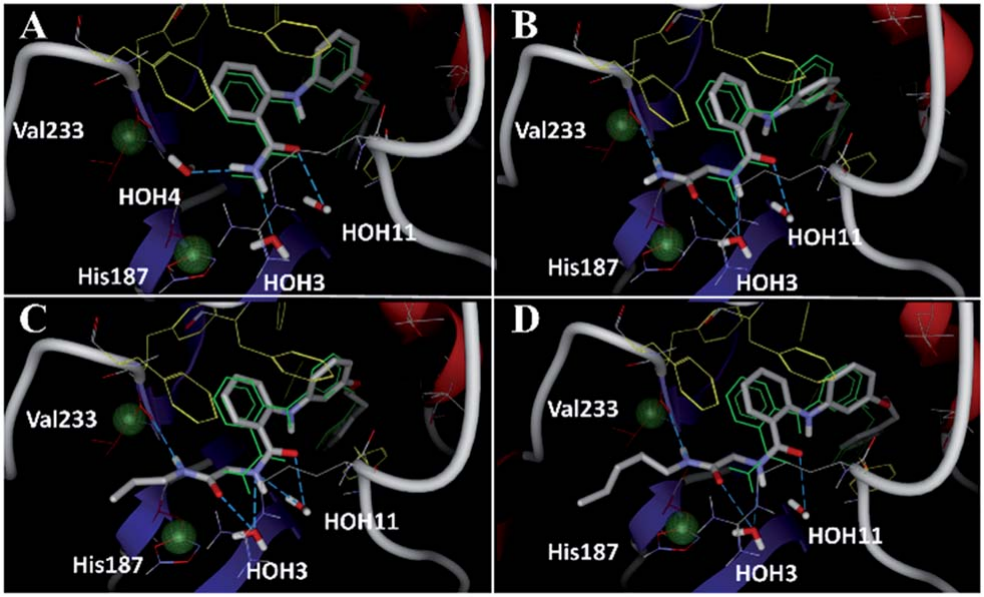
Docking poses for **6** ((A), redocked ligand RMSD 0.70 Å), **17** (B), **18** (C), and **19** (D) in the SIRT2/**6** crystal structure. Crystallized ligand **6** and docking poses are colored in green and gray, respectively. Val233 and His187 are colored in red and delimited by green spheres. The Phe residues surrounding selectivity pocket are colored in yellow; hydrogen bonds are represented by dashed lines.

**Fig. 6:**
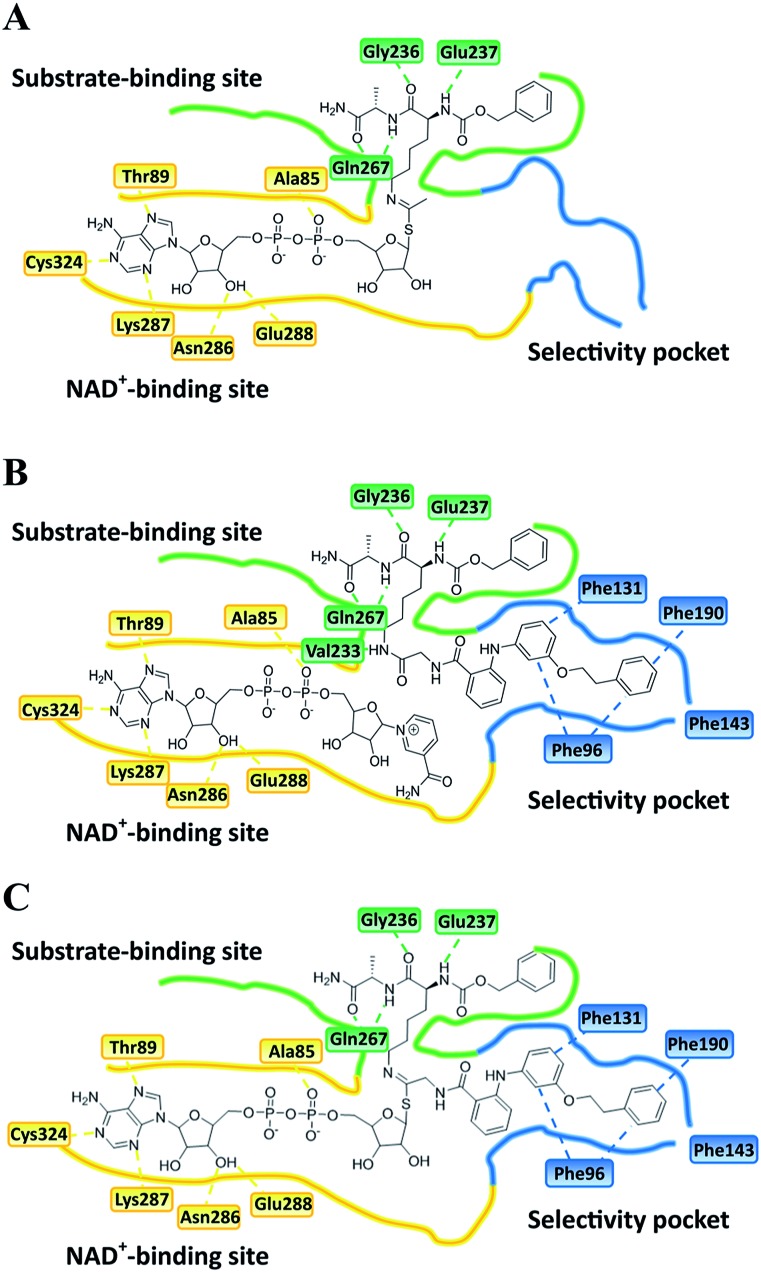
Plausible binding mode for (A) UKU10363, (B) **26** and (C) **36**. The substrate-binding site, NAD^+^-binding site, and selectivity pocket are outlined by green, yellow and blue lines, respectively.

Closer evaluation of the superimposed SIRT2 structures revealed that the amide group of **6** might occupy the same space as the methyl group of the *N* ^ε^-acetyl–lysine substrate (Fig. 3). This result prompted us to take a new perspective toward targeting the substrate-binding site through the functionalization of the benzamide moiety in **6** to mimic the acetyl–lysine interactions, which afforded compounds **17–19**, **26–28**, **36**, **41** and **42** (Fig. 4). All compounds prepared in this study (Schemes S1–4†) were screened *in vitro* against SIRT1–3 and SIRT5 (concentration: 10 μM; Table 1), while **6**, UKU10363 (Fig. 4) and **5** were used as reference compounds. In a first attempt to mimic the lysine acetyl amide, we explored small fragments using a glycinamide linker (**17–19**). The screening of SIRT1–3 and 5 (Table 1) revealed that both isotype selectivity and good SIRT2 inhibitory activity were retained upon amide functionalization of **6**, although the inhibitory potency was not improved. In order to rationalize the results, the putative binding mode of **17–19** was studied *via* docking simulations (Fig. 5). The glycinamide handle exhibits hydrogen bonding with HOH3 and HOH11 (Fig. 5B–D), located in proximity of the amide groups of **17–19**, and the water molecules can be displaced by NAD^+^ once it binds to the active site. The glycinamide substituents in **17–19** displace HOH4, thus mimicking the behavior of acetyl–lysine. This may lead to the direct interaction of **17–19** with Val233 (Fig. 5B–D) and a subsequent loss of hydrogen bonding with HOH4, resulting in an “abated” inhibitory activity. In order to test whether SIRT2 inhibition could be improved by introducing N-, C-terminal pseudopeptidic extensions on **19**, we designed KPM-1 (**26**). A methylene bridge was used to link **6** with the acetyl derivative of UKU10363,^42^ which is a previously identified pan-SIRT1–3 inhibitor (Fig. 4). UKU10363 has been proposed to inhibit SIRT1–3 through the attack of its thioacetyl group to NAD^+^ to afford a UKU10363-ADP-ribose conjugate (Fig. S3A, S3B† and 6A). The occupation of the SIRT2 selectivity pocket by a single molecule through the 2-anilinobenzamide core and the substrate-binding site through the pseudopeptidic backbone should lead to potent inhibition (Fig. 6B). The screening output revealed a strong and selective SIRT2 inhibitory effect (IC_50_ = 0.37 μM) by **26** (Table 1), which was fourfold more active than lead compound **6** and equipotent to UKU10363 and **5**. This result supports the hypothesis that targeting both the substrate-binding site and the selectivity pocket by linking two distinct inhibitor scaffolds represents a useful strategy to develop novel SIRT2 inhibitors. In order to obtain additional information on the structure–activity relationship around **26**, the 2-anilinobenzamide core was simplified in **27** and **28** (Fig. 4). As expected, the removal of the phenoxyethyl fragment in **27** reduced the SIRT2 inhibitory activity by ∼20% (Table 1), while further simplification of the core in **28** led to no SIRT2 inhibition. These results are consistent with those of our previous 2-anilinobenzamide SAR study.^34^ Then, we turned our efforts toward finding a suitable strategy to boost the inhibitory potency of **26**. Thioacetyl–lysine peptide/pseudopeptide inhibitors interact stronger with the substrate-binding site than their acetylated counterparts through the formation of a stalled intermediate with NAD^+^, inducing a retardation of the enzymatic turnover rate (Fig. 6A and S3B†).^43,44^ Following this lead, we designed KPM-2 (**36**) to test whether replacement of the acyl group in **26** with a thioacyl group could lead to an improvement of the SIRT2 inhibitory activity by generating a novel trapped intermediate (Fig. S3C†) and, consequently, the full occupation of the substrate/NAD^+^-binding site and the selectivity pocket (Fig. 6C). At 1 μM concentration, **36** potently inhibited SIRT2, even though it did not show strong inhibition towards SIRT1 and SIRT3. As indicated in Table 1, **36** is a moderate SIRT1 and SIRT3 inhibitor, while it potently inhibits SIRT2 (SIRT1 [IC_50_]/SIRT2 [IC_50_] = 28.4; SIRT3 [IC_50_]/SIRT2 [IC_50_] = 172.5) with an IC_50_ of 55 nM, which is by a factor of ∼32 lower than that of **6**, and by a factor of ∼11 lower than UKU10363. Notably, the SIRT2-inhibitory activity of **36** was much greater than representative previously reported SIRT2 inhibitors **3** and **5–9**. The moderate SIRT1 and SIRT3 inhibition by **36** is not surprising as SIRT1 and SIRT3 are also known to be a demyristoylase and have a pocket similar to the SIRT2 selectivity pocket that can accommodate the long chain fatty acyl group.^3^

**Table 1:** Screening of derivatives of **6**, UKU10363, **3**, **5**, **7–9**, **17–19**, **26–28**, **36**, **41** and **42**

Cpd.	% inhibition at 10 μM^*a*^				IC_50_ ± SD^*c*^ [μM] or % inhibition at 50 μM			
	SIRT1	SIRT2	SIRT3	SIRT5	SIRT1	SIRT2	SIRT3	SIRT5
**6**	3 ± 3.6	90 ± 1.5	1 ± 2.6	10 ± 2.6	14% at 50 μM, >300^*d*^	1.74 ± 0.26	12% at 50 μM, >300^*d*^	12% at 50 μM
		32 ± 4.1^*b*^				1.0 ± 0.12^*d*^		
UKU 10363	97 ± 0.04	99 ± 3.2	65 ± 0.23	3 ± 3.9	0.18 ± 0.013	0.59 ± 0.13	4.39 ± 0.28	6% at 50 μM
	83 ± 0.4^*b*^	56 ± 1.9^*b*^	19 ± 0.78^*b*^					
**17**	10 ± 1.9	85 ± 0.47	10 ± 0.95	0 ± 1.1	18% at 50 μM	3.60 ± 0.03	13% at 50 μM	4% at 50 μM
		19 ± 0.63^*b*^						
**18**	2 ± 1	81 ± 0.43	0 ± 1.1	0 ± 0.2	12% at 50 μM	4.04 ± 0.65	3% at 50 μM	3% at 50 μM
**19**	4.5 ± 3.4	69 ± 2.2	3 ± 3.2	6 ± 3.2	4% at 50 μM	6.71 ± 0.42	2% at 50 μM	6% at 50 μM
**26**	16 ± 0.1	99 ± 0.15	14 ± 4.9	0 ± 3.5	25% at 50 μM	0.37 ± 0.03	56% at 50 μM	6% at 50 μM
		74 ± 2^*b*^						
**27**	24 ± 2.1	80 ± 0.12	9 ± 2.2	0 ± 2.5	68% at 50 μM	1.84 ± 0.21	16% at 50 μM	3% at 50 μM
**28**	3.5 ± 1	8 ± 3.3	1 ± 1.8	0 ± 0.6	1% at 50 μM	18% at 50 μM	0% at 50 μM	0% at 50 μM
**36**	29.5 ± 2.1^*b*^	100 ± 3.4^*b*^	2 ± 1.8^*b*^	4 ± 2.6	1.56 ± 0.06	0.055 ± 0.0035	9.49 ± 1.7	10% at 50 μM
**5**	3 ± 0.65	98 ± 3.1	5 ± 0.6	3 ± 1.5	6% at 50 μM, >100^*e*^	0.30 ± 0.06, 0.4^*e*^	1% at 50 μM, >100^*e*^	1% at 50 μM
		80 ± 1.5^*b*^						
**3**	0.9 ± 0.17	45 ± 2.5	0 ± 1.0	0 ± 1.4	64% at 50 μM	9.66 ± 2.3	9% at 50 μM	2% at 50 μM
**7**	0.9 ± 0.62	45 ± 2.9	0 ± 1.0	0 ± 1.1	14% at 50 μM	11.5 ± 3.8	14% at 50 μM	3% at 50 μM
**8**	6.2 ± 0.85	31 ± 0.42	0 ± 0.89	0 ± 0.05	25% at 50 μM	30.1 ± 1.3	0% at 50 μM	0% at 50 μM
**9**	97 ± 0.13	79 ± 1.3	20 ± 1.2	0 ± 1.1	0.24 ± 0.01	2.53 ± 0.04	54% at 50 μM	0% at 50 μM
**41**	0 ± 0.84	54 ± 1.5	0 ± 0.50	1 ± 2.2	10% at 50 μM	8.47 ± 0.30	0% at 50 μM	3% at 50 μM
**42**	0 ± 1.1	40 ± 3.4	0 ± 1.1	0 ± 0.23	11% at 50 μM	19.3 ± 3.1	2% at 50 μM	3% at 50 μM

^*a*^Fluor de Lys assay; values represent mean values ± standard deviation of at least two experiments.

^*b*^% inhibition at 1 μM.

^*c*^Fluor de Lys assay; values are calculated from three independent determinations, which afford a total of 21 data points (Fig. S4).

^*d*^Data from ref. 34.

^*e*^Data from ref. 32.

In addition, we examined the SIRT-inhibitory activity of compounds **41** and **42**, thioamide analogues of **18** and **19**, respectively. As shown in Table 1, even though SIRT2 inhibition was observed with **41**, no improvement was achieved as compared with **18**. Furthermore, the elongation of aliphatic chain (**19**) resulted in a loss of potency. These results suggest that compounds **41** and **42** do not react with NAD^+^ because the short alkyl chain of **41** and **42** is too flexible to fix the thioamide group in proximity to the nicotinamide moiety of NAD^+^.

### Mechanistic analysis of SIRT2 inhibition by **36**

**Fig. 7:**
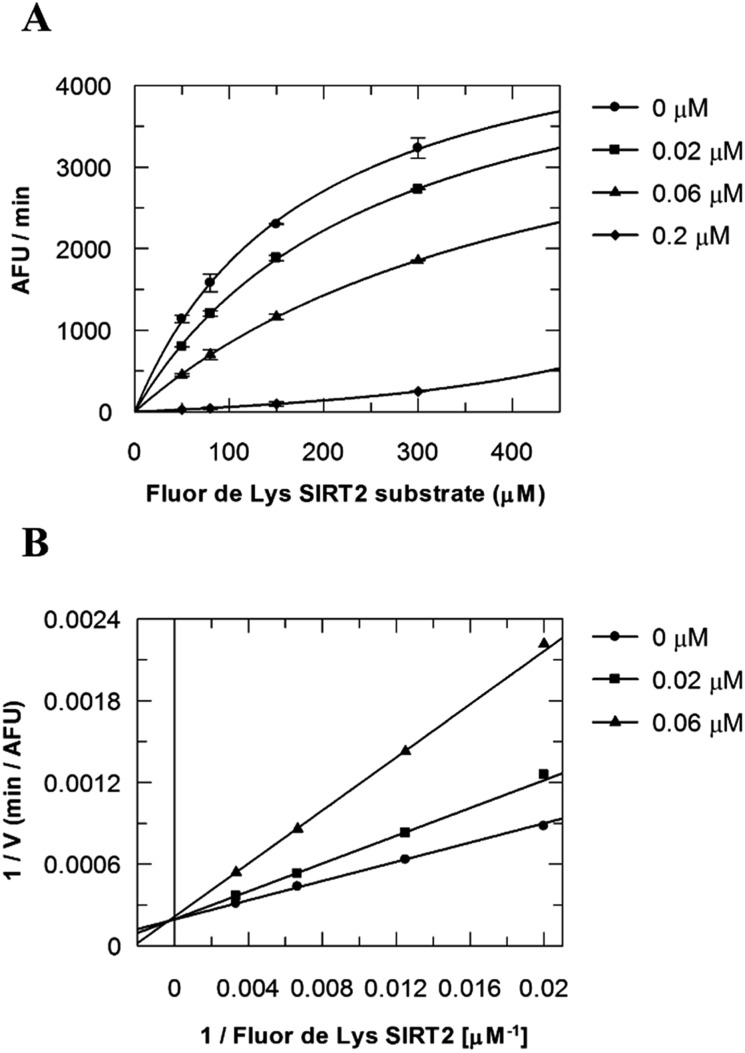
Competition analysis for **36** with the acetylated lysine substrate. (A) Michaelis–Menten plot showing the Fluor de Lys SIRT2 substrate (μM) competition analysis for of **36** at 0, 0.02, 0.06 and 0.2 μM. (B) Lineweaver–Burk plot: 1/*V* as a function of the reciprocal acetylated lysine substrate in the presence of 0, 0.02 and 0.06 μM of **36**. Values were calculated from two independent determinations.

**Fig. 8:**
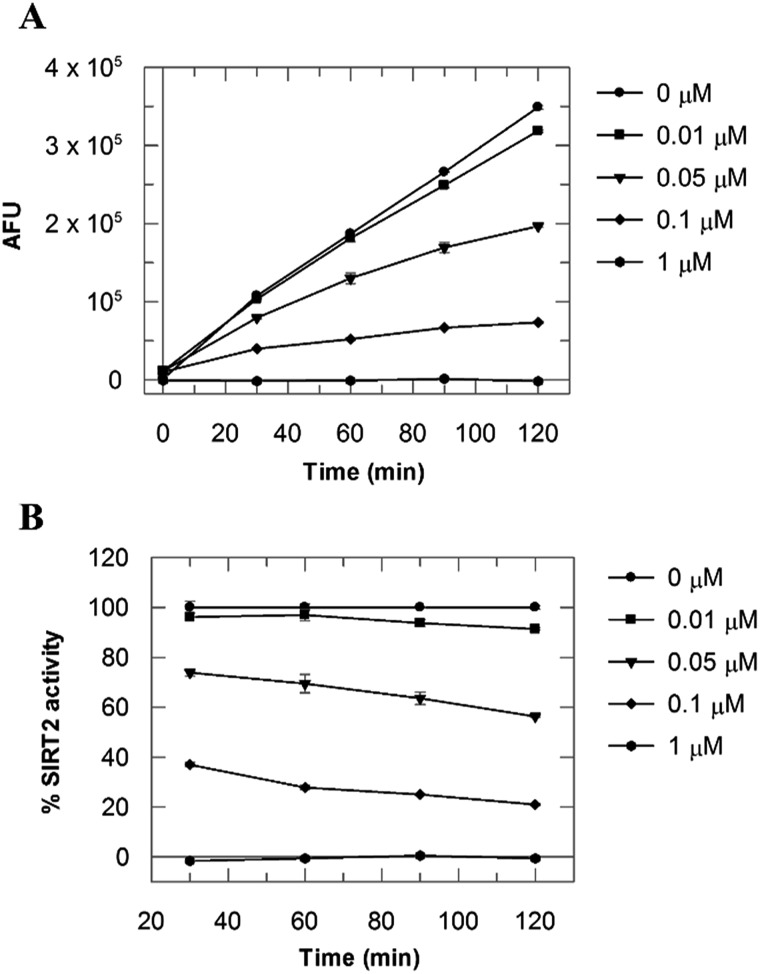
(A) SIRT2 time reaction course at 0, 0.01, 0.05, 0.1 and 1 μM of **36**. (B) SIRT2 activity expressed as % of the control value. Values were calculated from two independent determinations.

Due to the peculiar structure of the novel SIRT2 inhibitor, further investigations seemed pertinent. To elucidate the **36**-induced SIRT2 inhibition mechanism, a substrate competition analysis was carried out (Fig. 7A). The double reciprocal plot of initial velocity as a function of substrate concentration 1/*V* against 1/[*S*] revealed a series of regression lines intersecting the 1/*V* axis, suggesting competitive inhibition with the acetylated lysine substrate (Fig. 7B). Furthermore, the apparent Michaelis constant *K*_m_ of the substrate increased with increasing concentration of **36** under NAD^+^ saturation conditions. Subsequently, we monitored the SIRT2 reaction course in the presence and absence of **36** (Fig. 8A). The inhibitory effect was time-dependent and exhibited a pronounced effect after 120 min of simultaneous incubation of SIRT2, the acetylated substrate, NAD^+^ and **36** (Fig. 8B). These results support a potential SIRT2 mechanism-based inhibition. To confirm that the time-dependent SIRT2 inhibition by **36** was not due to an assay artifact or chemical instability, a SIRT deacetylated standard assay and an HPLC stability test were carried out (Fig. S5 and S6†). Under the applied conditions, **36** was stable and did not inhibit the developer reaction of the SIRT2 assay used in this study.

**Fig. 9:**
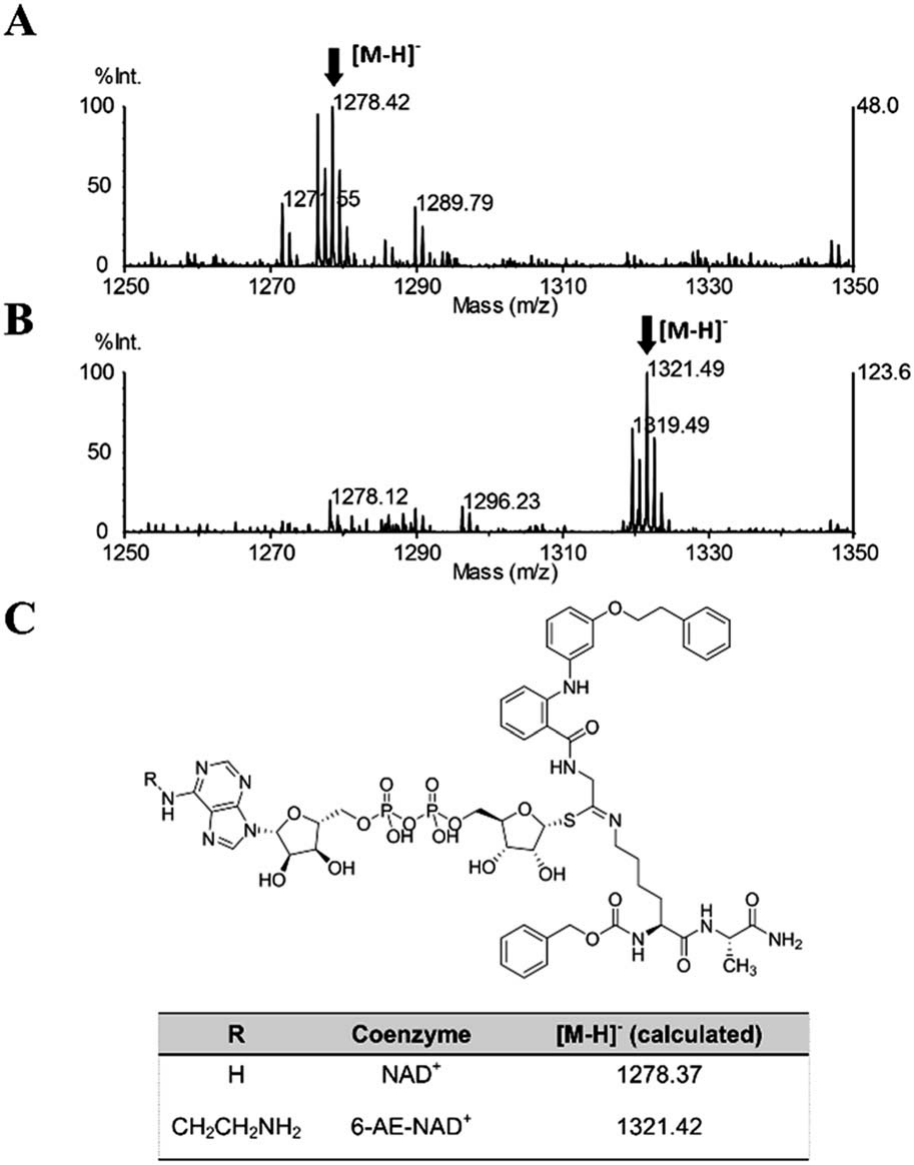
MALDI-TOF mass spectrometric detection of the ADP-ribose conjugate formed between **36** and (A) NAD^+^ or (B) 6-AE-NAD^+^. (C) Chemical structure of the conjugate and the calculated *m*/*z* value for the [M – H]^–^ ion.

Once proven that **36** is a substrate-competitive and time-dependent inhibitor, a MALDI-TOF (matrix-assisted laser desorption/ionization time-of-flight) mass spectrometric analysis was conducted, in order to determine whether the **36**-ADP-ribose conjugate could be detected after simultaneous incubation of **36** with SIRT2 and NAD^+^ (Fig. S3C† and 6C). As shown in Fig. 9A, a peak with *m*/*z* 1278.42 corresponding to [M – H]^–^ of **36**-ADP-ribose conjugate was observed only in the presence of SIRT2 (Fig. S7†). In order to provide additional insight, a similar experiment was carried out using 6-aminoethyl (AE)-NAD^+^. Upon generation of the trapped intermediate, a mass shift of +43 Da from that of the **36**-ADP-ribose conjugate should be observed. As shown in Fig. 9B, a mass peak of *m*/*z* 1321.49 confirmed the ability of **36** to specifically react with 6-(AE)-NAD^+^, which is catalyzed by SIRT2. In their entirety, these results suggest that **36** is a substrate-competitive inhibitor. Moreover, they indicate that **36** specifically reacts in a time-dependent manner with NAD^+^ exclusively in the presence of SIRT2, which affords a Michaelis adduct intermediate, supporting the hypothesis that **36** is a SIRT2-mechanism-based inhibitor.

### The effect of **26** and **36** in breast cancer cells (MDA-MB-231 and MCF-7)

**Fig. 10:**
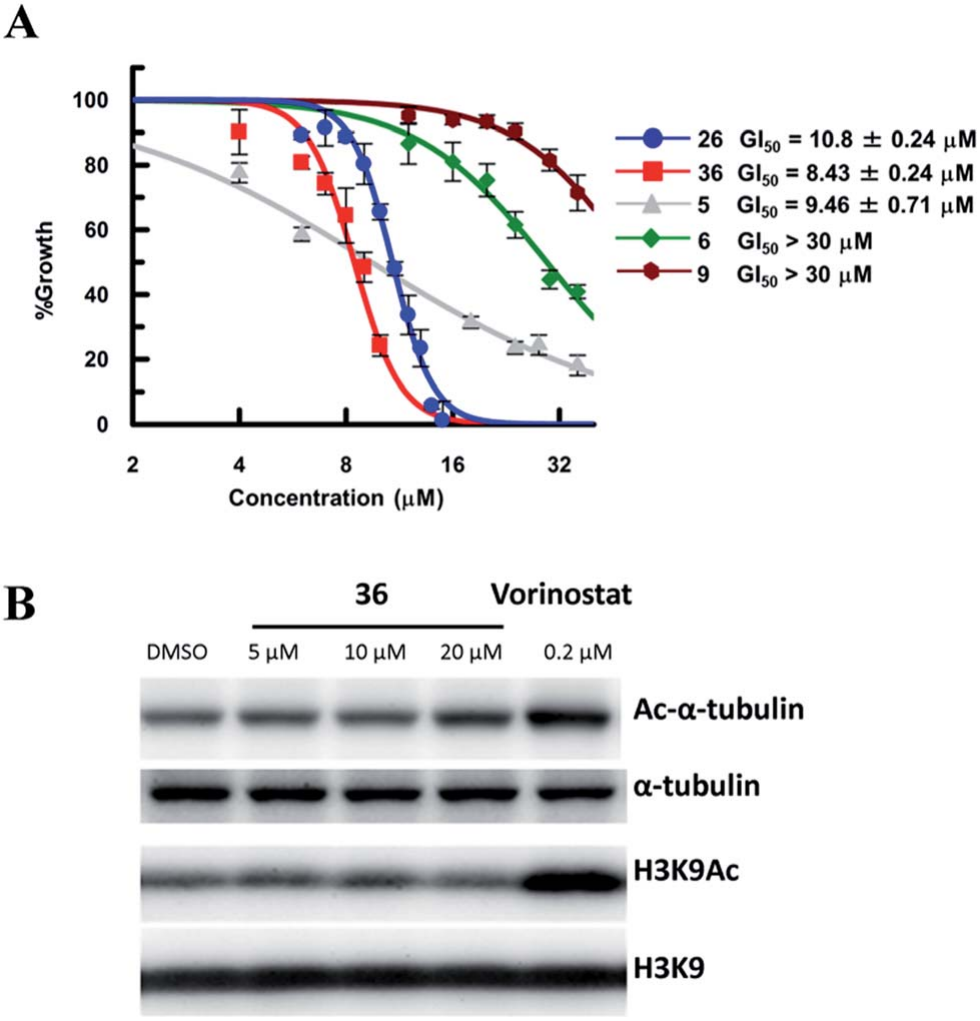
(A) Antiproliferative activity of **5**, **6**, **9**, **26** and **36** in MDA-MB-231 cells after 72 h of treatment. Values were calculated from three independent determinations. (B) Western blot detection of α-tubulin or H3K9 acetylation levels in MDA-MB-231 cells after 6 h treatment with **36** or vorinostat, which is a pan-HDAC inhibitor. The latter was used as a positive control that induces acetylation of both α-tubulin^45^ and H3K9.^46^

So far, valuable antiproliferative activity in cancer cells has been established for several SIRT1/2 inhibitors. However, on account of their questionable potency of action and selectivity profile, a possible off-target activity could not be ruled out. In this study, the ability of the highly potent and selective SIRT2 inactivators **26** and **36** toward the reduction of MDA-MB-231 and MCF-7 cancer cell proliferation and the selective intracellular inhibition of SIRT2 was examined. As reference compounds, **5**, **6** and EX-527 (**9**)^37^ were included in the assay as a potent SIRT2-selective inhibitor, a weak SIRT2-selective inhibitor and a potent SIRT1-selective inhibitor, respectively. After 72 h of incubation, GI_50_ values were determined. Neither the potent and selective SIRT1 inhibitor **9**, nor the weak SIRT2-selective inhibitor **6** showed significant antiproliferative activity against MDA-MB-231 cells (GI_50_ > 30 μM; Fig. 10A). However, under the applied conditions, **5** and **26** efficiently reduced the proliferation of MDA-MB-231 cells (GI_50_ = 9.46 μM and 10.8 μM, respectively). **36**, *i.e.*, the most potent SIRT2 inhibitor, showed the highest activity (GI_50_ = 8.3 μM). We also examined the effect of **6**, **9**, **26** and **36** in MCF-7 cells (Fig. S8†). The antiproliferative activity of **6**, **9**, **26** and **36** in MCF-7 cells was similar to that in MDA-MB-231 cells, with a strong correlation between the antiproliferative activity and the SIRT2-inhibitory activity of the inhibitors. These results suggest that SIRT2-selective inhibition is responsible for the observed cancer cell growth inhibition. Subsequently, **36** was used for further examination of its cellular “isotype selectivity” using western blotting (Fig. 10B). Treatment of MDA-MB-231 breast cancer cells with 20 μM **36** induced the accumulation of acetylated α-tubulin, which is a known SIRT2 substrate,^47^ in the cells. On the other hand, **36** did not upregulate the levels of acetylated lysine **9** on histone H3 (H3K9Ac), which is a SIRT1 substrate,^48–50^ in MDA-MB-231 cells. In their entirety, these results suggest that **36** selectively inhibits SIRT2 over SIRT1 in the cellular context.

### Effect of **36** on the neurite outgrowth of neuro-2a cells

**Fig. 11:**
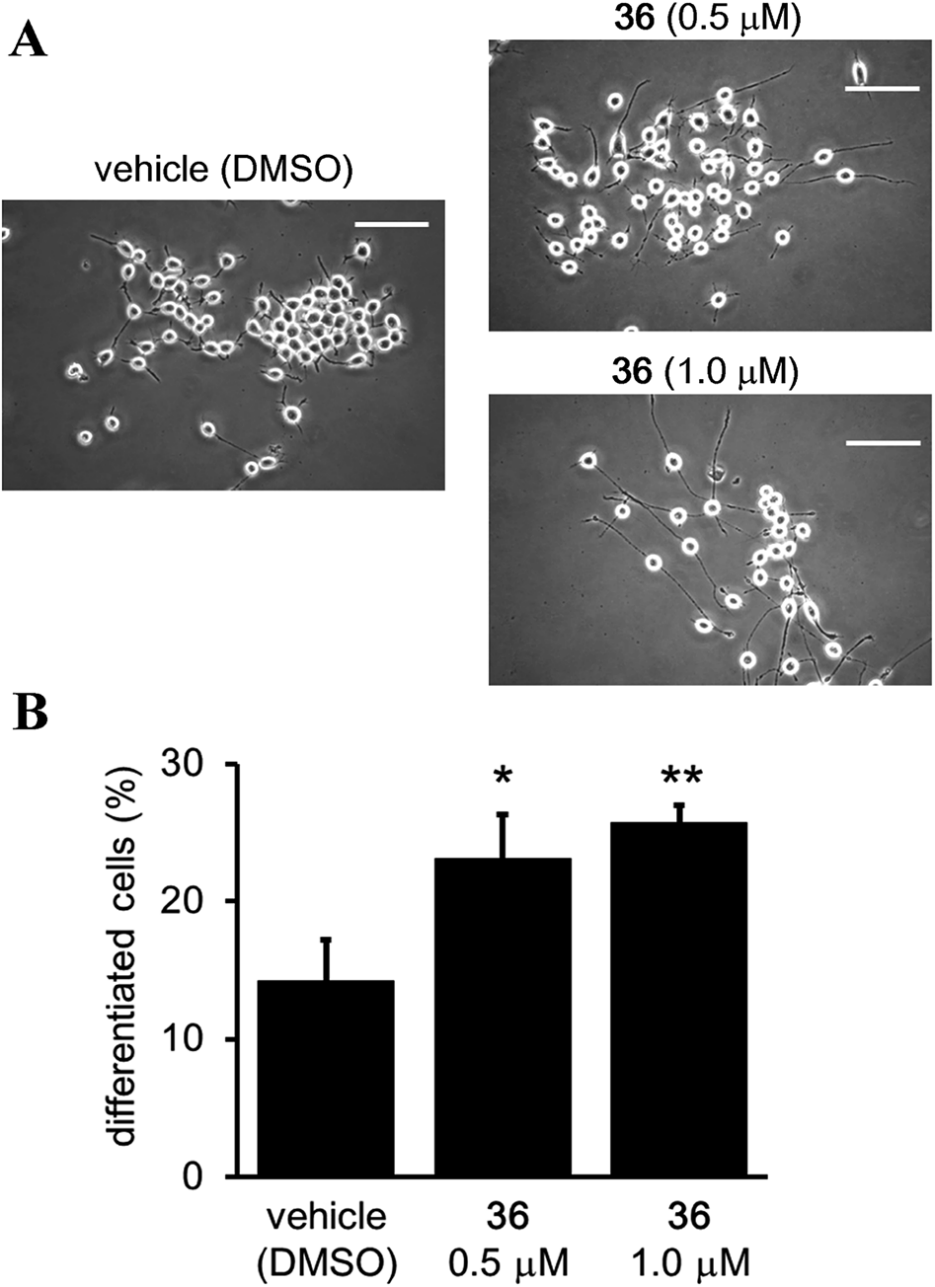
Effect of **36** on neurite outgrowth. (A) Representative images showing N2a cells treated with **36** (0.5 and 1 μM) or vehicle (0.1% DMSO) for 72 h. Scale bar: 100 μm. (B) Effect of **36** on N2a differentiation, represented as the % of differentiated cells relative to the total counted cells (at least 100 cells for each condition). Bars represent the mean values ± SD from three independent experiments; *p < 0.05, **p < 0.01, compared to the vehicle group.

Although it has been suggested that SIRT2 inhibition by small molecules might be beneficial in the context of neurological disorders,^25,26,34,51,52^ the function of the catalytic activity of SIRT2 in neurogenesis is still unclear.^24^ Therefore, we investigated the effect of the SIRT2-selective inhibitor **36** on neurite outgrowth. Mouse neuroblastoma neuro-2a (N2a) cells, which have previously been validated for the study of neurite morphology in neuronal differentiation, were used in this study.^53,54^ Treatment of N2a cells with **36** (0.5 and 1 μM) for 72 h induced neurite outgrowth (Fig. 11A) and significantly increased the percentage of differentiated cells relative to the vehicle control group (0.5 μM and 1 μM of **36** *vs.* vehicle; 23.09 ± 3.21% and 25.73 ± 1.3% *vs.* 14.22 ± 2.9%; **36** 0.5 μM *vs.* vehicle, *p* < 0.05, **36** 1 μM *vs.* vehicle, *p* < 0.01; Fig. 11B). These results suggest that the catalytic activity of SIRT2 is associated with neurite outgrowth and that SIRT2-selective inhibitors may be useful therapeutic agents for neurological disorders.

## Conclusions

Since 2012, 2-anilinobenzamides have been considered as one of the most promising classes of drug-like SIRT2 inhibitors. In this study, the binding mode of **6** on SIRT2 was elucidated using X-ray crystallography. On the basis of these results, a new molecular design was developed around the scaffold of this compound. Herein, we demonstrated that targeting of both the SIRT2 selectivity pocket and the substrate-binding site with **26** represents an efficient strategy to achieve potent and selective inhibition. Furthermore, the molecular conjugation of two distinct sirtuin inhibitors, UKU10363 and **6**, through a simple methylene bridge led to the new potent SIRT2 inhibitor **36**. Kinetic and mass spectroscopic analyses supported the hypothesis that **36** is a mechanism-based inhibitor that affords *in situ* occupation of the substrate-binding site, the selectivity pocket, and the NAD^+^-binding site. Thus, **36** should be a valuable tool for the detailed investigation of the effect of SIRT2 inhibition in the cellular context. In MDA-MB-231 and MCF-7 breast cancer cell cultures, **36** showed antiproliferative activity with selective acetylation the SIRT2 substrate α-tubulin. Moreover, **36** showed neurite outgrowth activity in N2a cells, suggesting the possibility of SIRT2-selective inhibitors as therapeutic agents for neurological disorders.

Our strategy to simultaneously target the substrate-binding site, the selectivity pocket and the NAD^+^-binding site provides a new approach to the design of mechanism-based selective sirtuin inhibitors, which could be potentially extended to explore and probe the limits of the SIRT1–7 active sites.

## Conflict of interest

The authors declare no competing financial interest.
