# Supplementary material for: Potent mechanism-based sirtuin-2-selective inhibition by an in situ-generated occupant of the substrate-binding site, “selectivity pocket” and NAD+-binding site
Source: Chem Sci. 2017 Jul 21;8(9):6400–8. doi: 10.1039/c7sc02738a (PMC5628579; doi:10.1039/c7sc02738a)
Supplement: Supplementary file 1 [file SC-008-C7SC02738A-s001.pdf]

Electronic Supplementary Material (ESI) for Chemical Science.
This journal is © The Royal Society of Chemistry 2017

Supplementary Information

Potent Mechanism-Based Sirtuin-2-Selective Inhibition by an *In-Situ*-Generated Occupant of the Substrate-Binding Site, "Selectivity Pocket" and NAD^+-Binding Site

Paolo Mellini,[a] Yukihiro Itoh,[a] Hiroki Tsumoto,[b] Ying Li,[a] Miki Suzuki,[a] Natsuko Tokuda,[c] Taeko Kakizawa,[d] Yuri Miura,[b] Jun Takeuchi,[c] Maija Lahtela-Kakkonen[e] and Takayoshi Suzuki*[af]

[a] *Graduate School of Medical Science, Kyoto Prefectural University of Medicine, 1-5 Shimogamohangi-cho, Sakyo-ku, Kyoto 606-0823, Japan.*
[b] *Research Team for Mechanism of Aging, Tokyo Metropolitan Institute of Gerontology, 35-2 Sakae-cho, Itabashi-ku, Tokyo, 173-0015, Japan.*
[c] *Minase Research Institute, Ono Pharmaceutical Co., Ltd., 3-1-1 Sakurai Shimamoto-Cho, Mishima-Gun, Osaka 618-8585, Japan.*
[d] *Department of Chemistry and Biochemistry, School of Advanced Science and Engineering, Waseda University, Shinjuku, Tokyo 169-8555, Japan.*
[e] *School of Pharmacy, University of Eastern Finland, P.O. Box 1627, 70211 Kuopio, Finland.*
[f] *CREST, Japan Science and Technology Agency (JST), 4-1-8 Honcho, Kawaguchi, Saitama 332-0012, Japan.*
* *Correspondence: suzukit@koto.kpu-m.ac.jp*

Contents **Page**

Supplementary Figures

**Fig. S1, S2**	Structural overlay of SIRT2/**6** and the SIRT2 apo structure, as well as SIRT2/**6** and SIRT2/**5**/NAD^+.	**S3**
**Fig. S3**	Deacetylation mechanism catalyzed by sirtuins and inhibition mode of thioacetyl-based inhibitors including **36**.	**S4**
**Fig. S4**	IC_{50} curves of **6**, **17**, **26**, **36**, UKU10363, and **5**.	**S5**
**Fig. S5**	Evaluation of the interference of **26** and **36** with the Developer II reaction on the SIRTs assay.	**S6**
**Fig. S6**	HPLC stability of **36** under SIRTs assay conditions.	**S7 -S9**
**Fig. S7**	MALDI-TOF blank spectra carried out for the detection of the **36**-ADP-ribose conjugate.	**S10**
**Fig. S8**	Growth inhibition effect of **6**, **26**, **36**, and EX-527 (**9**) in MCF-7 cancer cells.	**S11**

Supplementary Table **Page**

**Table S1**	Data collection and refinement for the **6**-SIRT2 crystal structure.	**S12**

Supplementary Methods **Page**

	SIRT assays.	**S13-S14**
	Cell assays.	**S14-S16**
	Crystallization, data collection and figures preparation	**S16**
	Chemical synthesis of **10-42**.	**S16-S34**
	HPLC chromatograms for **17-19**, **26-28**, **36**, **41** and **42**.	**S35-S37**
	^1H and ^{13}C-NMR spectra for **17-19**, **26-28**, **36**, **41** and **42**.	**S38-S55**

References **S56**

Supplementary Figures

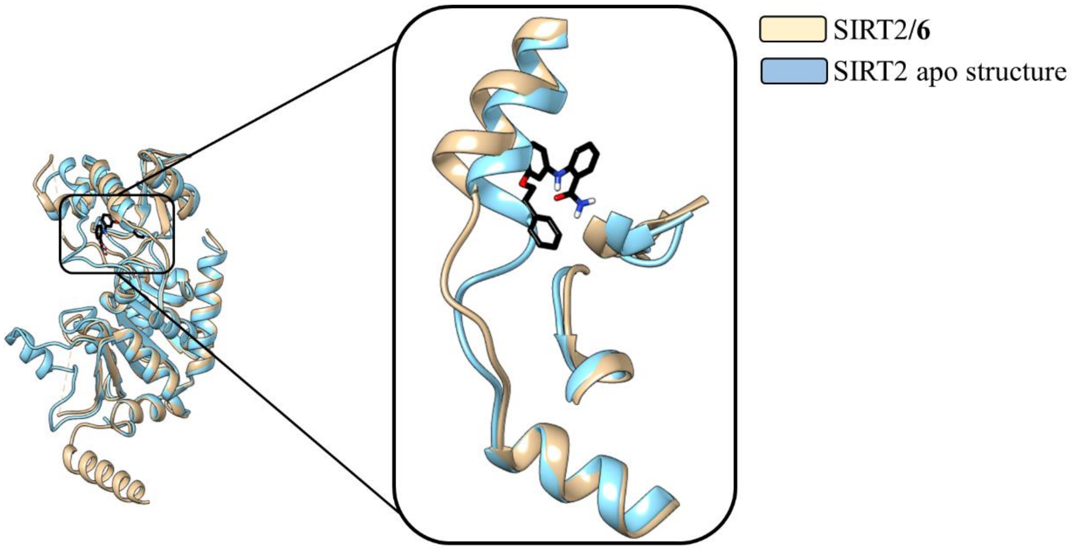

Fig. S1 Superimposition of SIRT2/**6** with the SIRT2 apo structure (PDB: 3ZGO). The α-helix and the connection loop shift induced by **6** are highlighted in the magnified window on the right, wherein **6** is colored in black.

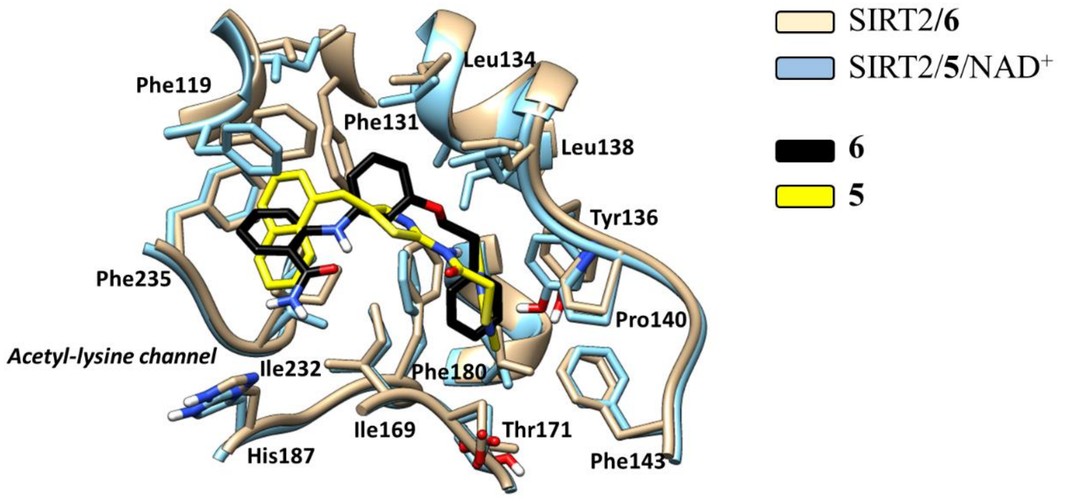

Fig. S2 Superimposition of SIRT2/**6** with the **5**/NAD^+ (PDB: 4RMG) structure. Only the inhibitor binding mode is shown.

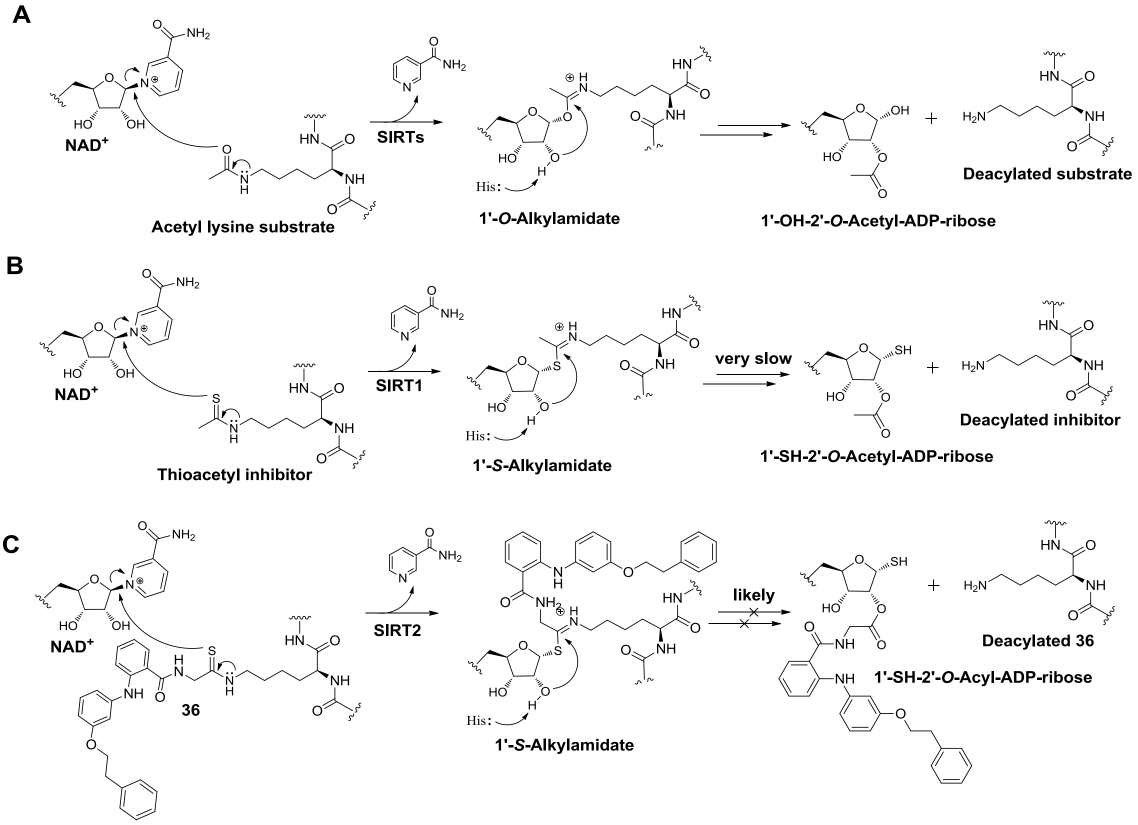

Fig. S3 Deacetylation mechanism catalyzed by sirtuins and inhibition mode of thioacetyl-based inhibitors, including **36**. (A) Simplified deacetylation mechanism catalyzed by sirtuins;[1,2] (B) inhibition mechanism on SIRT1 by a general substrate-competitive thioacetyl-based inhibitor;[3] (C) proposed SIRT2 inhibition mechanism by **36**.

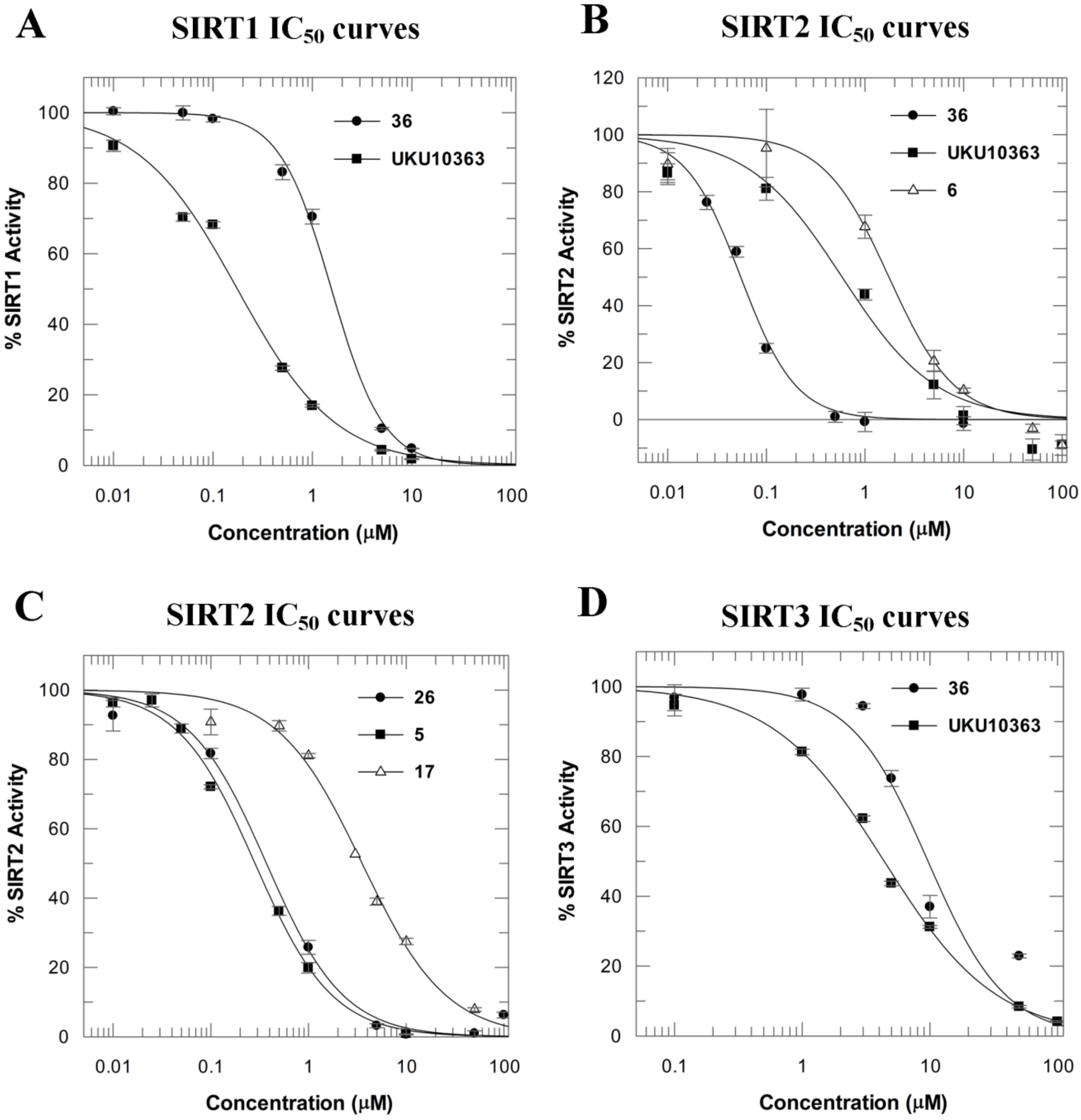

Fig. S4 IC_{50} curves for compounds **6**, **17**, **26**, **36**, UKU10363 and **5** on SIRT1-3. (A) SIRT1, **36**, and UKU10363; (B) SIRT2, **36**, UKU10363, and **6**; (C) SIRT2, **26**, **5**, and **17**; (D) SIRT3, **36**, and UKU10363. Fluor de Lys assay; values were calculated from three independent determinations, which afforded a total of 21 data points.

BML-AK556 instruction manual available with SIRT2 Fluorimetric Drug Discovery Kit provided by Enzo Life Sciences, was used as guideline to test the potential interference of SIRT2 inhibitors with the Fluor de Lys Developer II or the fluorescence signal. **26** and **36** (10 µL) in buffer/DMSO were added to selected wells (buffer/DMSO and buffer/DMSO/compound were added to the control and blank wells, respectively), followed by 40 µL of a solution containing the deacetylated standard (final concentration: 5 µM). After gentle mixing, the reaction was started upon addition of 50 µL of the Developer II solution to all wells except for the blank. Fluorescence readings were obtained from 0–30 min at 30 °C using a Victor X3 plate reader (λ_{ex} = 355 nm, λ_{em} = 460 nm). Compound **6** and SirReal2 (**5**) were used as negative controls.

	**Buffer**	**Cpd***	**Developer II**	**Deac. standard**
**Blank**	50 µL	10 µL	-	40 µL
**Reaction**	-	10 µL	50 µL	40 µL
*** Control blank contains DMSO/buffer**				

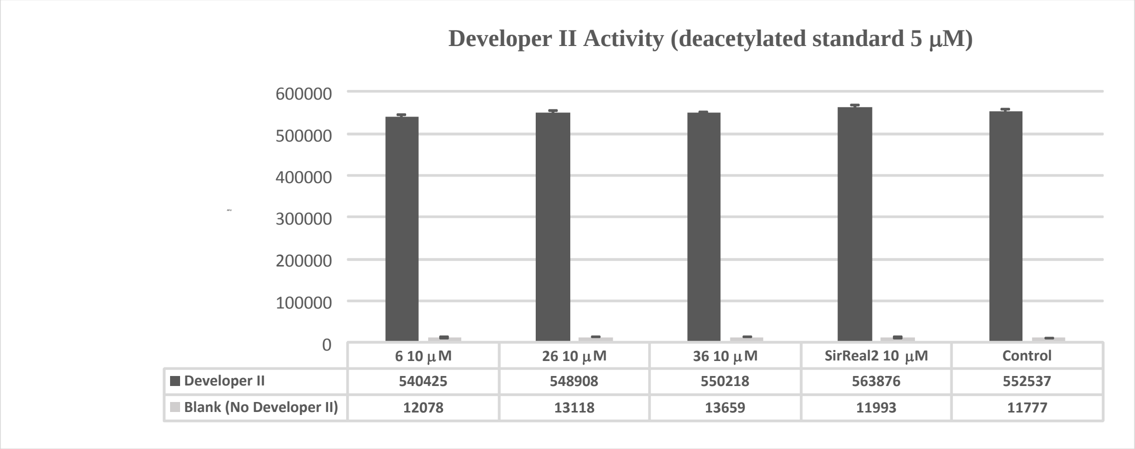

Fig. S5 Evaluation of the interference of **26** and **36** with the Developer II reaction on the SIRTs assay. Values were calculated from two independent determinations ± SD.

HPLC stability of 36 under SIRTs assay conditions

Compound **36** (10 µL, 0.5 mM) in DMSO/buffer (50 mM Tris/HCl, pH = 8.0, 137 mM NaCl, 2.7 mM KCl, 1 mM $MgCl_2$) was added to selected wells, followed by 40 µL of buffer (50 mM Tris/HCl, pH = 8.0, 137 mM NaCl, 2.7 mM KCl, 1 mM $MgCl_2$). The obtained solution was incubated at 30 °C and 250 rpm for 0, 50, 100, 150, and 200 min. At the end of each incubation time, the wells were diluted with CH_3CN (50 µL, HPLC grade). The entire volume (100 µL) was transferred to a Cosmospin filter H (0.45 µm) and 20 µL of the filtrate were injected in the HPLC instrument. Column: COSMOSIL 5C18-ARII (4.6 × 150 mm). Gradient: 0.1%TFA B, 0–20 min (10–90%); 20–30 min (90%), 30–40 min (90–10%). The peak area was determined at 220 nm.

Blank

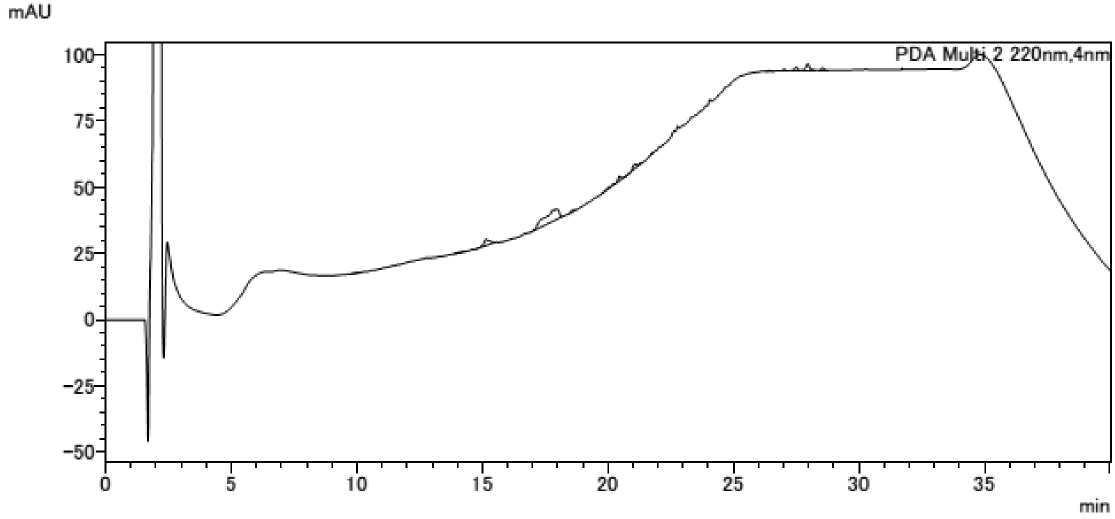

0 min

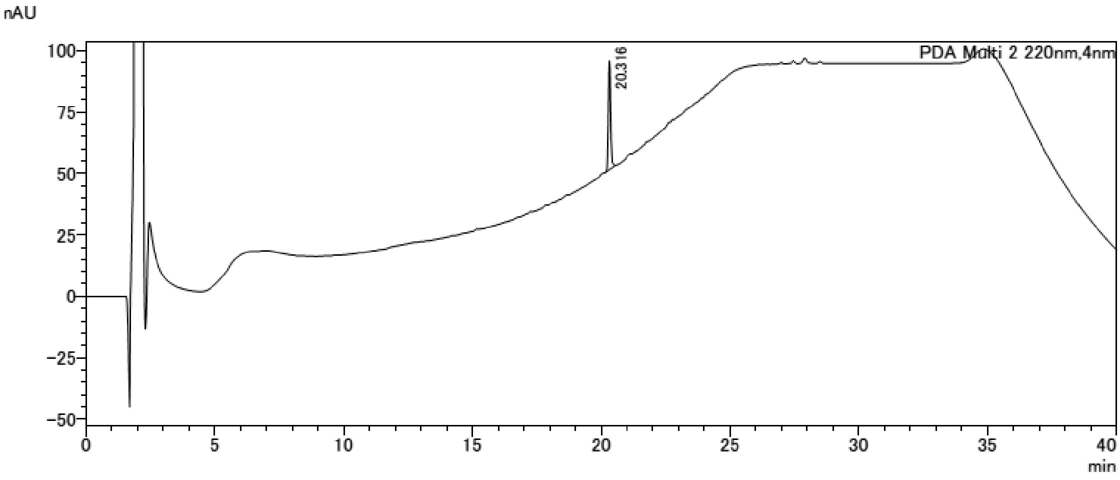

50 min

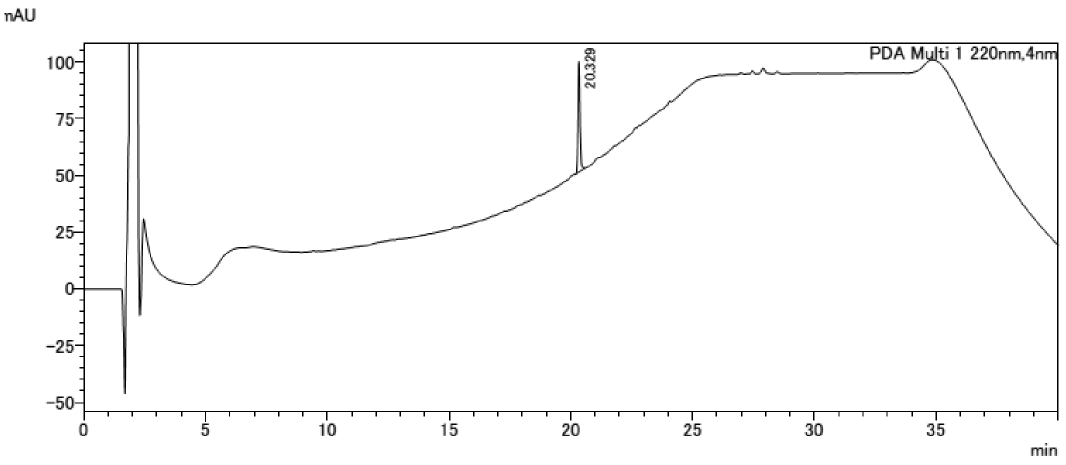

mAU
PDA Multi 1 220nm,4nm
20.329
min

100 min

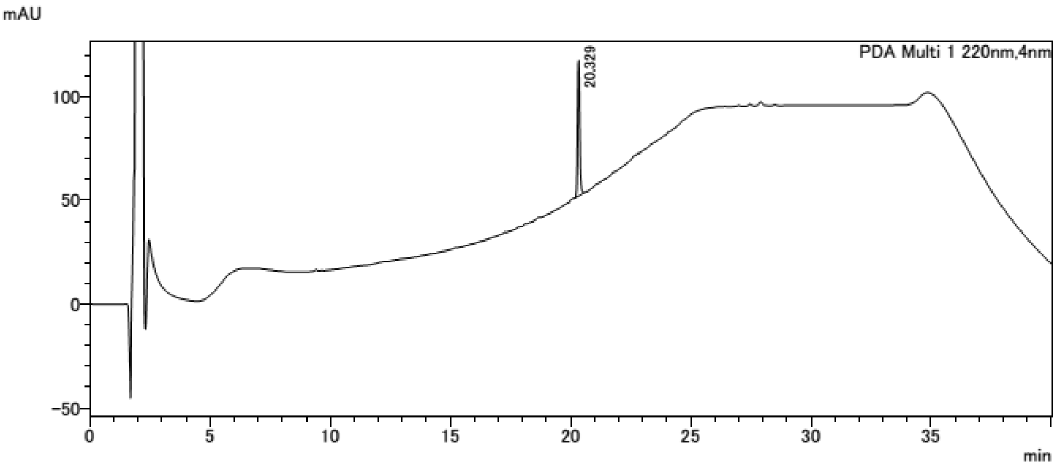

mAU
PDA Multi 1 220nm,4nm
20.329
min

150 min

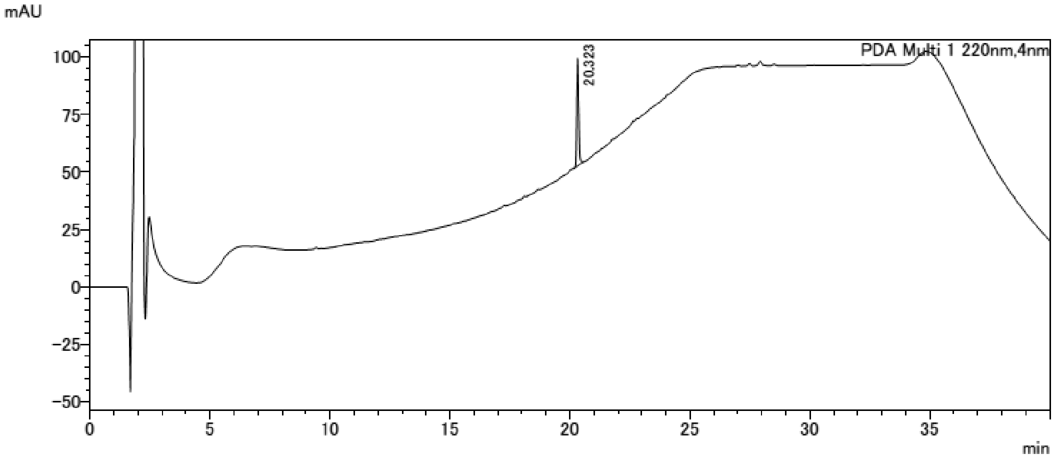

200 min

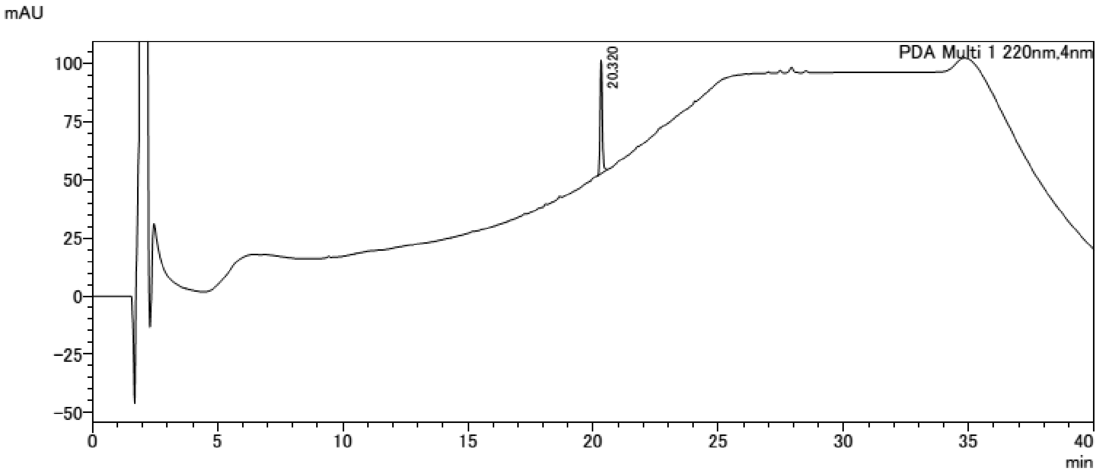

Fig. S6 HPLC stability of compound **36** under SIRTs assay conditions.

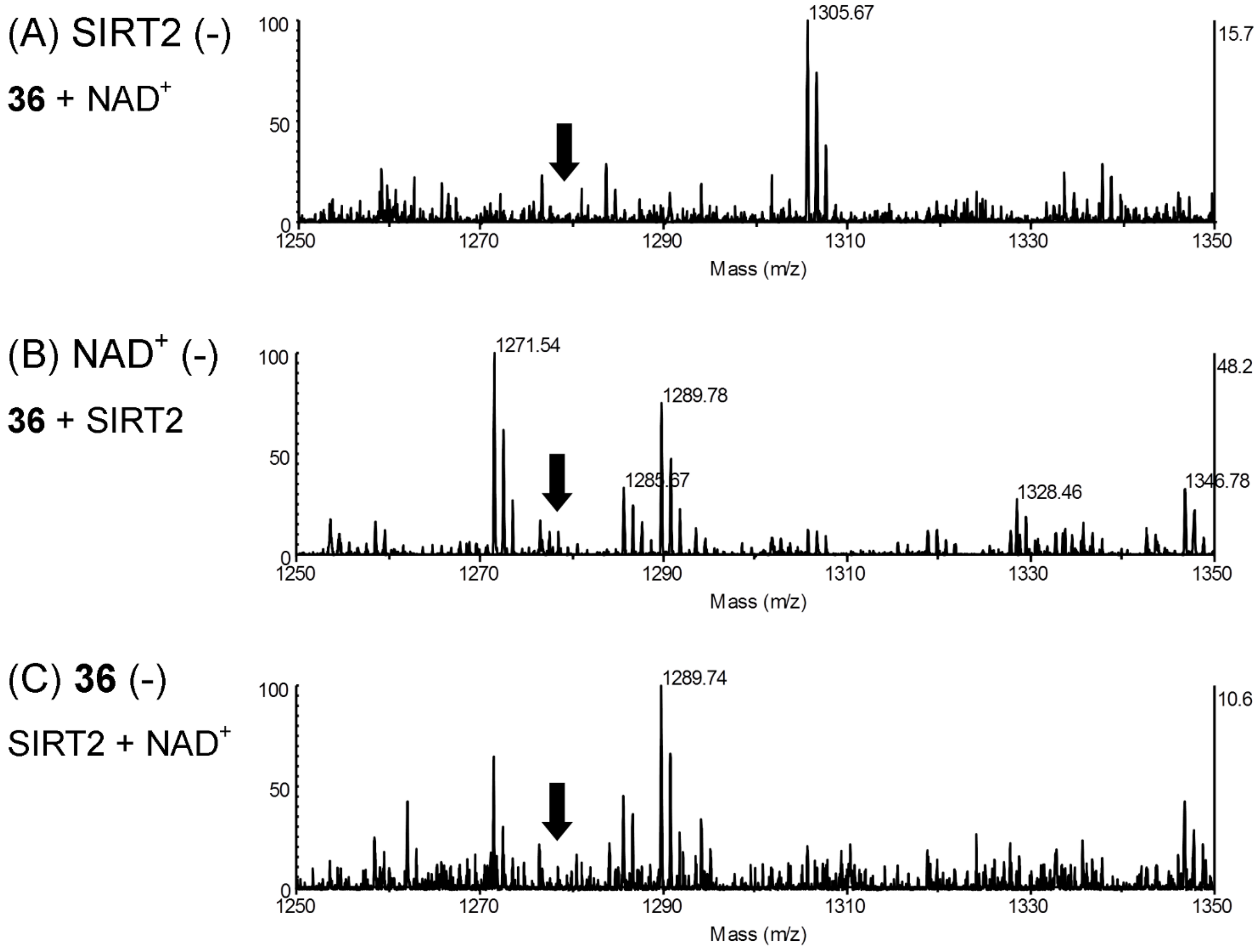

Fig. S7 MALDI-TOF blank spectra for the detection of the **36**-ADP-ribose conjugate. MALDI-TOF mass spectrometric detection of the ADP-ribose conjugate formed by **36** and NAD^+ in the absence of (A) SIRT2; (B) NAD^+, and (C) **36**.

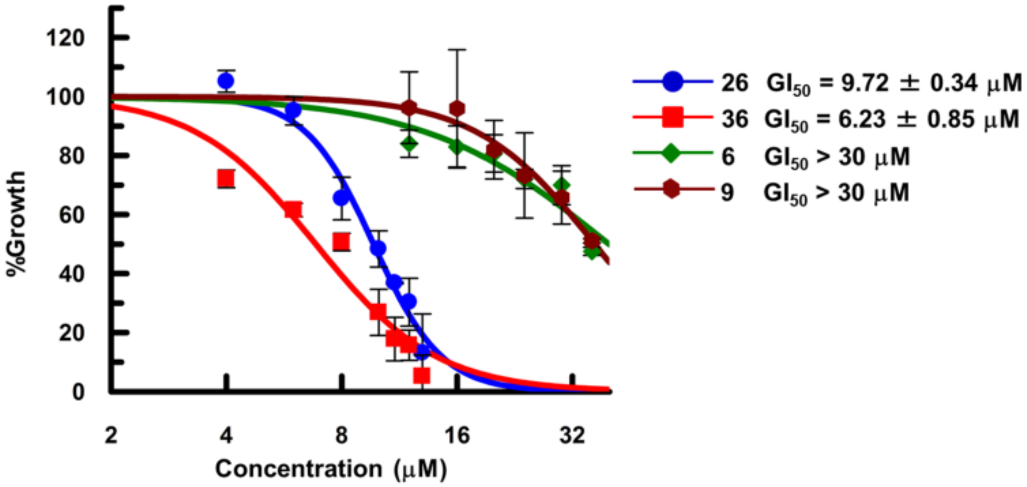

Fig. S8 Growth inhibition effect of **6**, **26**, **36** and EX-527 (**9**) in MCF-7 breast cancer cells. %Growth was evaluated after 72 h of treatment at different concentrations of **6**, **26**, **36** and **9**. The error bars represent the SD of three independent experiments.

Supplementary Table

Table S1. Data collection and refinement for the SIRT2/**6** crystal structure

Data collection			
Space group	P2221		
Cell dimension (Å)			
a, b, c (Å)	50.8503	57.9804	124.5321
α, β, γ (°)	90	90	90
Resolution (Å)	42.43-2.30	(2.38-2.30)	
R_{merge}	0.076	(0.280)	
l/σl	12.3	(4.7)	
Completeness (%)	94.9	(97.4)	
Redundancy	4.90	(4.94)	
Refinement			
Resolution (Å)	42.43-2.30		
No. of reflections	79310	(16183)	
R_{work}/R_{free} (%)	25.6/25.2		
No. of atoms			
Protein	2291		
Compound 6	25		
Zn^{2+}	1		
Water	136		
B-factor (Å^2)			
Protein	35.683		
Compound	39.212		
Zn^{2+}	47.400		
Water	40.482		
r.m.s. deviation			
Bond length (Å)	0.011		
Bond angles (°)	2.1		

Supplementary Methods

SIRT1–3 and SIRT5 Assays

Fluor de Lys assays were performed according to the method described in the Biomol kit sheets AK-555, 556, 557, and 513. The assays were carried out using acetylated substrates at concentrations of 25 μM (BML-KI177-0005 for SIRT1 and BML-KI179-0005 for SIRT2 and SIRT3) or 10 μM (BML-KI590-0050 for SIRT5); SIRT1 0.5–1 U/well (BML-SE239-0100), SIRT2 4–6 U/well (BML SE-251-0500), SIRT3 3-4 U/well (BML-SE270-0500), SIRT5 8 U/well (BML-SE555-9090), and NAD^+ 1 mM for SIRT1, SIRT2, and SIRT5, 1.8 mM for SIRT3; Developer II solution (BML-KI176-1250)/nicotinamide 1 mM (BML-KI283-0500) and sirtuin buffer (all provided as part of the kit). DMSO (purchased from Nacalai) was used at 2% to reach the final volume per well. 10 μL of compound in buffer/DMSO were added quickly to the selected wells (buffer/DMSO was added to the control and blank wells), followed by 25 μL of a buffer solution containing the substrate/NAD^+. After gentle mixing, the reaction was started by adding 15 μL of the diluted enzyme (15 μL of buffer was added to the blank wells). The reaction mixtures were incubated for 3 h at 30 °C (no rpm). Thereafter, 50 μL of a stop solution containing Fluor de Lys Developer II/nicotinamide were added to all wells and the fluorescence was measured for 0–30 min at 30 °C using a Victor X3 plate reader (λ_{ex} = 355 nm; λ_{em} = 460 nm). IC_{50} values were determined from three independent measurements affording a total of 21 data points. All data points were included in the IC_{50} calculation using GraFit 7.0.3, in which three independent curves were generated. Under the same assay conditions, **6**, UKU10363, and SirReal2 (**5**) were profiled and used as the internal standards.

Time-dependent inhibition of SIRT2 with 36

This assay was carried out using the SIRT2 acetylated substrate at a concentration of 130 μM (BML-KI179-0005), SIRT2 4 U/well (BML SE-251-0500), and NAD^+ 1 mM; Developer II solution (BML-KI176-1250)/nicotinamide 1 mM (BML-KI283-0500) and sirtuin buffer (all provided as part of the kit). DMSO (purchased from Nacalai) was used at 2% to reach the final volume per well. 10 μL of compound in buffer/DMSO were added quickly to the selected wells (buffer/DMSO was added to the control and blank wells), followed by 25 μL of a buffer solution containing the substrate/NAD^+. After gentle mixing, the reaction was started by adding 15 μL of the diluted enzyme (15 μL of buffer was added to the blank wells). The reaction mixtures were incubated for 0, 30, 60, 90, and 120 min (for the 0 min wells, Developer II was added immediately) at 30 °C directly in the plate reader. After the respective time, 50 μL of a stop solution containing Fluor de Lys Developer II/nicotinamide were

added to the wells and the fluorescence was measured for 0–20 min at 30 °C using a Victor X3 plate reader (λ_{ex} = 355 nm; λ_{em} = 460 nm). The results were plotted using GraFit 7.0.3.

SIRT2 substrate competition analysis for 36

The assay follows in general the same procedure reported for the SIRT2 assay (*vide supra*), except for the following changes: reaction time = 45 min; [NAD^+] = 2 mM; [substrate] = 50, 80, 150, or 300 µM.

Mass spectrometric detection of the ADP-ribose conjugate

Reactions were conducted for 5 min at 37 °C in 5 µL containing 1.9 µM SIRT2 (SignalChem), 500 µM NAD^+ or 6-AE-NAD^+ (BIOLOG Life Science Institute), and 1 mM **36**, as well as 40 mM sodium phosphate buffer (pH = 7.0), containing 240 mM NaCl, 120 mM imidazole, 0.08 mM PMSF, 0.2 mM DTT, 20% glycerol, and 2% DMSO. Controls were measured in the absence of compounds or the enzyme. The reaction mixtures were diluted with 5 µL of water and purified using ZipTip-µC_{18} (Millipore). The fraction eluted with 2 µL of 50% acetonitrile containing α-cyano-4-hydroxycinnamic acid at a concentration of 5 mg/mL was directly subjected to MALDI-TOF MS analysis. MALDI-TOF mass spectra were acquired on an AB SCIEX TOF/TOF™ 5800 (AB SCIEX) in reflectron negative ion mode.

Cell cultures

MCF-7 cells (RIKEN BRC via the National Bio-Resource Project of MEXT, Japan) were cultured in DMEM (high glucose; Nacalai, #08489-45) containing 10% fetal bovine serum (FBS; SIGMA, #172012-500ML), an antibiotic-antimycotic mixed stock solution (Nacalai, #09366-44), an L-glutamine stock solution (Nacalai, #16948-04), or a sodium pyruvate solution (Nacalai, #06977-34) at 37 °C in a humidified atmosphere of 5% CO_2 in air. Human breast cancer MDA-MB-231 cells (American type culture collection, ATCC) were cultured at 37 °C in Leibovitz's L-15 medium containing 2 mM of glutamine, 10% FBS, and a penicillin and streptomycin mixture. The Neuro-2a (N2a) cell line was obtained from the Japanese Collection of Research Bioresources (JCRB) Cell Bank. The N2a cell culture was performed according to previously reported procedures.[4]

Cell growth assay

MCF-7 and MDA-MB-231 cells were plated in 96-well plates (initial density: 1×10^3 cells per well) and incubated at 37 °C. After 24 h, test compound solutions (50 μL/well) of varying concentrations in medium (DMEM and Leibovitz's L-15 for MCF-7 and MDA-MB-231 cells respectively) for were added to the cells at 37 °C under 5% CO_2 in air and left to react for 72 h. Thereafter, the mixtures were treated with 10 μL of AlamarBlue® (AbD Serotec, #BUF012A), incubation was continued at 37 °C for 3 h. The fluorescence in each well was measured with an ARVOTM X3 microplate reader (λ_{ex} = 540 nm; λ_{em} = 590 nm). With the obtained fluorescence readings, it was possible to calculate the percentage of cell growth.

Western Blotting

MDA-MB-231 cells (5×10^5 cells/2 mL/dish) were treated for 6 h with the test compounds at the indicated concentrations in the cell culture medium, before the cells were collected and extracted with SDS buffer. The protein concentrations of the lysates were determined using a BCA protein assay. Equivalent amounts of protein from each lysate were resolved in 5–20% SDS-polyacrylamide gels and transferred onto PVDF membranes. After blocking with TBS-T containing 5% skimmed milk, the transblotted membranes were probed with the rabbit monoclonal H3K9Ac antibody (CST, #9649) (1:1000 dilution), rabbit polyclonal H3 antibody (Abcam, #ab1791) (1:200000 dilution), mouse monoclonal acetyl-α-tubulin antibody (Sigma, #T6793) (1:2000 dilution), or mouse monoclonal α-tubulin antibody (Sigma, #T8203) (1:2000 dilution) in TBS-T containing 5% skimmed milk. The probed membranes were washed three times with TBS-T, incubated with ECL rabbit IgG, HRP-linked whole antibody (GE Healthcare Life Sciences, #NA934) (1:2500 dilution), ECL mouse IgG, or HRP-linked whole antibody (GE Healthcare Life Sciences, #NA931) (1:2500 or 1:10000 dilution), and washed again three times with TBS-T. The immunoblots were visualized by enhanced chemiluminescence with the ImmobilonTM Western Chemiluminescent HRP Substrate (Millipore, #WBKLS0500).

Neurite outgrowth assay

N2a cells were plated at a concentration of 1×10^4 cell/mL in DMEM including high glucose, 10% FBS, 100 U/mL penicillin, and 100 μg/mL streptomycin at 37 °C in 5% CO_2 humidified atmosphere. For the differentiation study, the medium was changed to DMEM supplemented with 2% FBS. After incubation with or without **36** for 72 h, the cell morphology was examined using a microscope (Olympus CKX41) and further analyzed with the Photomeasure software (Kenis Ltd.). The

differentiated cells were defined as those with at least one neurite that was longer than twice the diameter of the cell body. The results are expressed as the percentage of differentiated cells relative to the total number of counted cells. These experiments were carried out in triplicate. One-way ANOVA and Dunnett's *post hoc* tests were used to determine the significance among the groups.

Crystallization, data collection and figures preparation

Crystals of the purified SIRT2Tm protein (34-356) in complex with **6** (final concentration: 10 mg/mL) were obtained using 0.1 M Bis-Tris buffer (pH = 5.5) and 15% (w/v) PEG 5000 MME at 16 °C. X-ray diffraction data were collected at 100 K on the BL41XU beamline at Spring-8 (Hyogo, Japan) and processed using HKL2000 (HKL Research). Structure refinements were carried out using Discovery Studio (BIOVIA). Figures were prepared with UCSF Chimera 1.10.2, a visualization system for exploratory research and analysis.[5]

Chemical synthesis of 10–42

General. The chemical reagents and solvents used in this study were of commercially available high purity. Reagents and solvents were purchased from Sigma Aldrich, Wako Pure Chemical Industries, and TCI Tokyo Chemical Industry CO, LTD. Organic solvents were dried over anhydrous sodium sulfate. Compound **6** and UKU10363 were prepared according to procedures reported by Suzuki *et al.*[6] and Mellini *et al.*,[7] respectively. SirReal2 (**5**)[8] and EX-527 (**9**)[9] were purchased from Sigma Aldrich. NMR spectra were recorded on a Bruker Avance 300 AV (Bruker Biospin, Swizerland) spectrometer operating at 300.1 MHz (^{1}H) or 75.5 MHz (^{13}C). The chemical shift values are reported as δ (ppm) relatively to TMS (tetramethylsilane) as the internal reference (δ = 0), whereby coupling constants are given in Hz. Positive/negative LRMS ion mass spectra were recorded on a Bruker HCT-Plus. The purity of all tested compounds was determined by HPLC using a Shimadzu UFLC (SPD-M20A UV detector, DGU-20A3R degassing unit, LC-20AD solvent delivery unit and CBM-20A system) and a C18 column (Inert Sustain, 4.6*150, 5 μM), UV detection (λ = 220 or 254 nm), and a flow of 1 mL/min. HPLC conditions: eluent A: H_2O containing 0.1% TFA; eluent B: acetonitrile

containing 0.1% TFA. Gradient: B: 0 to 20 min, 10–90%; 20 to 30 min, 90%; 30 to 40 min, 90–10%. Melting points were determined using a Yanako Micro Melting Point apparatus. High-resolution mass spectra (HRMS) were recorded on a JEOL JMS-SX102A mass spectrometer.

Scheme S1.[a]

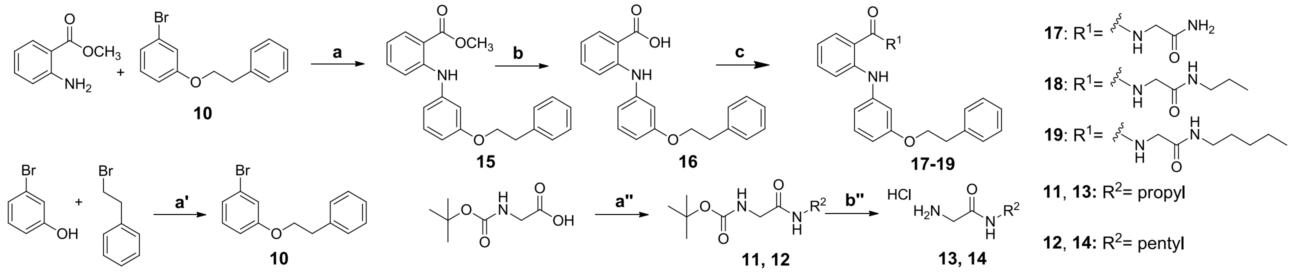

[a]Reagents and conditions: a) Pd_2dba_3, 2-dicyclohexylphosphino-2’,4’,6’-triisopropyl biphenyl (XPhos), K_2CO_3, *t*-BuOH, reflux, 18 h; b) THF:MeOH:H_2O, LiOH, rt, overnight, then 1 N aqueous HCl to establish pH = 2; c, a’’) dry DMF, amine, EDCI·HCl, anhydrous HOBT, TEA, N_2 flow, 0 °C to rt, 15–17 h; a’) dry acetone, K_2CO_3, N_2 atmosphere, reflux 48 h; b’’) 2-propanol, 37% aqueous HCl, triisopropylsilane, rt to 50 °C, 2 h.

Scheme S2.[a]

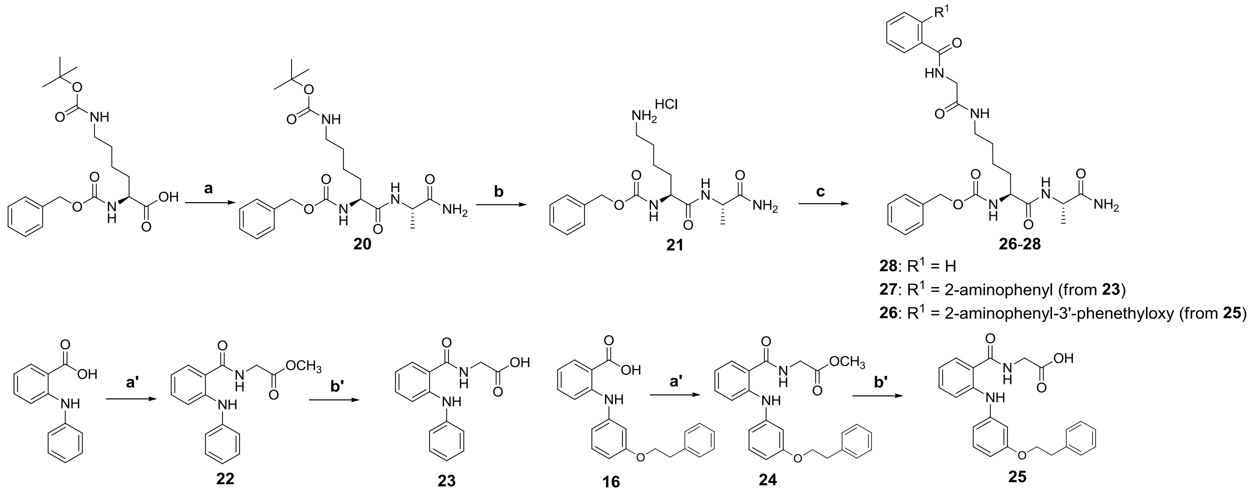

[a]Reagents and conditions: a) dry DMF, TEA, COMU, alaninamide hydrochloride, rt, 5 h, N_2 flow; b) dry DCM, 4 N HCl in dioxane, 0 °C to rt, 200 min; c, a’) dry DMF, carboxylic acid or amine, EDCI·HCl, anhydrous HOBT, TEA, N_2 flow, 0 °C to rt, 8.5–20 h; b’) THF:MeOH:H_2O, LiOH, rt, 150 min, then 1 N aqueous HCl to establish pH = 2.

Scheme S3.[a]

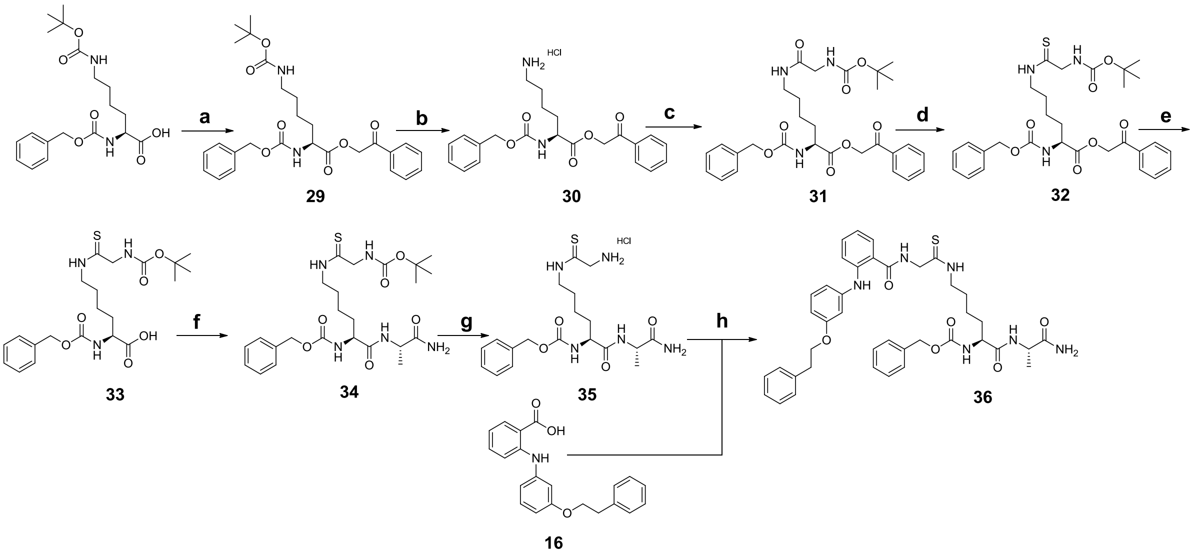

[a]Reagents and conditions: a) EtOAc, TEA, rt 16 h; b) dry DCM, 4 N HCl in dioxane, 0 °C to rt, 280 min; c, h) dry DMF, acid, EDCI·HCl, anhydrous HOBT, TEA, 0 °C to rt, 5–25 h; d) dry toluene, Lawesson's reagent, 60 °C, 6 h; e) THF:MeOH:H_2O, LiOH, rt, 4 h, then 1 N aqueous HCl to establish pH = 2; f) dry DMF, TEA, COMU, alaninamide hydrochloride, 0 °C to rt, 22 h, N_2 flow; g) 2-propanol, 37% HCl, triisopropylsilane, rt to 50 °C, 160 min.

Scheme S4.[a]

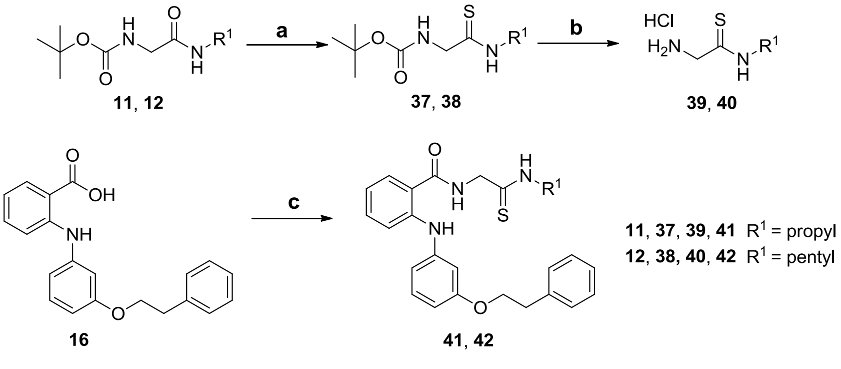

[a]Reagents and conditions: a) dry toluene, Lawesson's reagent, 60 °C, 210 min; b) 2-propanol, 37% HCl, triisopropylsilane, rt to 50 °C, 120 min; c) dry DMF, **39** or **40**, EDCI·HCl, anhydrous HOBT, TEA, N_2 flow, 0 °C to rt, 15–17 h.

Synthesis of 10–42

1-bromo-3-phenethoxybenzene (10). K_2CO_3 (4.70 g, 34 mmol) and (2-bromoethyl)benzene (4.59 mL, 34 mmol) were added to a solution of 3-bromophenol (2.0 g, 11.5 mmol) in dry acetone (8 mL). The reaction was stirred overnight under an N_2 atmosphere while being heated to reflux. Stirring was then continued for 48 h at rt. Thereafter, the resulting inorganic precipitate was filtered off and the solvent evaporated. The thus obtained yellow oily residue was purified by column chromatography on silica Kieselgel 60 with *n*-hexane:EtOAc (40/1) as the eluent to afford a colorless oil (2.98 g, 10.7 mmol, 93.5%). R*f* = 0.26 (*n*-hexane:EtOAc = 40:1). ^{1}H-NMR (DMSO-d_6): δ = 7.30–7.18 (m overlap, 4H), 7.13–7.07 (m overlap, 2H), 6.94–6.91 (m, 1H), 4.19 (t, 2H, *J* = 6.80 Hz), 3.00 (t, 2H, *J* = 6.80 Hz). ^{13}C-NMR (DMSO-d_6): δ = 159.37, 138.08, 131.07, 128.85, 128.21, 126.21, 123.35, 122.04, 117.30, 113.87, 68.38, 34.73.

General procedure for the synthesis of 11 and 12. Example: *tert*-butyl [2-oxo-2-(propylamino)ethyl]carbamate (11). Propylamine (0.47 mL, 5.7 mmol), *N*-(3-dimethylaminopropyl)-*N′*-ethylcarbodiimide hydrochloride (EDCI·HCl) (1.62 g, 8.5 mmol), anhydrous 1-hydroxybenzotriazole (HOBT) (1.15 g, 8.5 mmol), and triethylamine (TEA) (3.48 mL, 25 mmol) were added to a solution of Boc-Gly-OH (1.0 g, 5.7 mmol) in dry DMF (12 mL) in an ice bath. The reaction was stirred under an N_2 atmosphere for 15 h, before brine (50 mL) was added and the reaction mixture was extracted with EtOAc (4 × 50 mL). The combined organic phases were washed with a saturated aqueous $NaHCO_3$ solution (40 mL) and brine (40 mL), dried over Na_2SO_4, and the solvent was evaporated under reduced pressure. The residue was purified by flash column chromatography on silica using EtOAc:*n*-hexane (1:1), followed by EtOAc as the eluent to furnish a waxy solid (1.01 g, 4.6 mmol, 81.9%). R*f* = 0.17 (*n*-hexane:EtOAc = 1:1) visualized with phosphomolybdic acid. ^{1}H-NMR (DMSO-d_6): δ = 7.69 (s br, 1H), 6.87 (t, 1H, *J* = 6.04 Hz), 3.49 (d, 2H, *J* = 6.04 Hz), 3.04–2.98 (m, 2H), 1.43–1.33 (m overlap, 11H), 0.83 (t, 3H, *J* = 7.36 Hz). ^{13}C-NMR (DMSO-d_6): δ = 169.05, 155.73, 77.94, 43.27, 40.24, 28.15, 20.69, 11.26. ESI-MS (*m/z*): 217.1 $[M + H]^+$, 239.2 $[M + Na]^+$ (Chemical Formula: $C_{10}H_{20}N_2O_3$; Molecular Weight: 216.2774).

***tert*-Butyl [2-oxo-2-(pentylamino)ethyl]carbamate (12).** Waxy solid (1.34 g, 5.5 mmol, 96.5%). R*f* = 0.27 (*n*-hexane:EtOAc = 1:1) visualized with phosphomolybdic acid. ^{1}H-NMR (DMSO-*d*$_6$): δ = 7.67 (s br, 1H), 6.87 (t, 1H, *J* = 5.85 Hz), 3.47 (d, 2H, *J* = 6.23 Hz), 3.07–3.00 (m, 2H), 1.43–1.32 (m overlap, 11H), 1.31–1.18 (m, 4H), 0.85 (t, 3H, *J* = 7.18 Hz). ^{13}C-NMR (DMSO-*d*$_6$): δ = 168.98, 155.70, 77.91, 43.26, 38.38, 28.77, 28.50, 28.12, 21.8, 13.80. ESI-MS (*m/z*): 245.2 $[M + H]^+$, 267.2 $[M + Na]^+$ (Chemical Formula: $C_{12}H_{24}N_2O_3$; Molecular Weight: 244.3306).

General procedure for the synthesis of 13 and 14. Example: 2-amino-*N*-propylacetamide hydrochloride 13. Triisopropylsilane (0.25 mL, 1.2 mmol) was added to a stirred solution of **11** (0.2 g, 0.92 mmol) in 2-propanol (7 mL), followed by 37% aqueous HCl (0.21 mL). Then the reaction mixture was warmed to 50 °C. After 20 min and 40 min, 0.21 mL of 37% aqueous HCl were added, and the solution was stirred for 1 h at 50 °C. Thereafter, 2-propanol was removed under reduced pressure and the sticky residue washed with Et_2O (3 × 5 mL), EtOAc (2 × 5 mL), and Et_2O (3 × 5 mL) to afford a light white solid (0.106 g, 0.69 mmol, 75.5%). ^{1}H-NMR (DMSO-*d*$_6$): δ = 8.48 (t, 1H, *J* = 5.10 Hz), 8.18 (s, 3H), 3.50 (s, 2H), 3.10–3.04 (m, 2H), 1.49–1.37 (m, 2H), 0.86 (t, 3H, *J* = 7.37 Hz). ^{13}C-NMR (DMSO-*d*$_6$): δ =165.58, 40.45, 40.00, 22.13, 11.37. ESI-MS (*m/z*): 117.3 $[M + H]^+$ (Chemical Formula: $C_5H_{12}N_2O$; Molecular Weight: 116.1616).

2-Amino-*N*-pentylacetamide hydrochloride (14). From **12** (0.22 g, 0.92 mmol). White solid (0.10 g, 0.55 mmol, 60.1%). ^{1}H-NMR (DMSO-*d*$_6$): 8.45 (t, 1H, *J* = 5.29 Hz), 8.15 (s, 3H), 3.49 (s, 2H), 3.13–3.06 (m, 2H), 1.44–1.37 (m, 2H), 1.29–1.25 (m, overlap, 4H), 0.86 (t, 3H, *J* = 6.99 Hz). ^{13}C-NMR (DMSO-*d*$_6$): δ = 165.53, 40.01, 38.62, 28.50(2C), 21.76, 13.83. ESI-MS (*m/z*): 145.2 $[M + H]^+$ (Chemical Formula: $C_7H_{16}N_2O$; Molecular Weight: 144.2147).

Methyl 2-[(3-phenethoxyphenyl)amino]benzoate (15). A mixture of methyl-2-aminobenzoate (0.59 mL, 4.5 mmol), **10** (1.05 g, 3.8 mmol), K_2CO_3 (0.73 g, 5.3 mmol), Pd_2dba_3 (0.32 g, 0.35 mmol) and XPhos (0.36 g, 0.76 mmol) in *t*-BuOH (20 mL) under a flow of N_2 flow was heated to reflux for 18 h. Then, EtOAc (40 mL) was added and the resulting suspension filtered. The obtained solution was evaporated and the residue purified by column chromatography on silica Kieselgel 60 using *n*–

hexane:EtOAc (35/1) as the eluent to afford a brownish oil (1.28 g, 3.68 mmol, 96.9%). R*f* = 0.24 (*n*–hexane:EtOAc = 35:1). ^{1}H-NMR ($CDCl_3$): δ = 9.44 (s, 1H), 7.96 (m, 1H), 7.34–7.18 (m overlap, 8H), 6.84–6.78 (m overlap, 2H), 6.75–6.70 (m, 1H), 6.63 (dd, 1H, *J* = 8.12, 0.57 Hz), 4.14 (t, 2H, *J* = 7.18 Hz), 3.88 (s, 3H), 3.08 (t, 2H, *J* = 7.18 Hz). ^{13}C-NMR ($CDCl_3$): δ = 168.90, 159.84, 147.69, 142.10, 138.23, 134.10, 131.61, 130.03, 129.02, 128.50, 126.51, 117.26, 114.69, 114.49, 112.14, 109.68, 108.58, 68.72, 51.78, 35.80. ESI-MS (*m*/*z*): 348.2 (Chemical Formula: $C_{22}H_{21}NO_3$; Molecular Weight: 347.4070).

2-[(3-phenethoxyphenyl)amino]benzoic acid (16). A solution of LiOH (1.1 g, 26 mmol) in H_2O (8 mL) was added to a solution of **15** (1.20 g, 3.4 mmol) in THF:MeOH (16 mL/8 mL) and the reaction mixture was stirred at room temperature overnight. Then, the solvent volume was halved under vacuum and H_2O (20 mL) was added, before pH = 2 was established using a 1 N aqueous HCl solution. The product was extracted with EtOAc (4 × 60 mL) and the combined organic phases were washed with H_2O (30 mL). The oily residue was washed with *n*-hexane (2 × 3 mL) in order to remove any unreacted ester to afford a light yellow solid (1.1 g, 3.3 mmol, 97%). ^{1}H-NMR (DMSO-d_6): δ = 13.05 (s br, 1H), 9.57 (s br, 1H), 7.88 (dd, 1H, *J* = 7.93, 1.70 Hz), 7.41–7.36 (m, 1H), 7.33–7.17 (m overlap, 7H), 6.81–6.75 (m overlap, 3H), 6.61 (dd, 1H, *J* = 7.55, 2.27 Hz), 4.17 (t, 2H, *J* = 6.80 Hz), 3.01 (t, 2H, *J* = 6.80 Hz). ^{13}C-NMR (DMSO-d_6): δ = 169.88, 159.49, 146.73, 141.91, 138.38, 134.09, 131.85, 130.20, 128.93, 128.28, 126.24, 117.59, 114.31, 113.34, 112.96, 109.27, 107.23, 68.13, 34.95. ESI-MS (*m*/*z*): 331.9 $[M - H]^-$; 334.1 $[M + H]^+$ (Chemical Formula: $C_{21}H_{19}NO_3$; Molecular Weight: 333.3805).

General procedure for the synthesis of 17, 18 and 19. Example: *N*-(2-amino-2-oxoethyl)-2-[(3-phenethoxyphenyl)amino]benzamide (17). Glycinamide hydrochloride (0.026 g, 0.24 mmol), EDCI·HCl (0.069 g, 0.36 mmol), anhydrous HOBT (0.048 g, 0.36 mmol), and TEA (0.15 mL, 1.08 mmol) were added sequentially under a flow of N_2 to a solution of **16** (0.08 g, 0.24 mmol) in dry DMF (3 mL) in an ice bed. The reaction was stirred for 17 h, after which brine (~30 mL) was added

and the product was extracted with EtOAc (4 × 20 mL). The combined organic phases were washed with brine, dried over Na_2SO_4, and the solvent was removed under reduced pressure. The residue was purified by column chromatography on silica Kieselgel 60 using EtOAc:*n*-hexane (8:2), followed by EtOAc as the eluent to furnish a white solid (0.052 g, 0.13 mmol, 55.6%). R*f* = 0.18 (EtOAc:*n*-hexane = 8:2). m.p. 131–133 °C. ^{1}H-NMR (DMSO-*d*$_6$): δ = 9.55 (s, 1H), 8.69 (t, 1H, *J* = 5.85 Hz), 7.68 (d, 1H, *J* = 7.55 Hz), 7.41–7.15 (m overlap, 9H), 7.07 (s br, 1H), 6.87–6.82 (m, 1H), 6.74–6.69 (m overlap, 2H), 6.54 (dd, 1H, *J* = 8.12, 1.89 Hz), 4.17 (t, 2H, *J* = 6.80 Hz), 3.80 (d, 2H, *J* = 5.85 Hz), 3.02 (t, 2H, *J* = 6.80 Hz). ^{13}C-NMR (MeOD + 6% DMSO-*d*$_6$): δ =174.36, 171.70, 161.28, 145.78, 144.59, 139.9, 133.23, 131.10, 130.05, 129.90, 129.43, 127.38, 120.65, 119.76, 117.27, 113.35, 109.35, 107.25, 69.77, 43.41, 36.68. ESI-MS (*m*/*z*): 390.4 $[M + H]^+$. HPLC: purity 98.88% at 254 nm, t_R = 18.33 min. HRMS (EI) calcd for $C_{23}H_{23}N_3O_3$, 389.17395; found, 389.17387.

***N*-[2-Oxo-2-(propylamino)ethyl]-2-[(3-phenethoxyphenyl)amino]benzamide (18)**. **16** (0.08 g, 0.24mmol), **13** (0.037 g, 0.24 mmol), EDCI·HCl (0.069 g, 0.36 mmol), anhydrous HOBT (0.048 g, 0.36 mmol), and TEA (0.15 mL, 1.08 mmol). Purification by column chromatography on silica Kieselgel 60 using EtOAc:*n*-hexane (1:1) as the eluent to furnish a colorless sticky solid (0.046 g, 0.106 mmol, 44.4%). R*f* = 0.17 (EtOAc:*n*-hexane = 1:1). ^{1}H-NMR (DMSO-*d*$_6$): δ = 9.54 (s, 1H), 8.72 (t, 1H, *J* = 5.8 5Hz), 7.90 (t, 1H, *J* = 5.67 Hz), 7.68 (d, 1H, *J* = 7.37 Hz), 7.38–7.15 (m overlap, 8H), 6.88–6.82 (m, 1H), 6.74–6.69 (m overlap, 2H), 6.54 (dd, 1H, *J* = 7.74, 1.89 Hz), 4.16 (t, 2H, *J* = 6.80 Hz), 3.82 (d, 2H, *J* = 5.85 Hz), 3.06–3.00 (m overlap 4H), 1.47–1.35 (m, 2H), 0.83 (t, 3H, *J* = 7.37 Hz). ^{13}C-NMR (MeOD): δ = 171.86, 171.71, 161.30, 145.79, 144.72, 139.85, 133.23, 131.04, 130.00, 129.85, 129.39, 127.35, 120.88, 119.89, 117.57, 113.28, 109.37, 107.22, 69.81, 43.89, 42.22, 36.71, 23.58, 11.61. ESI-MS (*m*/*z*): 432.5 $[M + H]^+$. HPLC purity 98.94% at 254 nm, t_R: 20.63 min. HRMS (EI) calcd for $C_{26}H_{29}N_3O_3$, 431.22090; found, 431.22004.

***N*-[2-Oxo-2-(pentylamino)ethyl]-2-[(3-phenethoxyphenyl)amino]benzamide (19)**. **16** (0.08 g, 0.24 mmol), **14** (0.043 g, 0.24 mmol), EDCI·HCl (0.069 g, 0.36 mmol), anhydrous HOBT (0.048 g,

0.36 mmol), and TEA (0.15 mL, 1.08 mmol). Purification by column chromatography on silica Kieselgel 60 using EtOAc:*n*-hexane (1:1) as the eluent to furnish a colorless sticky solid (0.074 g, 0.16 mmol, 62.2%). R*f* = 0.25 (EtOAc:*n*-hexane = 1:1). ^{1}H-NMR (DMSO-*d*$_6$): δ = 9.56 (s, 1H), 8.71 (t, 1H, *J* = 6.04 Hz), 7.88 (t, 1H, *J* = 5.48 Hz), 7.69 (d, 1H, *J* = 8.31Hz), 7.38–7.15 (m overlap, 8H), 6.88–6.82 (m, 1H), 6.74–6.68 (m overlap, 2H), 6.53 (dd, 1H, *J* = 8.12, 1.89 Hz), 4.16 (t, 2H, *J* = 6.80 Hz), 3.81 (d, 2H, *J* = 5.67 Hz), 3.08–2.99 (m overlap, 4H), 1.44–1.35 (m, 2H), 1.31–1.20 (m overlap, 4H), 0.84 (t, 3H, *J* = 6.99 Hz). ^{13}C-NMR (MeOD): δ = 171.82, 171.61, 161.27, 145.81, 144.64, 139.80, 133.22, 131.01, 129.97, 129.81, 129.37, 127.32, 120.70, 119.82, 117.48, 113.30, 109.37, 107.27, 69.77, 43.91, 40.44, 36.69, 30.07, 30.03, 23.32, 14.29. ESI-MS (*m*/*z*): 460.5 $[M + H]^+$. HPLC: purity 99.35% at 254 nm, t_R: 22.27 min. HRMS (EI) calcd for $C_{28}H_{33}N_3O_3$, 459.25220; found, 459.25316.

Benzyl *tert*-butyl ((*S*)-6-{[(*S*)-1-amino-1-oxopropan-2-yl]amino}-6-oxohexane-1,5-diyl)dicarbamate (20). TEA (2.83 mL, 20.3 mmol) and (1-Cyano-2-ethoxy-2-oxoethylidenaminooxy)dimethylamino-morpholino-carbenium hexafluorophosphate (COMU) (1.49 g, 3.48 mmol) were added to a solution of Z-Lys(Boc)-OH (1.11 g, 2.9 mmol) in DMF (12 mL) under a flow of N_2, and the mixture was stirred for 1 min. Thereafter, L-alaninamide hydrochloride (0.4 g, 3.2 mmol) was added and the solution was stirred for 5 h at rt under a flow of N_2. Then, the reaction was quenched by adding brine (40 mL), before the obtained precipitate was filtered off and consecutively washed with a saturated aqueous solution of $NaHCO_3$ (40 mL), H_2O (40 mL), and Et_2O (5 × 10 mL). Compound **20** was obtained as a white solid (1.20 g, 2.7 mmol, 94%). R*f* = 0.47 (CH_2Cl_2:MeOH = 9:1 visualized with ninhydrin) ^{1}H-NMR (DMSO-*d*$_6$): δ = 7.86 (d, 1H, *J* = 7.55 Hz), 7.44–7.30 (m, 7H), 7.00 (s br, 1H), 6.76 (t, 1H, *J* = 5.29 Hz), 5.01 (s, 2H), 4.23–4.14 (m, 1H), 3.97–3.90 (m, 1H), 2.92–2.84 (m, 2H), 1.64–1.18 (m overlap, 19H). ^{13}C-NMR (DMSO-*d*$_6$): δ = 174.03, 171.42, 156.00, 155.53, 136.99, 128.29, 127.71, 127.58, 77.31, 65.36, 54.76(2C), 47.83, 31.43, 28.24(2C), 22.76, 18.41. ESI-MS (*m*/*z*): 451.4 $[M + H]^+$, 473.4 $[M + Na]^+$ (Chemical Formula: $C_{22}H_{34}N_4O_6$; Molecular Weight: 450.5286).

Benzyl ((*S*)-6-amino-1-{[(*S*)-1-amino-1-oxopropan-2-yl]amino}-1-oxohexan-2-yl)carbamate hydrochloride (21). HCl in dioxane (4 N, 3.51 mL) was slowly added to a stirred suspension of **20** (1.0 g, 2.2 mmol) in dry CH_2Cl_2 (17.5 mL), which was cooled in an ice bed. The reaction was then warmed to room temperature, where stirring was continued for 200 min. Thereafter, the solvents were evapored and the resulting residue washed with $CHCl_3$:Et_2O (1:1, 4 × 10 mL) to give a light yellow solid (0.85 g, 2.2 mmol, 100%). ^{1}H-NMR (DMSO-d_6): δ = 7.99–7.86 (m overlap, 4H), 7.44–7.30 (m, 6H), 7.02 (s br, 1H), 5.01 (s, 2H), 4.23–4.16 (m, 1H), 3.96–3.94 (m, 1H), 2.80–2.65 (m, 2H), 1.77–1.75 (m, 4H), 1.41–1.19 (m, 5H). ^{13}C-NMR (DMSO-d_6): δ = 174.20, 171.36, 155.99, 136.98, 128.32, 127.75, 127.61, 65.41, 54.54, 48.01, 38.44, 31.10, 26.42, 22.30, 18.39. ESI-MS (*m*/*z*): 351.2 $[M + H]^+$ (Chemical Formula: $C_{17}H_{26}N_4O_4$; Molecular Weight: 350.4127).

Methyl 2-[2-(phenylamino)benzamido]acetate (22). Compound **22** was prepared following the coupling procedure described for **17**: *N*-phenylanthranilic acid (0.6 g, 2.8 mmol), DMF (7 mL), glycine methyl ester (0.35 g, 2.8 mmol), EDCI·HCl (0.8 g, 4.2 mmol), anhydrous HOBT (0.57 g, 4.2 mmol), and TEA (1.75 mL, 13 mmol); reaction time: 8.5 h; extracted with EtOAc (4 × 50 mL) and washed once with a saturated aqueous solution of $NaHCO_3$ (20 mL). Purification by column chromatography on silica Kieselgel 60 using *n*-hexane:EtOAc (7:3) as the eluent to furnish a light yellow solid (0.62 g, 2.2 mmol, 78.6%). R*f* = 0.32 (*n*-hexane:EtOAc = 7:3). ^{1}H-NMR (DMSO-d_6): δ = 9.64 (s, 1H), 9.01 (t, 1H, *J* = 5.67 Hz), 7.69 (dd, 1H, *J* = 7.93, 1.32 Hz), 7.38–7.27 (m overlap, 4H), 7.17 (dd, 2H, *J* = 7.37, 1.13 Hz), 7.01–6.97 (m, 1H), 6.84 (td, 1H, *J* = 7.93, 1.51 Hz), 4.0 (d, 2H, *J* = 5.85 Hz), 3.66 (s, 3H). ^{13}C-NMR (DMSO-d_6): δ = 170.25, 169.22, 144.64, 141.21, 132.24, 129.34, 128.80, 121.98, 119.77, 117.89, 117.41, 114.73, 51.71, 41.02. ESI-MS (*m*/*z*): 285.1 $[M + H]^+$ (Chemical Formula: $C_{16}H_{16}N_2O_3$; Molecular Weight: 284.3098).

2-[2-(Phenylamino)benzamido]acetic acid (23). Compound **23** was prepared following the procedure reported for **16**: **22** (0.5 g, 1.7 mmol), THF:MeOH (6.25 mL/3.12 mL), LiOH·H_2O (0.51 g, 12 mmol), and H_2O (3.12 mL); reaction time: 4 h. The obtained white solid (0.44 g, 1.6 mmol,

95%) was used in the next step without further purification. ^{1}H-NMR (DMSO-d_6): δ = 9.65 (s, 1H), 8.88 (t, 1H, J = 5.67 Hz), 7.70 (dd, 1H, J = 7.74, 1.32 Hz), 7.34–7.27 (m, overlap, 4H), 7.18–7.15 (m, 2H), 7.01–6.96 (m, 1H), 6.84 (td, 1H, 7.93, 1.70 Hz), 3.91 (d, 2H, J = 5.85 Hz). ^{13}C-NMR (DMSO-d_6): δ = 171.24, 169.12, 144.48, 141.29, 132.08, 129.35, 128.77, 121.89, 119.65, 117.92, 117.85, 114.70, 41.06. ESI-MS (m/z): 271.1 $[M + H]^+$; 268.9 $[M - H]^-$ (Chemical Formula: $C_{15}H_{14}N_2O_3$; Molecular Weight: 270.2833).

Methyl 2-{2-[(3-phenethoxyphenyl)amino]benzamido}acetate (24). Compound **24** was prepared following the coupling procedure reported for **17**: **16** (0.2 g, 0.6 mmol), glycine methyl ester hydrochloride (0.075 g, 0.6 mmol), EDCI·HCl (0.172 g, 0.9 mmol), anhydrous HOBT (0.12 g, 0.9 mmol), and TEA (0.38 mL, 2.7 mmol). Purification by column chromatography on silica Kieselgel 60 using EtOAc:*n*-hexane (3:7) as the eluent to furnish a sticky solid (0.22 g, 0.5 mmol, 83.3%). R*f* = 0.29 (EtOAc:*n*-hexane = 3:7). ^{1}H-NMR (DMSO-d_6): δ = 9.57 (s, 1H), 8.99 (t, 1H, J = 5.48 Hz), 7.70 (d, 1H, J = 8.88 Hz), 7.39–7.16 (m overlap, 8H), 6.88–6.83 (m, 1H), 6.74–6.69 (m overlap, 2H), 6.56 (dd, 1H, J = 8.31, 1.89 Hz), 4.17 (t, 2H, J = 6.80 Hz), 4.00 (d, 2H, J = 5.67 Hz), 3.65 (s, 3H), 3.02 (t, 2H, J = 6.80 Hz). ^{13}C-NMR (MeOD): δ = 172.15, 171.94, 161.30, 145.98, 144.48, 139.85, 133.30, 131.04, 130.00, 129.74, 129.39, 127.35, 120.25, 119.65, 117.19, 113.54, 109.50, 107.47, 69.81, 52.66, 42.16, 36.71. ESI-MS (m/z): 405.1 $[M + H]^+$ (Chemical Formula: $C_{24}H_{24}N_2O_4$; Molecular Weight: 404.4584).

2-{2-[(3-Phenethoxyphenyl)amino]benzamido}acetic acid (25). Compound **25** was prepared following the procedure reported for **16**: **24** (0.16 g, 0.39 mmol), THF/MeOH (2 mL/1 mL), LiOH·H_2O (0.12 g, 2.77 mmol), and H_2O (1 mL); reaction time: 150 min; light yellow sticky solid (0.130 g, 0.33 mmol, 85.3%). ^{1}H-NMR (DMSO-d_6): δ = 9.58 (s, 1H), 8.87 (t, 1H, J = 5.85 Hz), 7.68 (d, 1H, J = 7.55 Hz), 7.38–7.15 (m overlap, 8H), 6.88–6.82 (m, 1H), 6.74–6.69 (m overlap, 2H), 6.55 (dd, 1H, J = 8.31, 2.08 Hz), 4.17 (t, 2H, J = 6.80 Hz), 3.90 (d, 2H, J = 5.85 Hz), 3.01 (t, 2H, J = 6.80 Hz). ^{13}C-NMR (MeOD): δ = 173.33, 171.88, 161.25, 145.84, 144.47, 139.82, 133.19, 131.01, 129.99,

129.73, 129.38, 127.33, 120.46, 119.64, 117.10, 113.50, 109.42, 107.40, 69.77, 42.07, 36.68. ESI-MS (*m*/*z*): 391.1 $[M + H]^+$; 389.0 $[M - H]^-$ (Chemical Formula: $C_{23}H_{22}N_2O_4$; Molecular Weight: 390.4318).

Benzyl {(*S*)-1-{[(*S*)-1-amino-1-oxopropan-2-yl]amino}-1-oxo-6-(2-{2-[(3-phenethoxyphenyl) amino]benzamido}acetamido)hexan-2-yl}carbamate (26). Compound **26** was prepared following the coupling procedure reported for **17**: **25** (0.07 g, 0.18 mmol), DMF (3 mL), **21** (0.07 g, 0.18 mmol), EDCI·HCl (0.052 g, 0.27 mmol), anhydrous HOBT (0.036 g, 0.27 mmol), and TEA (0.11 mL, 0.81 mmol); reaction time: 20 h; purification by column chromatography on silica Kieselgel 60 using EtOAc:MeOH (13/1) as the eluent to furnish a white solid (0.041 g, 0.057 mmol, 31.7%). R_f = 0.27 (EtOAc:MeOH = 13:1). m.p. 143–145 °C. ^{1}H-NMR (DMSO-d_6): δ = 9.56 (s, 1H), 8.70 (t, 1H, *J* = 5.85 Hz), 7.90–7.85 (m, 2H), 7.69 (d, 1H, *J* = 8.50 Hz), 7.43–7.15 (m overlap, 15H), 6.99 (s br, 1H), 6.87–6.81 (m, 1H), 6.73–6.68 (m, overlap 2H), 6.53 (dd, 1H, *J* = 8.31, 1.89 Hz), 5.02 (s, 2H), 4.22–4.14 (m overlap, 3H), 3.96–3.93 (m, 1H), 3.82 (d, 2H, *J* = 5.85 Hz), 3.04–2.99 (m overlap, 4H), 1.64–1.09 (m overlap, 9H). ^{13}C-NMR (MeOD): δ = 177.39, 174.54, 171.87, 171.76, 161.31, 158.59, 145.79, 144.75, 139.86, 138.08, 133.22, 131.05, 130.00, 129.88, 129.45, 129.39, 129.00, 128.85, 127.35, 120.95, 119.92, 117.60, 113.34, 109.40, 107.27, 69.83, 67.75, 56.52, 49.98, 43.90, 40.03, 36.71, 32.63, 29.84, 23.94, 18.19. ESI-MS (*m*/*z*): 723.4 $[M + H]^+$ (Chemical Formula: $C_{40}H_{46}N_6O_7$; Molecular Weight: 722.8292). HPLC: purity 98.3% at 254 nm, t_R: 18.67 min.

Benzyl ((*S*)-1-{[(*S*)-1-amino-1-oxopropan-2-yl]amino}-1-oxo-6-{2-[2-(phenylamino)benzamido]acetamido}hexan-2-yl)carbamate (27). Compound **27** was prepared following the coupling procedure reported for **17**: **21** (0.15 g, 0.39 mmol), DMF (4 mL), **23** (0.105 g, 0.39 mmol), EDCI·HCl (0.11 g, 0.58 mmol), anhydrous HOBT (0.078 g, 0.58 mmol), and TEA (0.24 mL, 1.7 mmol); reaction time: 18 h; purification by column chromatography on silica Kieselgel 60 using EtOAc:MeOH (12:1) as the eluent to furnish a yellow solid that was subsequently triturated with boiling EtOAc. Upon cooling at rt, the collected solid was washed with petroleum ether and

dried to afford a light yellow solid (0.12 g, 0.2 mmol, 51.3%). R*f* = 0.27 (EtOAc:MeOH = 12:1). m.p. 181–183 °C ^{1}H-NMR (DMSO-*d*$_6$): δ = 9.62 (s, 1H), 8.72 (t, 1H, *J* = 5.67 Hz), 7.95–7.86 (m overlap, 2H), 7.71 (d, 1H, *J* = 7.74 Hz), 7.44–7.28 (m overlap, 11H), 7.17–7.14 (m, 2H), 7.00–6.94 (m overlap, 2H), 6.93 (td, 1H, *J* = 7.93, 1.70 Hz), 5.02 (s, 2H), 4.25–4.15 (m, 1H), 3.98–3.92 (m, 1H), 3.83 (d, 2H, *J* = 5.67 Hz), 3.09–3.0 (m, 2H), 1.65–1.18 (m overlap, 9H). ^{13}C-NMR (DMSO-*d*$_6$): δ = 174.08, 171.42, 168.95, 168.54, 155.98, 144.24, 141.49, 136.99, 131.86, 129.32, 128.94, 128.29, 127.70, 127.58, 121.75, 119.52, 118.64, 117.97, 114.80, 65.37, 59.70, 54.66, 47.84, 42.35, 31.46, 28.73, 22.84, 18.39. ESI-MS (*m*/*z*): 603.4 $[M + H]^+$ (Chemical Formula: $C_{32}H_{38}N_6O_6$; Molecular Weight: 602.6807). HPLC: purity 99.11% at 254 nm, t_R: 16.00 min.

Benzyl ((S)-1-{[(S)-1-amino-1-oxopropan-2-yl]amino}-6-(2-benzamidoacetamido)-1-oxohexan-2-yl)carbamate (28). Compound **28** was prepared following the coupling procedure reported for **17**: **21** (0.13 g, 0.36 mmol), dry DMF (4 mL), hippuric acid (0.064 g, 0.36 mmol), EDCI·HCl (0.1 g, 0.54 mmol), anhydrous HOBT (0.073 g, 0.54 mmol), and TEA (0.21 mL, 1.5 mmol); reaction time: 16 h; purification by column chromatography on silica Kieselgel 60 using EtOAc:MeOH (8.5:1.5) as the eluent, followed by flash chromatography (DCM → DCM:MeOH, 15:1) to obtain a white solid (0.025 g, 0.047 mmol, 13%). R*f* = 0.34 (EtOAc:MeOH = 8.5:1.5) visualized with phosphomolybdic acid. m.p. 206–208 °C. ^{1}H-NMR (DMSO-*d*$_6$): δ = 8.67 (t, 1H, *J* = 5.85 Hz), 7.90–7.86 (m overlap, 4H), 7.57–7.41 (m overlap, 4H), 7.36–7.30 (m overlap, 6H), 6.99 (s br, 1H), 5.02 (s, 2H), 4.24–4.15 (m, 1H), 4.00–3.92 (m, 1H), 3.84 (d, 2H, *J* = 5.85 Hz), 3.08–3.01 (m, 2H), 1.67–1.15 (m overlap, 9H). ^{13}C-NMR (DMSO-*d*$_6$): δ = 174.07, 171.42, 168.63, 166.40, 155.99, 137.01, 134.09, 131.23, 128.31, 128.20, 127.72, 127.59, 127.32, 65.38, 54.69, 47.85, 42.62, 38.43, 31.48, 28.76, 22.85, 18.40. ESI-MS (*m*/*z*): 512.3 $[M + H]^+$, 534.3$[M + Na]^+$ (Chemical Formula: $C_{26}H_{33}N_5O_6$; Molecular Weight: 511.5701). HPLC: purity 96.97% at 254 nm, t_R: 12.32 min.

(*S*)-2-Oxo-2-phenylethyl 2-{[(benzyloxy)carbonyl]amino}-6-[(*tert*-butoxycarbonyl)amino]hexanoate (29). TEA (1.65 mL, 12 mmol) was added to a stirred solution

of Z-Lys(Boc)-OH (4.11 g, 10.8 mmol) in EtOAc (66 mL). The solution was stirred for 1 min, before 2-bromoacetophenone (2.28 g, 11.3 mmol) was added. After 16 h, the reaction was diluted with EtOAc (100 mL) and the obtained suspension washed with H_2O (50 mL). The reaction mixture was extracted with EtOAc (4 × 70 mL) and the combined organic layers were washed with brine and a saturated aqueous solution of $NaHCO_3$, dried over Na_2SO_4, and the solvent removed under reduced pressure. The solid residue was washed with Et_2O (4 × 10 mL) and dried to give a white solid (5.02 g, 10.6 mmol, 98.8%). R*f* = 0.64 (*n*-hexane:EtOAc = 1:1). ^{1}H-NMR ($CDCl_3$): δ = 7.97 (d, 2H, *J* = 7.55 Hz), 7.80 (d, 1H, *J* = 7.74 Hz), 7.70 (t, 1H, *J* = 7.55 Hz), 7.56 (t, 2H, *J* = 7.55 Hz), 7.40–7.29 (m, 5H), 6.79 (t, 1H, *J* = 5.48 Hz), 5.60 (d, 1H, *J* = 16.81 Hz), 5.48 (d, 1H, *J* = 16.81 Hz), 5.05 (s, 2H), 4.18–4.11 (m, 1H), 2.96–2.85 (m, 2H), 1.88–1.76 (m, 1H), 1.76–1.61 (m, 1H), 1.47–1.34 (m, 13H). ^{13}C-NMR (DMSO-d_6): δ = 192.41, 171.91, 156.02, 155.46, 136.78, 133.81, 133.74, 128.76(2C), 128.21, 127.65, 127.59, 77.23, 66.56, 65.40, 53.75, 30.43, 28.94, 28.13 (2C), 22.62. ESI-MS (*m*/*z*): 499.4 $[M + H]^+$ (Chemical Formula: $C_{27}H_{34}N_2O_7$; Molecular Weight: 498.5681).

(*S*)-2-Oxo-2-phenylethyl 6-amino-2-{[(benzyloxy)carbonyl]amino}hexanoate hydrochloride (30). HCl in dioxane (4 N, 16.5 mL) was slowly added to a stirred solution of **29** (4.90 g, 10.4 mmol) in dry CH_2Cl_2 (80 mL), which was cooled in an ice bed. The reaction was then warmed to room temperature, where stirring was continued for 280 min. Thereafter, the solvents were evaporated and the residue washed twice with $CHCl_3$:Et_2O 1:1 (4 × 40 mL) to give a white solid (3.82 g, 8.78 mmol, 82.8%). ^{1}H-NMR (DMSO-d_6): δ = 7.98–7.82 (m overlap, 6H), 7.67 (t, 1H, *J* = 7.37 Hz), 7.55 (t, 1H, *J* = 7.37 Hz), 7.36, 7.31 (m, 5H), 5.60 (d, 1H, *J* = 17.0 Hz), 5.47 (d, 1H, *J* = 17.0 Hz), 5.03 (s, 2H), 4.19–4.12 (m, 1H), 2.78–2.73 (m, 2H), 1.91–1.65 (m overlap, 2H), 1.61–1.42 (m overlap, 4H). ^{13}C-NMR (DMSO-d_6): δ = 192.58, 171.95, 156.16, 136.86, 133.97, 133.81, 128.90 (2C), 128.35, 127.79, 127.71, 66.77, 65.55, 53.75, 38.37, 30.26, 26.41, 22.39. ESI-MS (*m*/*z*): 399.2 $[M + H]^+$ (Chemical Formula: $C_{22}H_{26}N_2O_5$; Molecular Weight: 398.4522).

(*S*)-2-Oxo-2-phenylethyl 2-{[(benzyloxy)carbonyl]amino}-6-{2-[(*tert*-

butoxycarbonyl)amino]acetamido}hexanoate (31). Boc-Gly-OH (1.50 g, 8.5 mmol), EDCI·HCl (2.44 g, 12.75 mmol), anhydrous HOBT (1.72 g, 12.75 mmol), and TEA (5.3 mL, 38 mmol) were added to a solution of **30** (3.72 g, 8.5 mmol) in dry DMF (30 mL), which was cooled in an ice bed. The reaction was stirred under an atmosphere of N_2 for 25 h. Thereafter, brine (50 mL) was added and the reaction mixture extracted with EtOAc (5 × 70 mL). The combined organic phases were washed with a saturated aqueous solution of $NaHCO_3$ (40 mL) and brine (40 mL), before they were dried over Na_2SO_4 and the solvent was evaporated under reduced pressure. The residue was purified by flash chromatography on silica (EtOAc:*n*-hexane, 1:1 → EtOAc) to furnish a white solid (3.87 g, 6.9 mmol, 76.7%). R*f* = 0.61 EtOAc. ^{1}H-NMR (DMSO-*d*$_6$): δ = 7.96 (d, 2H, *J* = 7.18 Hz), 7.80–7.67 (m overlap, 3H), 7.56 (t, 2H, *J* = 7.37 Hz), 7.38–7.31 (m overlap, 5H), 6.86 (t, 1H, *J* = 5.85 Hz), 5.60 (d, 1H, *J* = 17.0 Hz), 5.47 (d, 1H, *J* = 17.0 Hz), 5.02 (s, 2H), 4.19–4.12 (m, 1H), 3.50 (d, 2H, *J* = 6.04 Hz), 3.12–3.01 (m, 2H), 1.91–1.80 (m, 1H), 1.76–1.59 (m, 1H), 1.48–1.38 (m overlap, 13H). ^{13}C-NMR (DMSO-*d*$_6$): δ = 192.53, 172.04, 169.00, 156.12, 155.71, 136.88, 133.94, 133.81, 128.88, 128.32, 127.77 (3C), 77.94, 66.69, 65.50, 53.80, 43.23, 38.19, 30.53, 28.65, 28.15, 22.81. ESI-MS (*m*/*z*): 556.4 [M + H]$^+$ (Chemical Formula: $C_{29}H_{37}N_3O_8$; Molecular Weight: 555.6194).

(*S*)-2-Oxo-2-phenylethyl 2-{[(benzyloxy)carbonyl]amino}-6-{2-[(*tert*-butoxycarbonyl)amino]ethanethioamido}hexanoate (32). **31** (2.0 g, 3.6 mmol) and Lawesson's reagent (0.72 g, 1.8 mmol) were added to a dried reaction tube under a flow of argon, followed by dry toluene (40 mL). The suspension was warmed to 60 °C and stirred for 6 h under an atmosphere of argon. Thereafter, all volatiles were evaporated under reduced pressure and the residue directly purified by flash chromatography on silica (*n*-hexane → *n*-hexane:EtOAc, 1:1) to provide a colorless sticky solid (2.06 g, 3.6 mmol, 100%). R*f* = 0.37 (EtOAc:*n*-hexane = 1:1). ^{1}H-NMR (DMSO-*d*$_6$): δ = 9.73 (pseudo s, 1H), 7.97 (d, 2H, *J* = 7.18 Hz), 7.81 (d, 1H, *J* = 7.74 Hz), 7.73–7.67 (m, 1H), 7.57 (t, 2H, *J* = 7.36 Hz), 7.41–7.30 (m overlap, 5H), 7.04 (t, 1H, *J* = 5.67 Hz), 5.60 (d, 1H, *J* = 17.0 Hz), 5.47 (d, 1H, *J* = 17.0 Hz), 5.05 (s, 2H), 4.21–4.13 (m, 1H), 3.89 (d, 2H, *J* = 5.85 Hz), 3.60–3.50 (m,

2H), 1.94–1.81 (m, 1H), 1.79–1.69 (m, 1H), 1.65–1.52 (m, 2H), 1.46–1.39 (m overlap, 11H). ^{13}C-NMR (DMSO-*d*$_6$): δ = 199.17, 192.52, 172.01, 156.12, 155.54, 136.87, 133.94, 133.82, 128.87, 128.32, 127.77 (3C), 78.33, 66.70, 65.50, 53.78, 50.91, 44.54, 30.54, 28.11, 26.67, 22.87. ESI-MS (*m*/*z*): 572.3 [M + H]$^+$, 594.3 [M + Na]$^+$ (Chemical Formula: $C_{29}H_{37}N_3O_7S$; Molecular Weight: 571.6850).

(*S*)-2-{[(Benzyloxy)carbonyl]amino}-6-{2-[(*tert*-butoxycarbonyl)amino]ethanethioamido}hexanoic acid (33). Compound **33** was prepared following the procedure reported for **16**: **32** (2.0 g, 3.5 mmol), THF:MeOH (25 mL/12.5 mL), and a solution of LiOH (1.02 g, 24 mmol) in H_2O (12.5 mL); reaction time: 4 h; purification by column chromatography on silica Kieselgel 60 using DCM:MeOH:AcOH (20:1:0.5) as the eluent to furnish a yellow sticky solid (1.06 g, 2.3 mmol, 66.8%). R*f* = 0.27 (DCM:MeOH:AcOH = 20:1:0.5).^{1}H-NMR (DMSO-*d*$_6$): δ = 12.5 (s br, 1H), 9.68 (s br, 1H), 7.53 (d, 1H, *J* = 7.93 Hz), 7.43– 7.13 (m overlap, 6H), 7.03–6.98 (m, 1H), 5.02 (s, 2H), 3.95–3.86 (m overlap, 3H), 3.59–3.45 (m, 2H), 1.74–1.49 (m overlap, 4H), 1.41–1.29 (m overlap, 10H). ^{13}C-NMR (DMSO-*d*$_6$): δ = 199.20, 173.88, 156.17, 155.56, 137.02, 128.31, 127.77, 127.68, 78.38, 65.39, 53.78, 50.91, 44.58, 30.46, 28.13, 26.71, 23.03. ESI-MS (*m/z*): 452.0 [M - H]$^-$; 454.3 [M + H]$^+$ (Chemical Formula: $C_{21}H_{31}N_3O_6S$; Molecular Weight: 453.5523).

Benzyl ((*S*)-1-{[(*S*)-1-amino-1-oxopropan-2-yl]amino}-6-[2-(tert-butoxycarbonylamino)ethanethioamido]-1-oxoexan-2-yl)carbamate (34). COMU (0.56 g, 1.32 mmol) and TEA (0.92 mL, 6.6 mmol) were sequentially added to a solution of **33** (0.5 g, 1.1 mmol) in dry DMF (5 mL), which was cooled in an ice bed. The reaction mixture was stirred under a N_2 flow for 1 min, before L-alaninamide hydrochloride (0.15 g, 1.21 mmol) was added, and stirring at room temperature was continued for 22 h. Thereafter, brine (30 mL) was added, and the reaction mixture was extracted with EtOAc (4 × 50 mL) and dried over Na_2SO_4, before all volatiles were removed under reduced pressure. The residue was purified by column chromatography on silica

Kieselgel 60 using DCM:MeOH (20:1) as the eluent to furnish a light yellow solid (0.37 g, 0.7 mmol, 64.2%). R*f* = 0.20 (DCM:MeOH = 20:1). ^{1}H-NMR (DMSO-*d*$_6$): δ = 9.69 (s br, 1H), 7.86 (d, 1H, *J* = 7.55 Hz), 7.44–7.31 (m, overlap, 7H), 7.04–7.00 (m , overlap, 2H), 5.02 (s, 2H), 4.22–4.15 (m, 1H), 4.02–3.95 (m, 1H), 3.87 (d, 2H, *J* = 5.85 Hz), 3.55–3.43 (m, 2H), 1.70–1.45 (m overlap, 4H), 1.44–1.27 (m overlap, 11H), 1.20 (d, 3H, *J* = 6.99 Hz). ^{13}C-NMR (DMSO-*d*$_6$): δ = 199.13, 174.06, 171.36, 155.97, 155.52, 136.98, 128.29, 127.70, 127.58, 78.33, 65.37, 54.62, 50.86, 47.83, 44.71, 31.44, 28.11, 26.77, 22.87, 18.40. ESI-MS (*m/z*): 524.3 [M + H]$^+$ (Chemical Formula: $C_{24}H_{37}N_5O_6S$; Molecular Weight: 523.6455).

Benzyl ((*S*)-1-{[(*S*)-1-amino-1-oxopropan-2-yl]amino}-6-(2-aminoethanethioamido)-1-oxohexan-2-yl)carbamate hydrochloride (35). Compound **35** was prepared following the cleavage procedure described for **13**: **34** (0.25 g, 0.47 mmol), 2-propanol (8.7 mL), triisopropylsilane (0.13 mL, 0.62 mmol), and a 37% aqueous solution of HCl (3 × 0.26 mL). A light yellow solid was obtained (0.174 g, 0.38 mmol, 79.3%). ^{1}H-NMR (DMSO-*d*$_6$): δ = 10.54 (s br, 1H), 8.21 (s br, 3H), 7.91 (d, 1H, *J* = 7.55 Hz), 7.47–7.31 (m, overlap, 7H), 7.01 (s, 1H), 5.02 (s, 2H), 4.25–4.15 (m, 1H), 4.02–3.94 (m, 1H), 3.78 (s, 2H), 3.53–3.48 (t, 2H, *J* = 6.99 Hz), 1.70–1.47 (m overlap, 4H), 1.42–1.30 (m, 2H), 1.21 (d, 3H, *J* = 6.99 Hz). ^{13}C-NMR (DMSO-*d*$_6$): δ = 193.74, 174.15, 171.32, 155.95, 136.99, 128.29, 127.70, 127.57, 65.36, 54.58, 47.93, 45.95, 45.19, 31.39, 26.51, 22.89, 18.39. ESI-MS (*m/z*): 424.3 [M + H]$^+$ (Chemical Formula: $C_{19}H_{29}N_5O_4S$; Molecular Weight: 423.5297).

Benzyl [(*S*)-1-{[(*S*)-1-amino-1-oxopropan-2-yl]amino}-1-oxo-6-(2-{2-[(3-phenethoxyphenyl)amino]benzamido}ethanethioamido)hexan-2-yl]carbamate (36). Compound **36** was prepared following the coupling procedure reported for **17**: **16** (0.08 g, 0.24 mmol), dry DMF (4 mL), **35** (0.11 g, 0.24 mmol), EDCI·HCl (0.069 g, 0.36 mmol), anhydrous HOBT (0.049 g, 0.36 mmol), and TEA (0.15 mL, 1.08 mmol); reaction time: 330 min; purification by column chromatography on silica Kieselgel 60 with DCM:MeOH (30:1) as the eluent to furnish a light yellow solid (0.08 g, 0.11 mmol, 45.1%). R*f* = 0.18 (DCM:MeOH = 30:1). m.p. 126–128 °C. ^{1}H-NMR

(DMSO-d_6): δ = 9.88 (s br, 1H), 9.54 (s, 1H), 8.84 (t, 1H, J = 4.72 Hz), 7.87 (d, 1H, J = 7.37 Hz), 7.76 (d, 1H, J = 7.55 Hz), 7.44–7.15 (m overlap, 15H), 6.99 (s br, 1H), 6.89–6.83 (m, 1H), 6.73–6.68 (m overlap, 2H), 6.54 (dd, 1H, J = 8.12, 1.70 Hz), 5.02 (s, 2H), 4.25–4.15 (m overlap, 5H), 4.00–3.93 (m, 1H), 3.56–3.47 (m, 2H), 3.01 (t, 2H, J = 6.80 Hz), 1.70–1.46 (m overlap, 4H), 1.41–1.29 (m, 2H), 1.20 (d, 3H, J = 7.18 Hz). ^{13}C-NMR (DMSO-d_6): δ = 198.72, 174.05, 171.34, 168,79, 159.42, 155.98, 144.08, 142.87, 138.37, 137.00, 131.98, 130.08, 128.89 (2C), 128.29, 128.25, 127.70, 127.58, 126.20, 118.87, 118.25, 115.51, 111.70, 108.02, 105.47, 68.03, 65.38, 54.62, 49.52, 47.84, 44.96, 38.72, 34.91, 26.76, 22.94, 18.41. ESI-MS (*m/z*): 739.6 [M + H]$^+$ (Chemical Formula: $C_{40}H_{46}N_6O_6S$; Molecular Weight: 738.8948). HPLC: purity 98.28% at 254 nm, t_R: 20.75 min.

General procedure for the synthesis of 37 and 38. Example of *tert*-Butyl (2-(propylamino)-2-thioxoethyl)carbamate (37). **11** (0.5 g, 2.3 mmol) was dissolved in dry toluene under argon flow, then Lawesson's reagent (0.46 g, 1.15 mmol) was added. The reaction was left under stirring under argon atmosphere at 60ºC for 3h, 30min. After this time solvent was removed under reduced pressure and the residue purified by flash chromatography starting from *n*-Hexane: EtOAc (8:2) followed by *n*-Hexane: EtOAc (2:1) to provide a white solid (0.538 g, 2.3 mmol, Yield 100%). R*f* = 0.7 *n*-Hexane: EtOAc (1:1) visualized with Phosphomolybdic acid. ^{1}H-NMR: (DMSO-d$_6$): δ = 9.66 (s br, 1H), 7.04-6.99 (m, 1H), 3.88 (d, 2H, J = 6.04 Hz), 3.53-3.46 (m, 2H), 1.63-1.51 (m, 2H), 1.39 (s, 9H), 0.86 (t, 3H, J = 7.37 Hz). ^{13}C-NMR (DMSO-d_6): δ = 199.23, 155.52, 78.31, 50.92, 46.46, 28.09, 20.49, 11.25. ESI-MS (*m/z*): 233.2 [M + H]$^+$ (Chemical Formula: $C_{10}H_{20}N_2O_2S$; Molecular Weight: 232.3430).

***tert*-Butyl (2-(pentylamino)-2-thioxoethyl)carbamate (38)**. White solid (0.472 g, 1.8 mmol, 78.8%). R*f* = 0.8 *n*-Hexane: EtOAc (1:1) visualized with Phosphomolybdic acid. ^{1}H-NMR: (DMSO-d$_6$): δ = 9.63 (s br, 1H), 7.03-6.99 (m, 1H), 3.85 (d, 2H, J = 5.85 Hz), 3.56-3.49 (m, 2H), 1.60-1.50 (m, 2H), 1.39-1.24 (m overlap, 13H), 0.87 (t, 3H, J = 6.99 Hz). ^{13}C-NMR (DMSO-d_6): δ = 199.05, 155.49, 78.28, 50.91, 44.68, 28.44, 28.06, 26.77, 21.76, 13.74. ESI-MS (*m/z*): 261.3 [M + H]$^+$ (Chemical Formula: $C_{12}H_{24}N_2O_2S$; Molecular Weight: 260.3962).

General procedure for the synthesis of 39 and 40. Example of 2-Amino-N-propylethanethioamide hydrochloride (39). To a solution of **37** (0.21 g, 0.92 mmol). in 2-propanol (7 mL), triisopropylsilane (0.25 mL, 1.2 mmol) was added, followed by HCl 37% (0.21 mL) under stirring, then warmed at 50ºC. After 20 min and 40 min a portion of HCl 37% was added (each portion 0.21 mL). When the total amount of HCl in the reaction flask was 0.63 mL, the solution was maintained for 1h at 50°C under stirring. After this time 2-propanol was removed under reduced pressure and the sticky residue washed with Et_2O (3x5mL), EtOAc (2x 5 mL), and Et_2O (3x 5 mL) providing the product as light white solid (0.12 g, 0.71 mmol, 77.2%). ^{1}H-NMR: (DMSO-d^6): δ = 10.84 (s br, 1H), 8.33 (s br, 3H), 3.81 (s, 2H), 3.48 (t, 2H, J = 7.18 Hz), 1.67-1.55 (m, 2H), 0.91 (t, 3H, J = 7.55 Hz). ^{13}C-NMR (DMSO-d_6): δ = 193.80, 47.16, 45.92, 20.36, 11.53. ESI-MS (*m/z*): 133.2 $[M + H]^+$ (Chemical Formula: $C_5H_{12}N_2S$; Molecular Weight: 132.2272).

2-Amino-N-pentylethanethioamide hydrochloride (40). From **38** (0.24 g, 0.92 mmol). White solid (0.08 g, 0.41 mmol, 44.2%). ^{1}H-NMR: (DMSO-d^6): 10.64 (s br, 1H), 8.30 (s br, 3H), 3.80 (s br, 2H), 3.52 (t, 2H, J = 7.18 Hz), 1.64-1.55 (m, 2H), 1.33-1.27 (m overlap 4H), 0.88 (t, 3H, J = 7.18 Hz). ^{13}C-NMR (DMSO-d_6): δ = 194.82, 46.99, 46.80, 30.21, 28.22, 23.33, 14.23. ESI-MS (*m/z*): 161.2 $[M + H]^+$ (Chemical Formula: $C^7H_{16}N_2S$; Molecular Weight: 160.2803).

2-((3-Phenethoxyphenyl)amino)-N-(2-(propylamino)-2-thioxoethyl)benzamide (41). **16** (0.08 g, 0.24 mmol), **39** (0.040g, 0.24 mmol), EDCI·HCl (0.069 g, 0.36 mmol), anhydrous HOBT (0.048 g, 0.36 mmol), and TEA (0.15 mL, 1.08 mmol). Purification by column chromatography, using silica Kieselgel 60 with EtOAc: *n*-hexane (2.3:7.7) as eluent phase to furnish a white solid (0.081g, 0.182 mmol, 75.8%). R*f* = 0.74 (EtOAc: *n*-hexane 1:1). m.p. 123-125 ºC. ^{1}H-NMR (DMSO-d_6): δ = 9.87 (s br, 1H), 9.52 (s br, 1H), 8.89 (s br, 1H), 7.76 (d, 1H, J = 8.69 Hz), 7.39-7.15 (m overlap, 8H), 6.89-6.84 (m, 1H), 6.73-6.67 (m overlap 2H), 6.55 (dd, 1H, J = 7.55, 1.70 Hz), 4.19-4.14 (m overlap, 4H), 3.50 (t, 2H, J = 7.18 Hz), 3.02 (t, 2H, J = 6.80 Hz), 1.64-1.51 (m, 2H), 0.85 (t, 3H, J = 7.37 Hz). ^{13}C-NMR (DMSO-d_6): δ = 198.76, 168.84, 159.40, 144.05, 142.86, 138.34, 131.96, 130.05, 129.17,

128.87, 128.22, 126.18, 118.88, 118.22, 115.51, 111.61, 107.96, 105.41, 68.00, 49.67, 46.64, 34.89, 20.48, 11.29. ESI-MS (*m*/*z*): 448.5 $[M + H]^+$. HPLC: purity 99.36% at 254nm, t_R: 22.98 min. HRMS (EI) calcd for $C_{26}H_{29}N_3O_2S$, 447.19805; found, 447.19616.

***N*-(2-(Pentylamino)-2-thioxoethyl)-2-((3-phenethoxyphenyl)amino)benzamide (42)**. **16** (0.08 g, 0.24 mmol), **40** (0.047 g, 0.24 mmol), EDCI·HCl (0.069 g, 0.36 mmol), anhydrous HOBT (0.048 g, 0.36 mmol), and TEA (0.15mL, 1.08 mmol). Purification by column chromatography on silica Kieselgel 60 with EtOAc: *n*-hexane (2:8) as eluent phase to furnish a white solid (0.084 g, 0.177 mmol, 73.8%). R*f* = 0.85 (EtOAc: *n*-hexane 1:1). m.p. 102-104 ºC. ^{1}H-NMR (DMSO-d_6): δ = 9.85 (s br, 1H), 9.54 (s br, 1H), 8.89 (s br, 1H), 7.76 (d, 1H, *J* = 6.99 Hz), 7.39-7.15 (m overlap, 8H), 6.89-6.84 (m, 1H), 6.73-6.67 (m overlap, 2H), 6.55 (dd, 1H, *J* = 7.74, 1.89 Hz), 4.19-4.15 (m overlap, 4H), 3.53 (t, 2H, *J* = 7.37 Hz), 3.02 (t, 2H, *J* = 6.80 Hz), 1.62-1.51 (m, 2H), 1.32-1.21 (m overlap, 4H), 0.83 (t, 3H, *J* = 6.80 Hz). ^{13}C-NMR (DMSO-d_6): δ = 198.62, 168.88, 159.42, 144.11, 142.87, 138.36, 131.99, 130.07, 129.18, 128.88, 128.24, 126.20, 118.85, 118.22, 115.49, 111.66, 107.99, 105.50, 68.03, 49.72, 44.93, 34.92, 28.51, 26.80, 21.77, 13.77. ESI-MS (*m*/*z*): 476.5 $[M + H]^+$. HPLC: purity 98.85% at 254nm, t_R: 24.38 min. HRMS (EI) calcd for $C_{28}H_{33}N_3O_2S$, 475.22935; found, 475.22871.

HPLC chromatograms for 17–19, 26–28, 36, 41 and 42.

HPLC chromatogram for **17**. Purity 98.88%; t_R: 18.33 min.

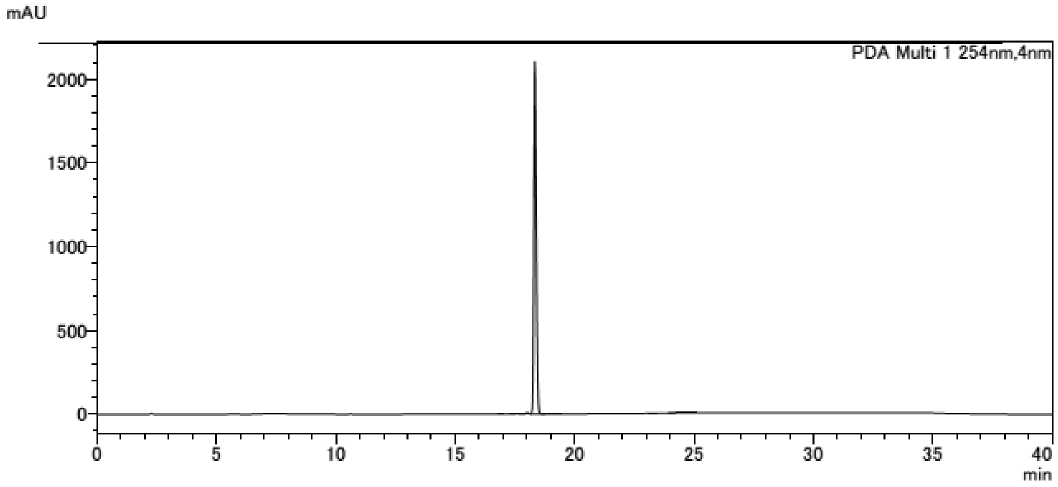

HPLC chromatogram for **18**. Purity 98.94%; t_R: 20.63min.

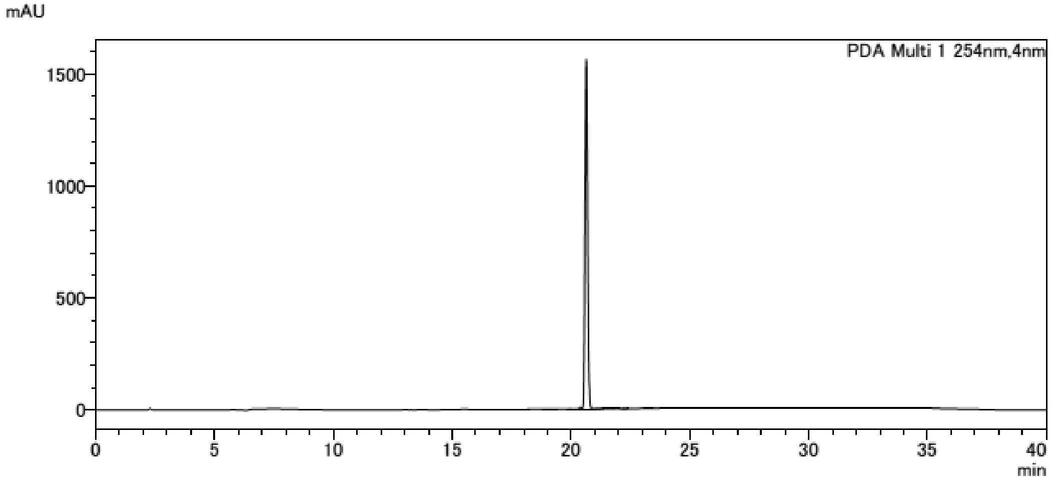

HPLC chromatogram for **19**. Purity 99.35%; t_R: 22.27 min.

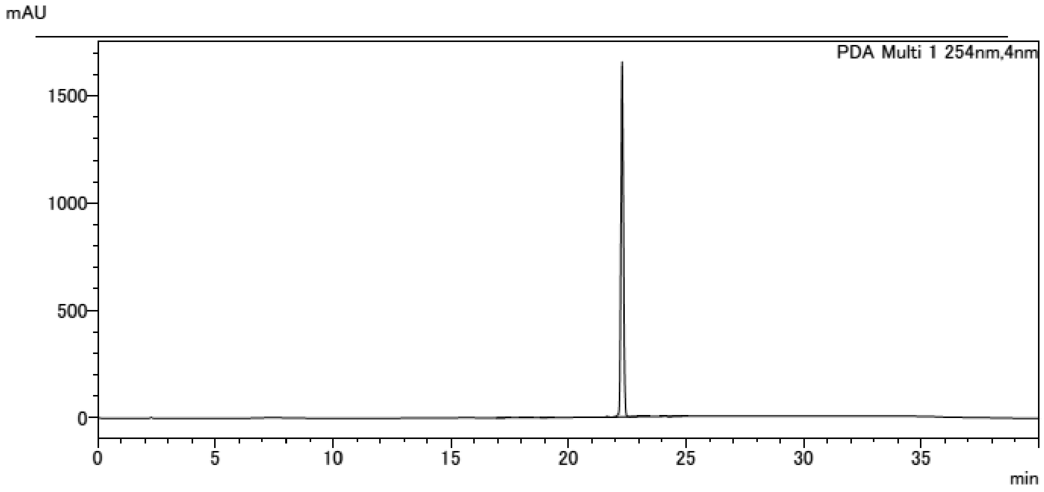

HPLC chromatogram for **26**. Purity 98.3%; t_R: 18.67 min.

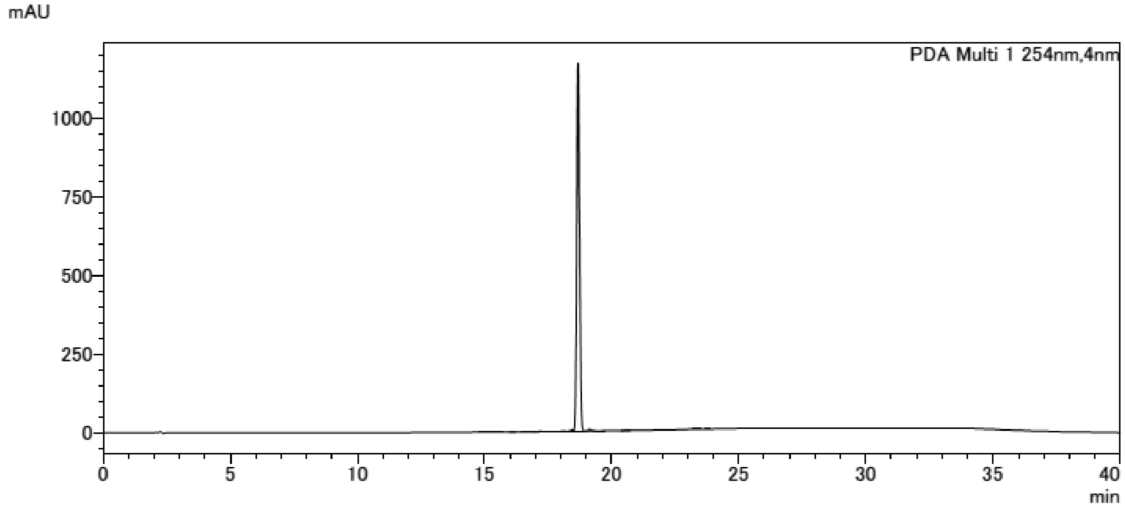

HPLC chromatogram for **27**. Purity 99.11%; t_R: 16.00 min.

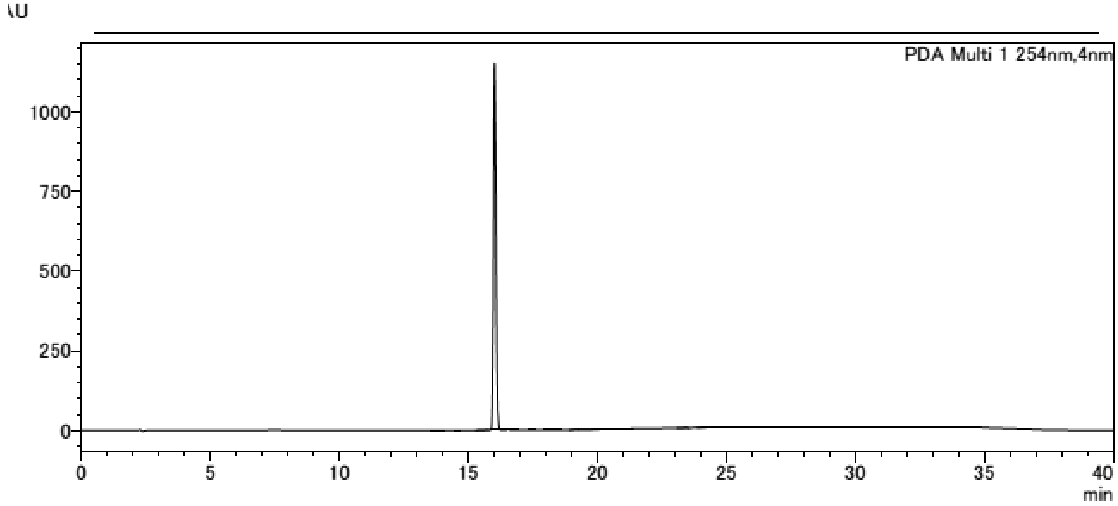

HPLC chromatogram for **28**. Purity 96.97%; t_R: 12.32 min.

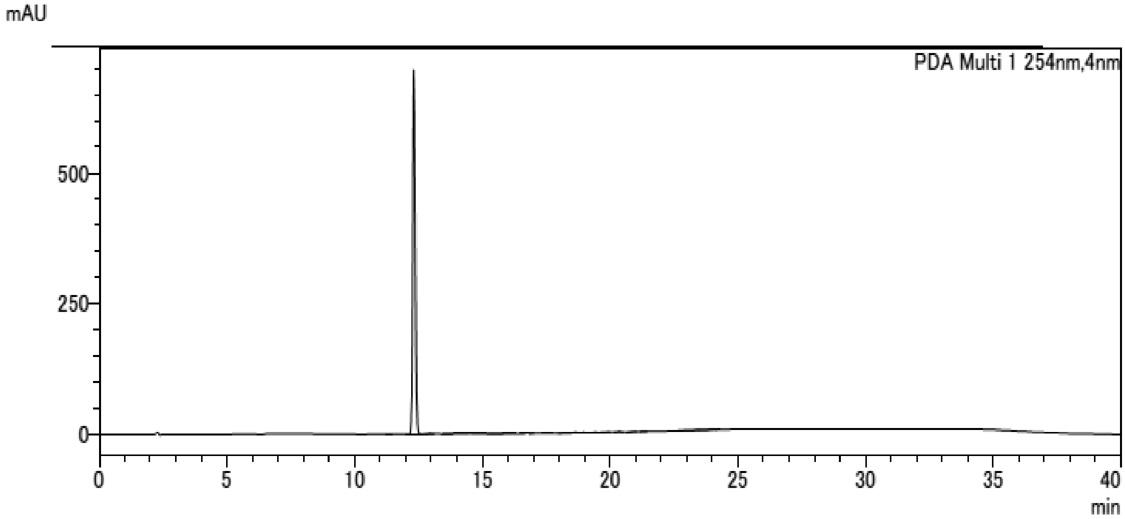

HPLC chromatogram for **36**. Purity 98.28%; t_R: 20.75 min.

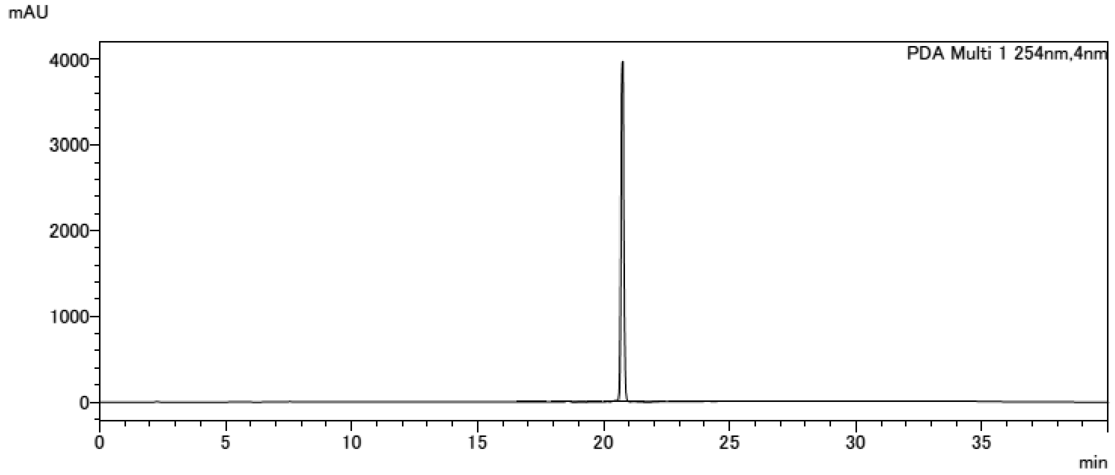

HPLC chromatogram for **41**. Purity 99.36%, $t_{R:}$ 22.98 min

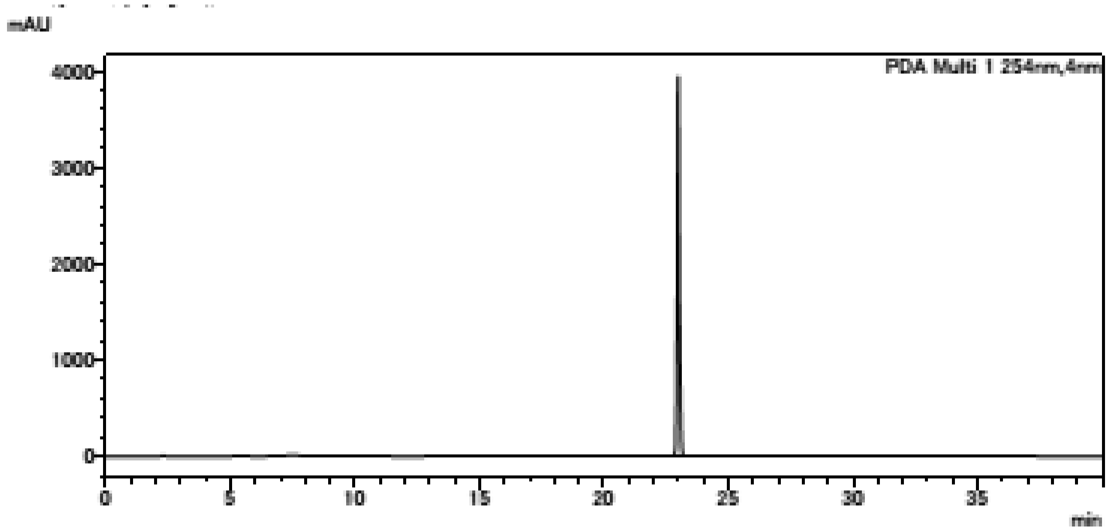

HPLC chromatogram for **42**. Purity 98.85%, $t_{R:}$ 24.38 min

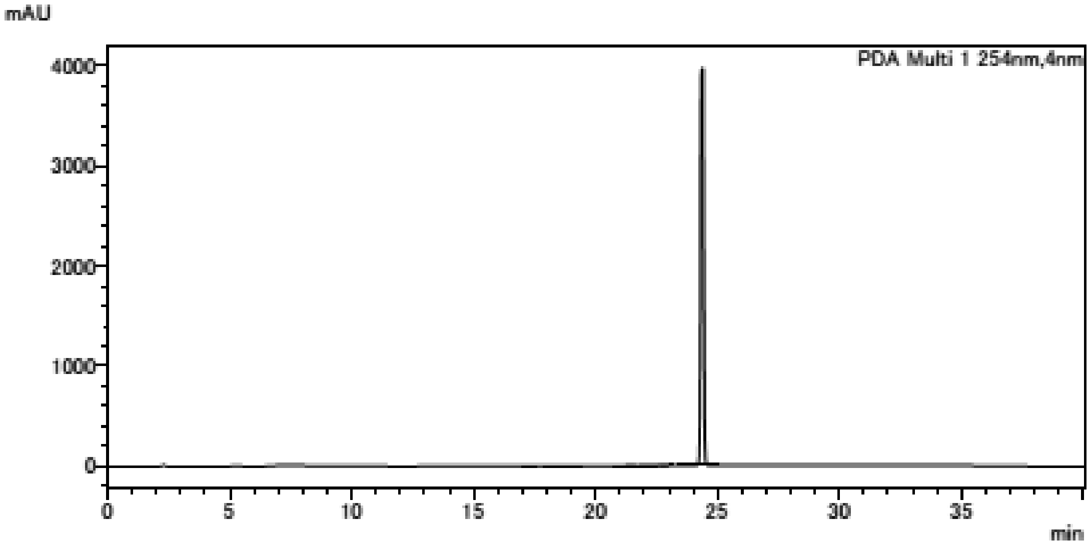

^{1}H and ^{13}C-NMR spectra for 17–19, 26–28, 36, 41 and 42.

^{1}H NMR spectrum for **17** in DMSO-d_6 at 299.7 K

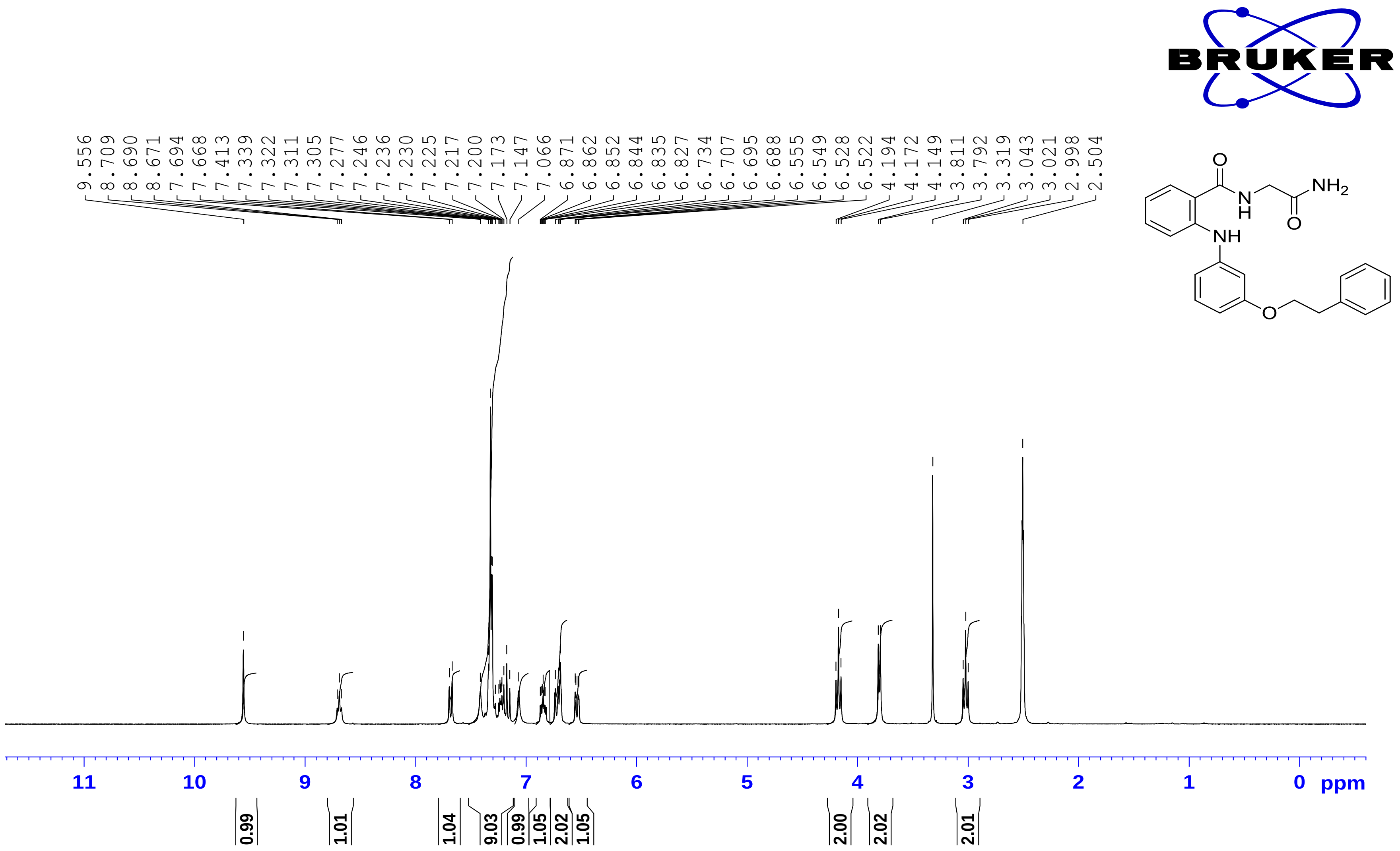

^{13}C NMR spectrum for **17** in MeOD + 6% DMSO-d_6 at 301.2 K

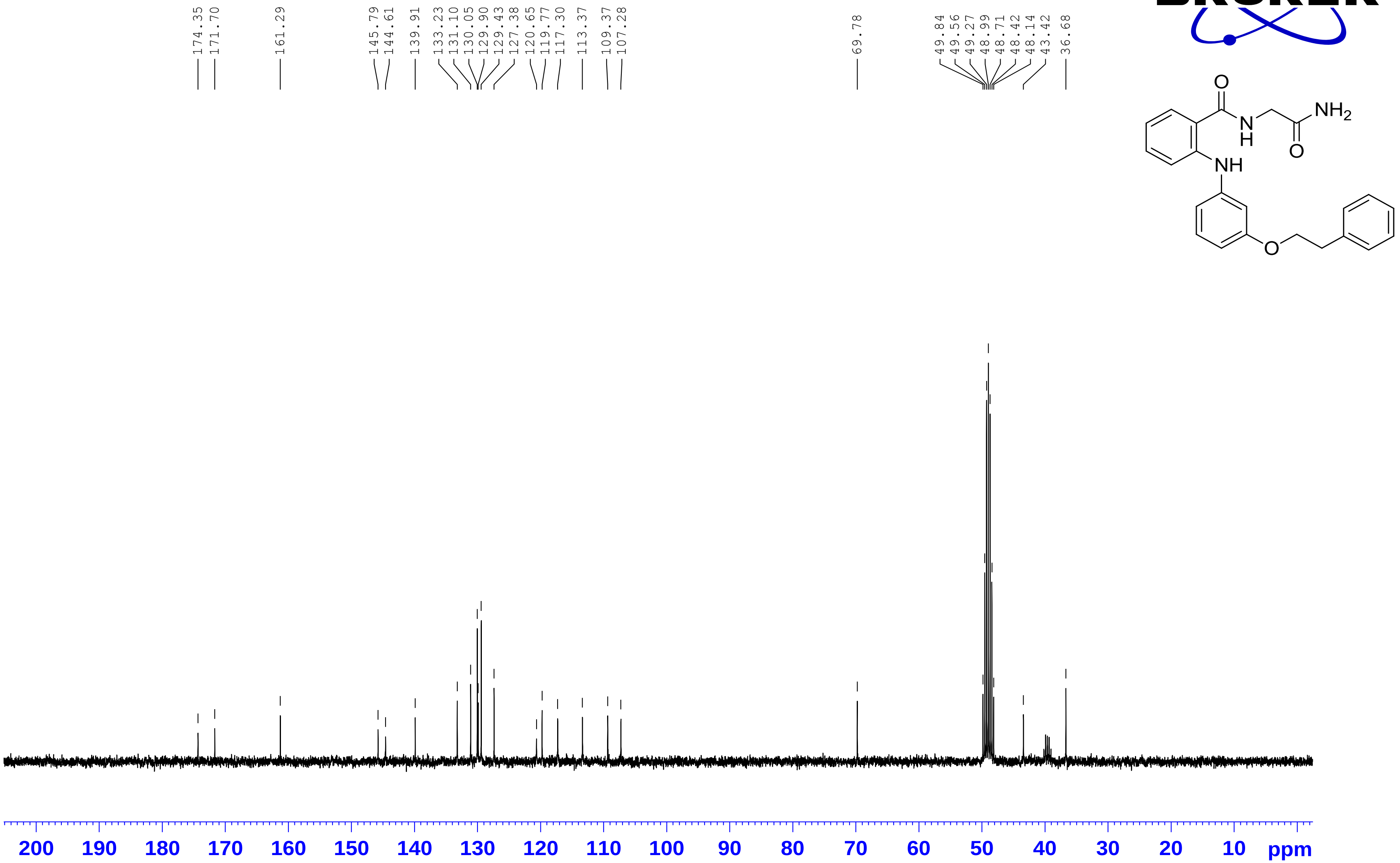

^{1}H NMR spectrum for **18** in DMSO-d_6 at 299.6 K

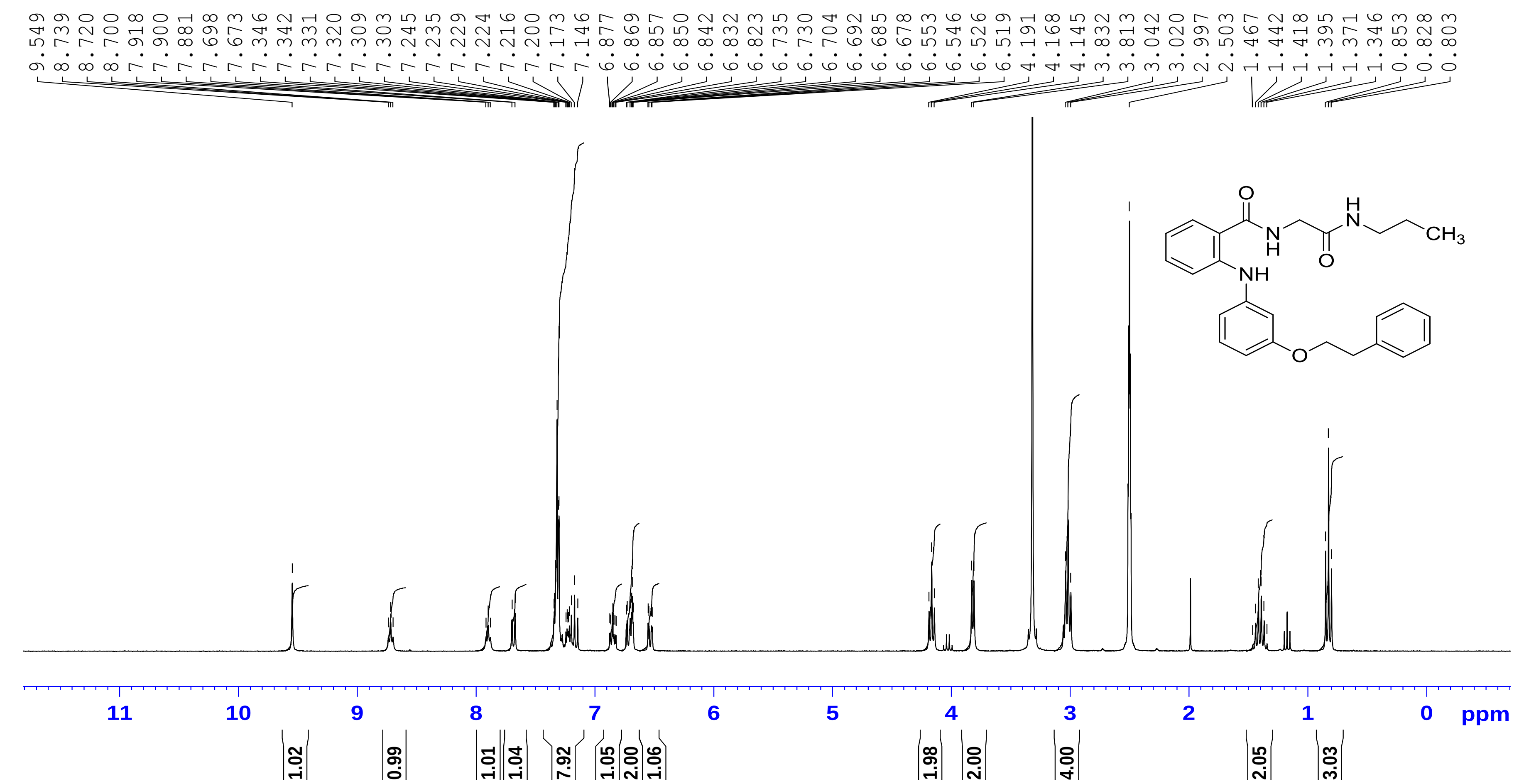

^{13}C NMR spectrum for **18** in MeOD at 301.7 K

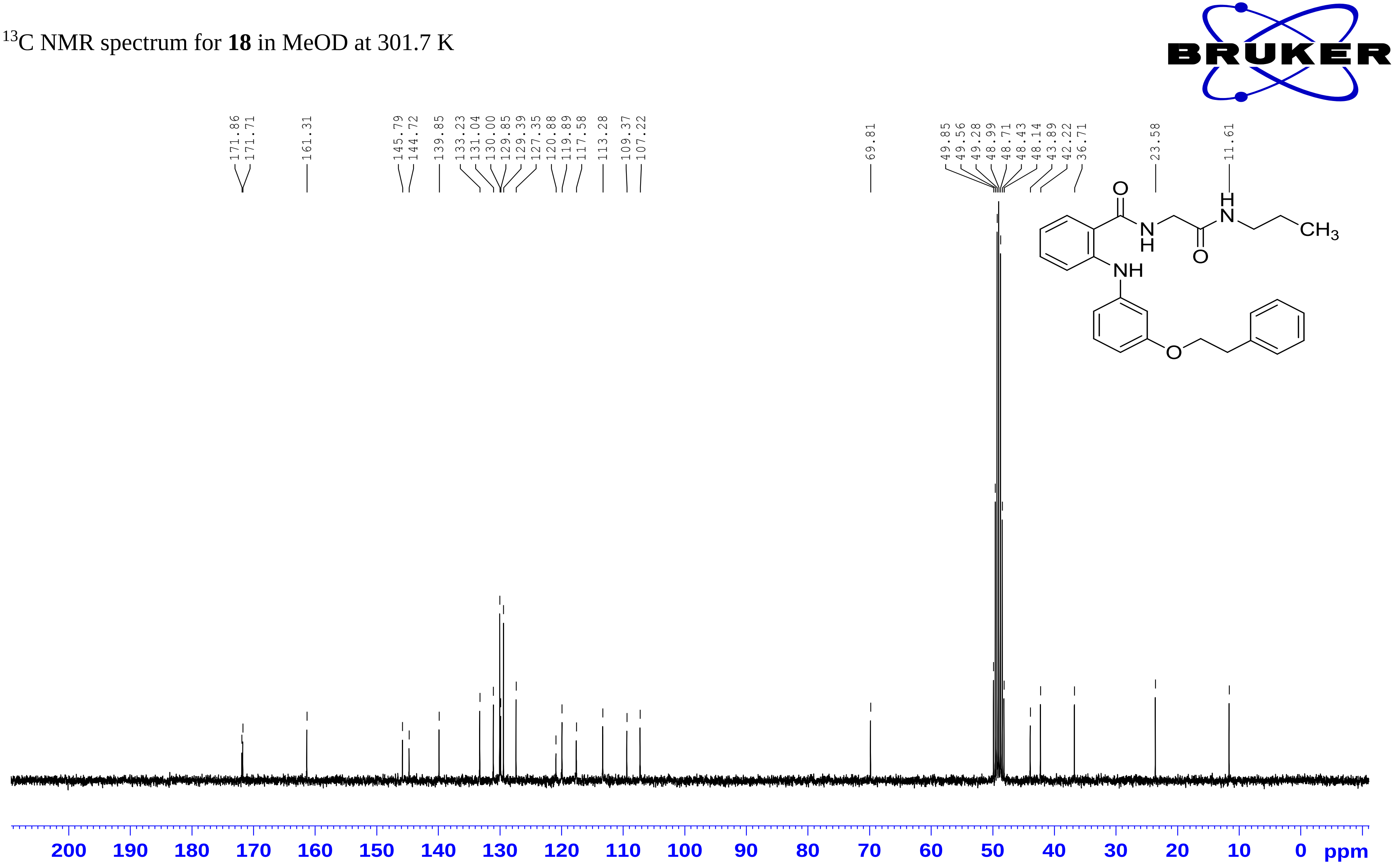

^{1}H NMR spectrum for **19** in DMSO-d_6 at 299.6 K

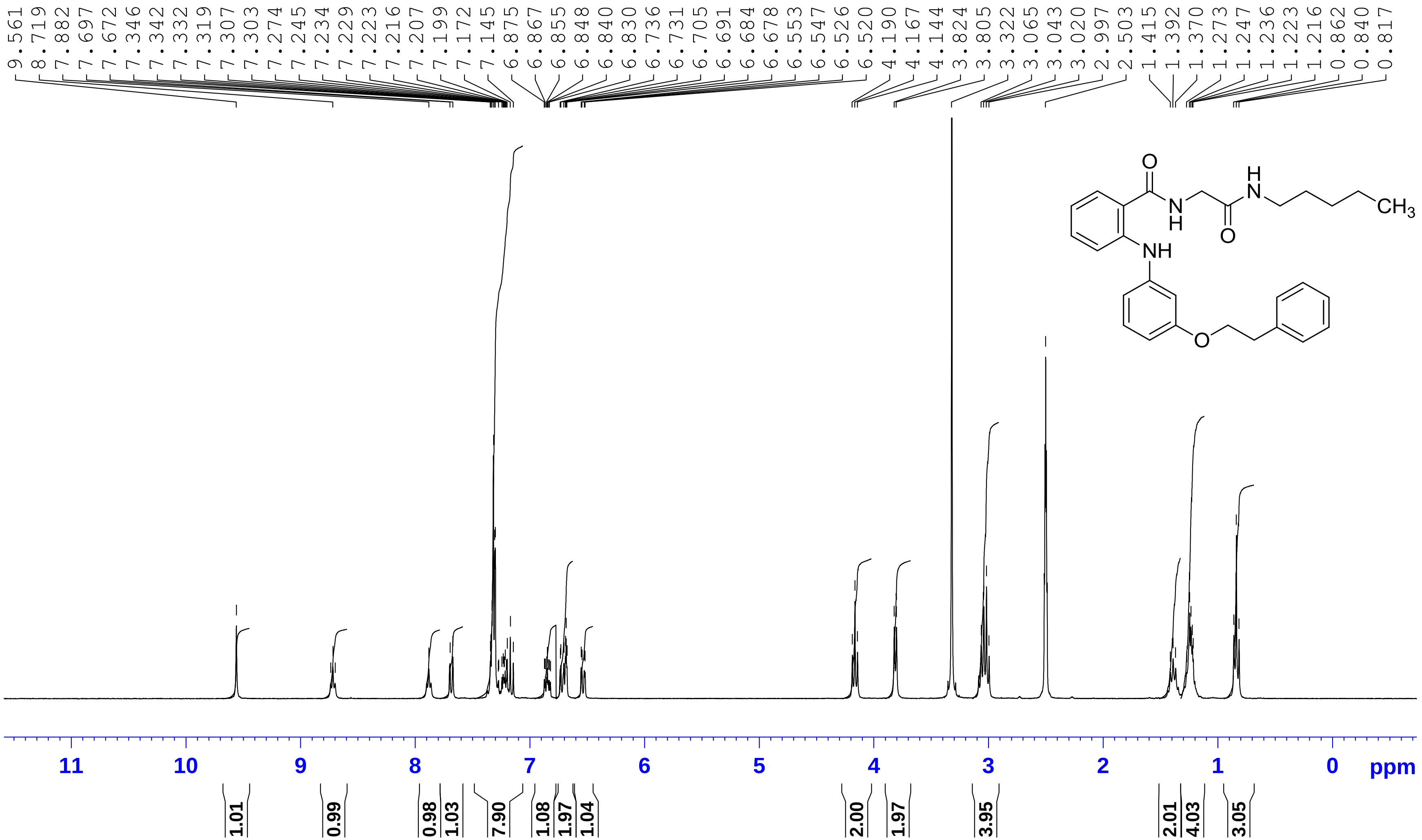

^{13}C NMR spectrum for **19** in MeOD at 302.8 K

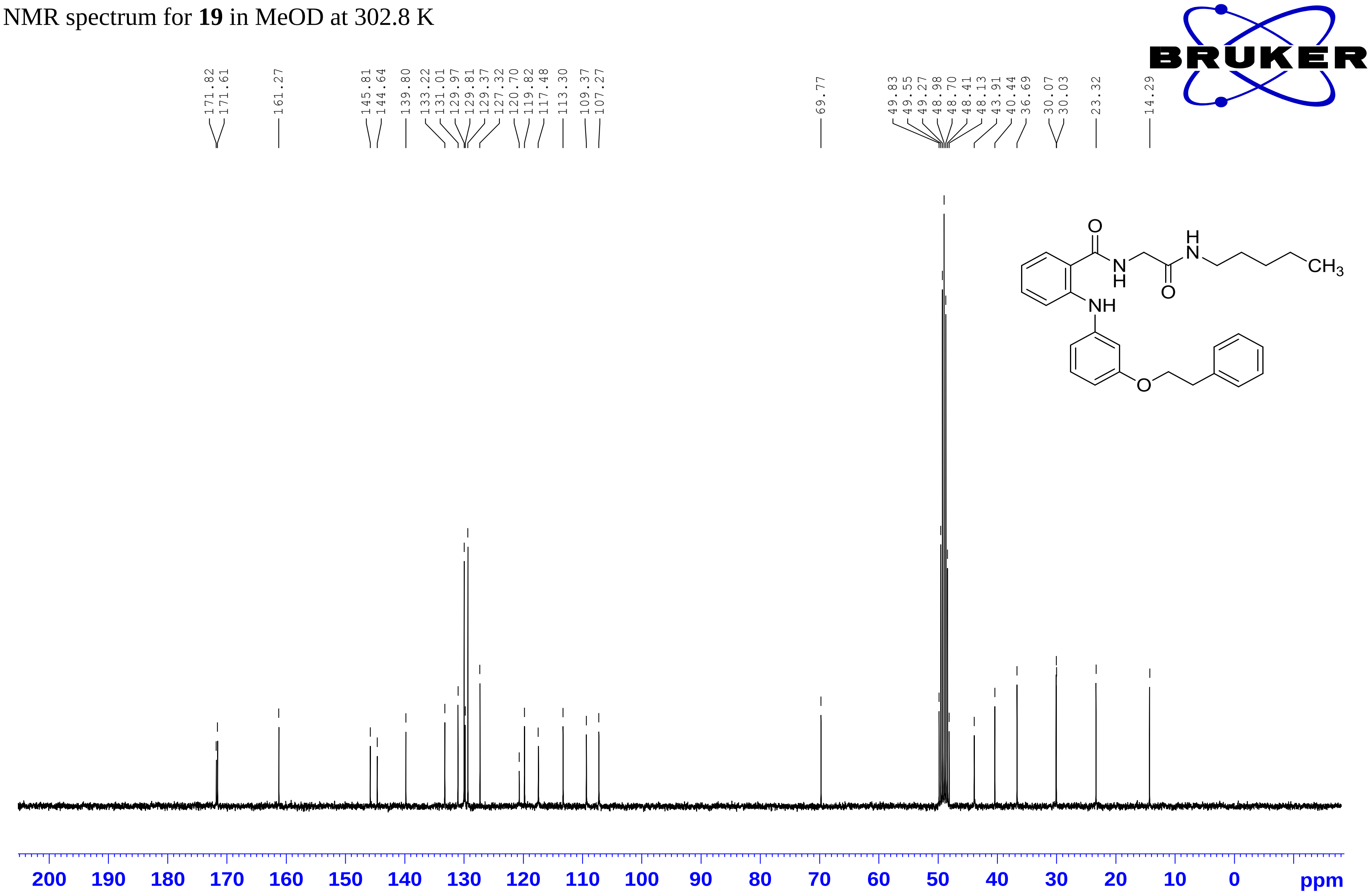

^{1}H NMR spectrum for **26** in DMSO-d_6 at 299.9 K

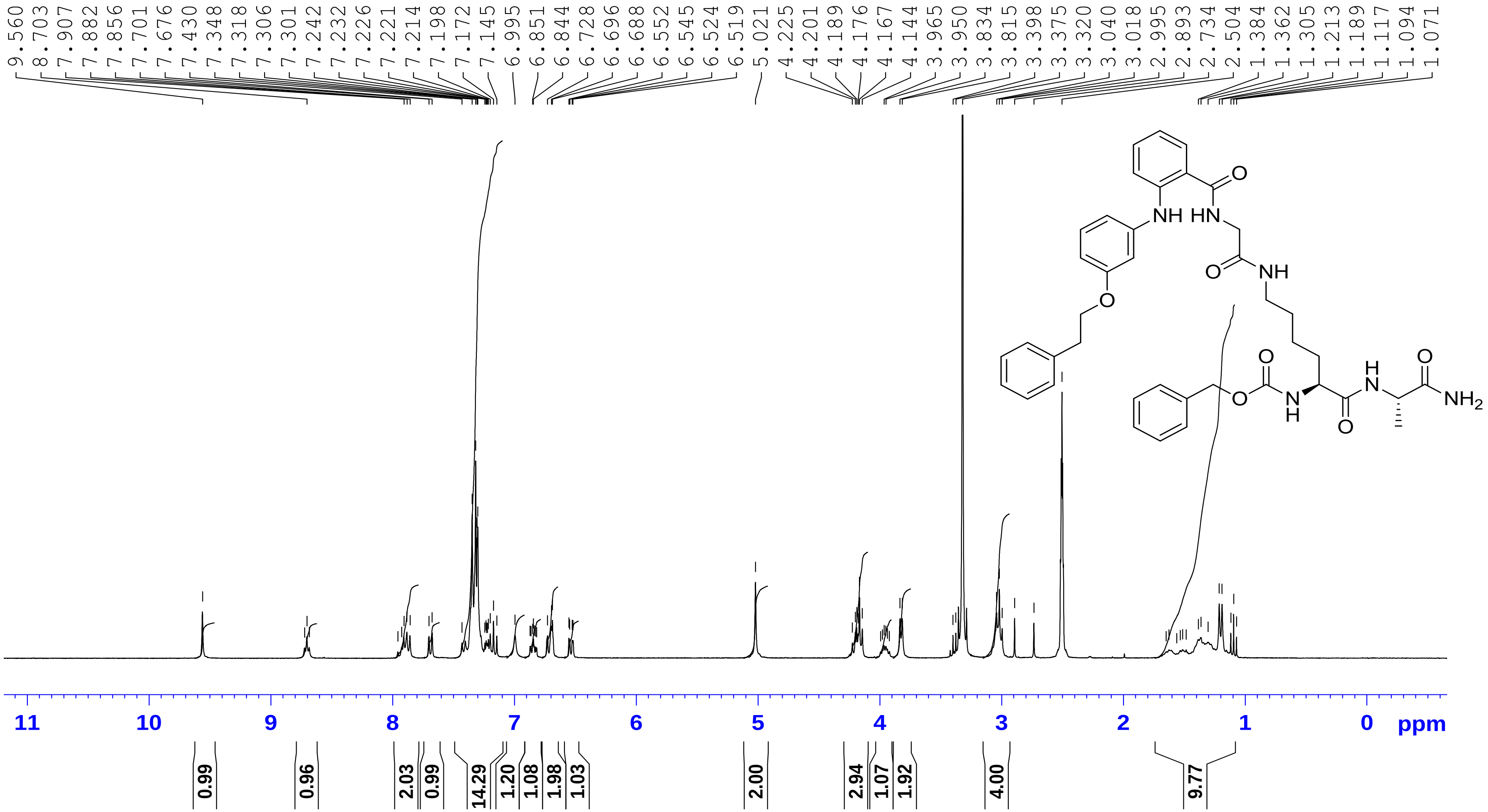

^13C NMR spectrum for **26** in MeOD at 301.3 K

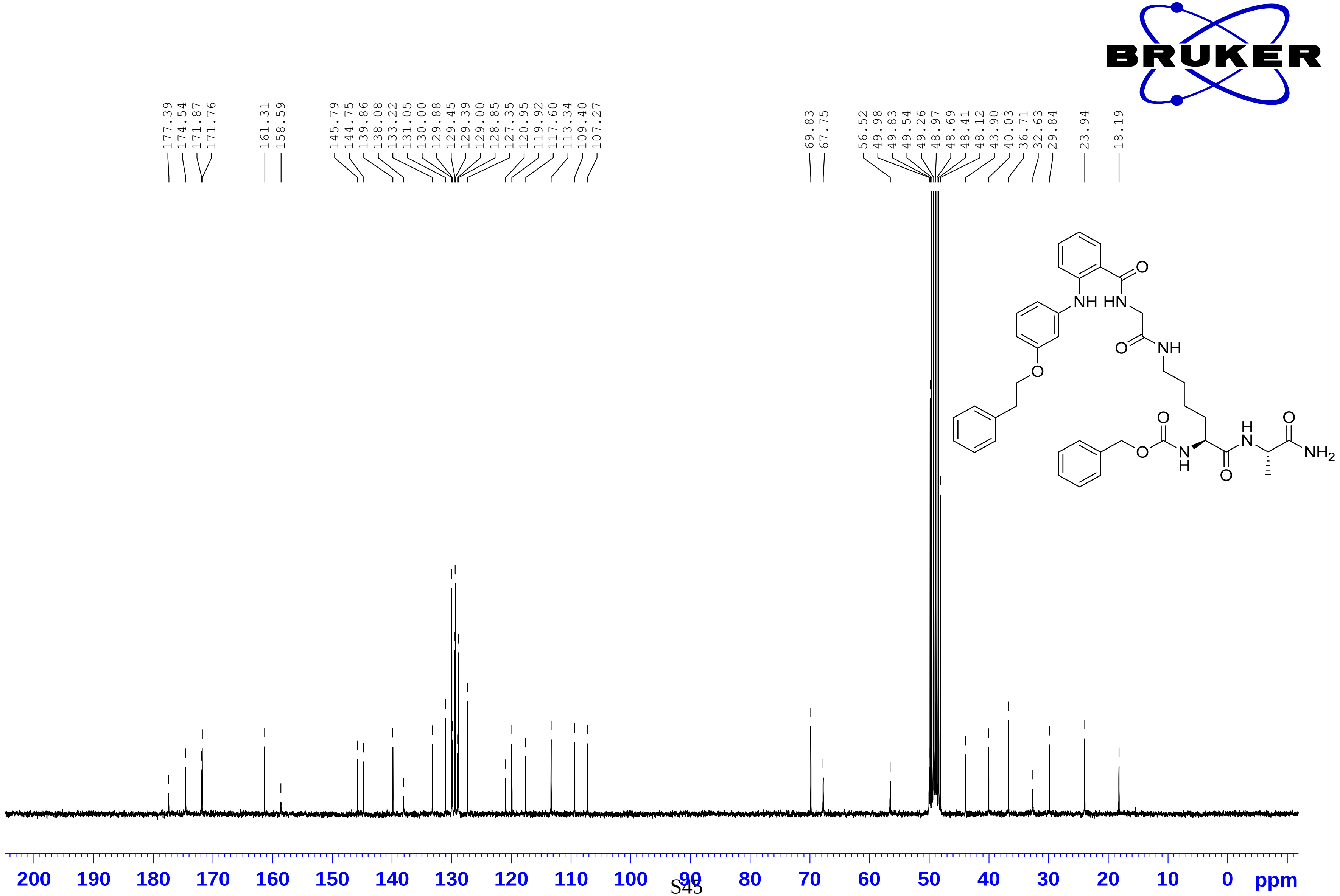

^{1}H NMR spectrum for **27** in DMSO-d_6 at 298.6 K

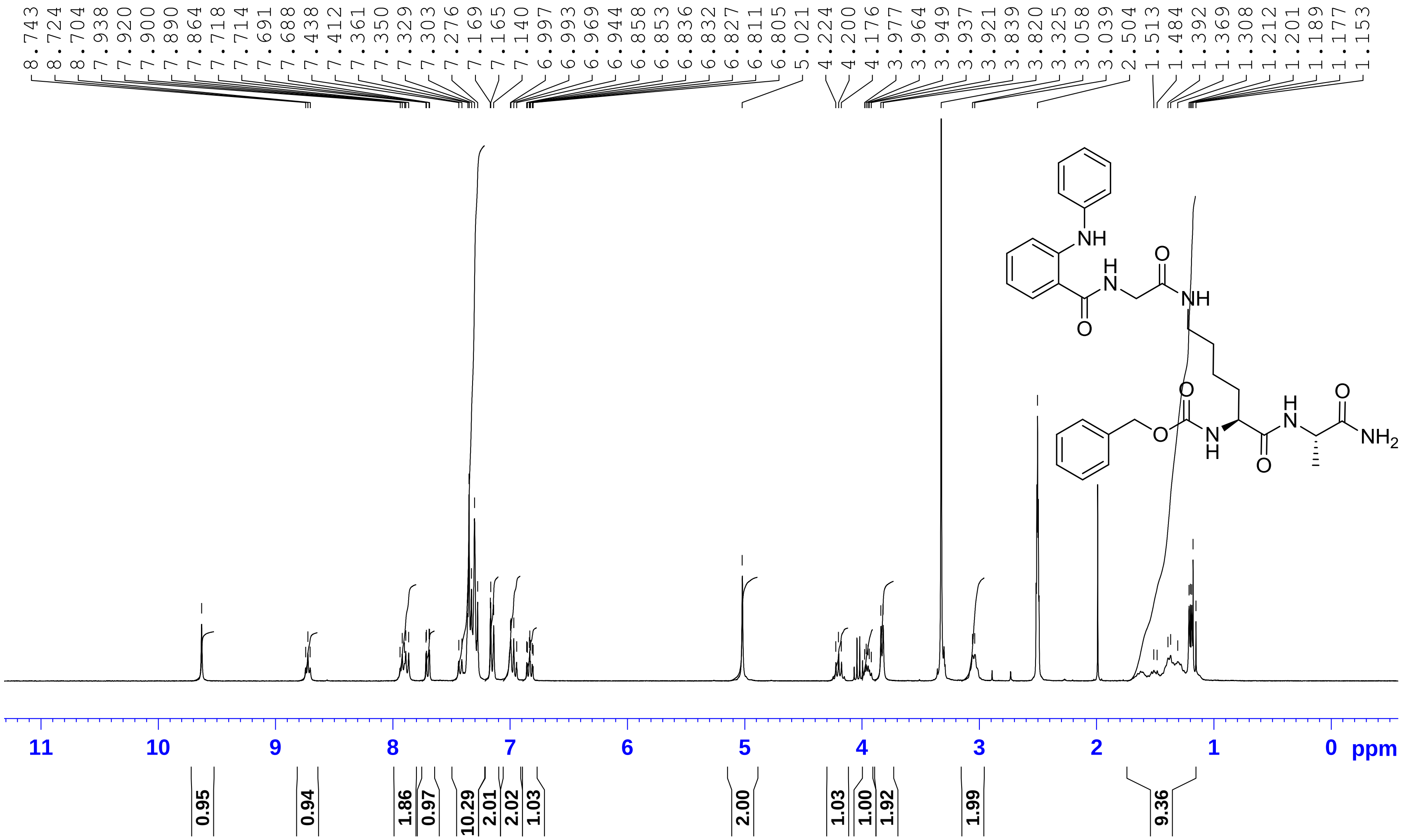

^{13}C NMR spectrum for **27** in DMSO-d_6 at 300.9 K

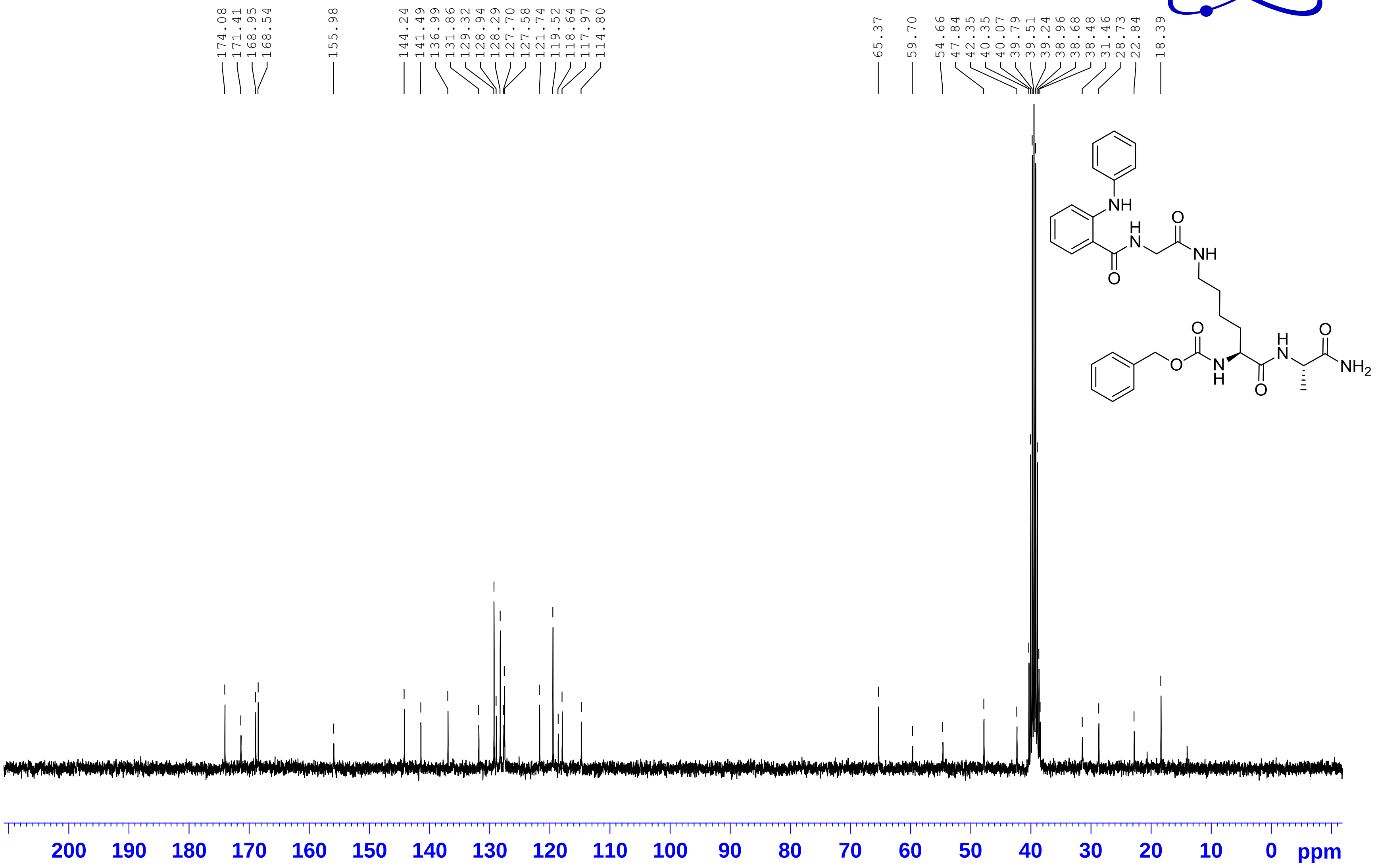

^{1}H NMR spectrum for **28** in DMSO-d_6 at 299.2 K

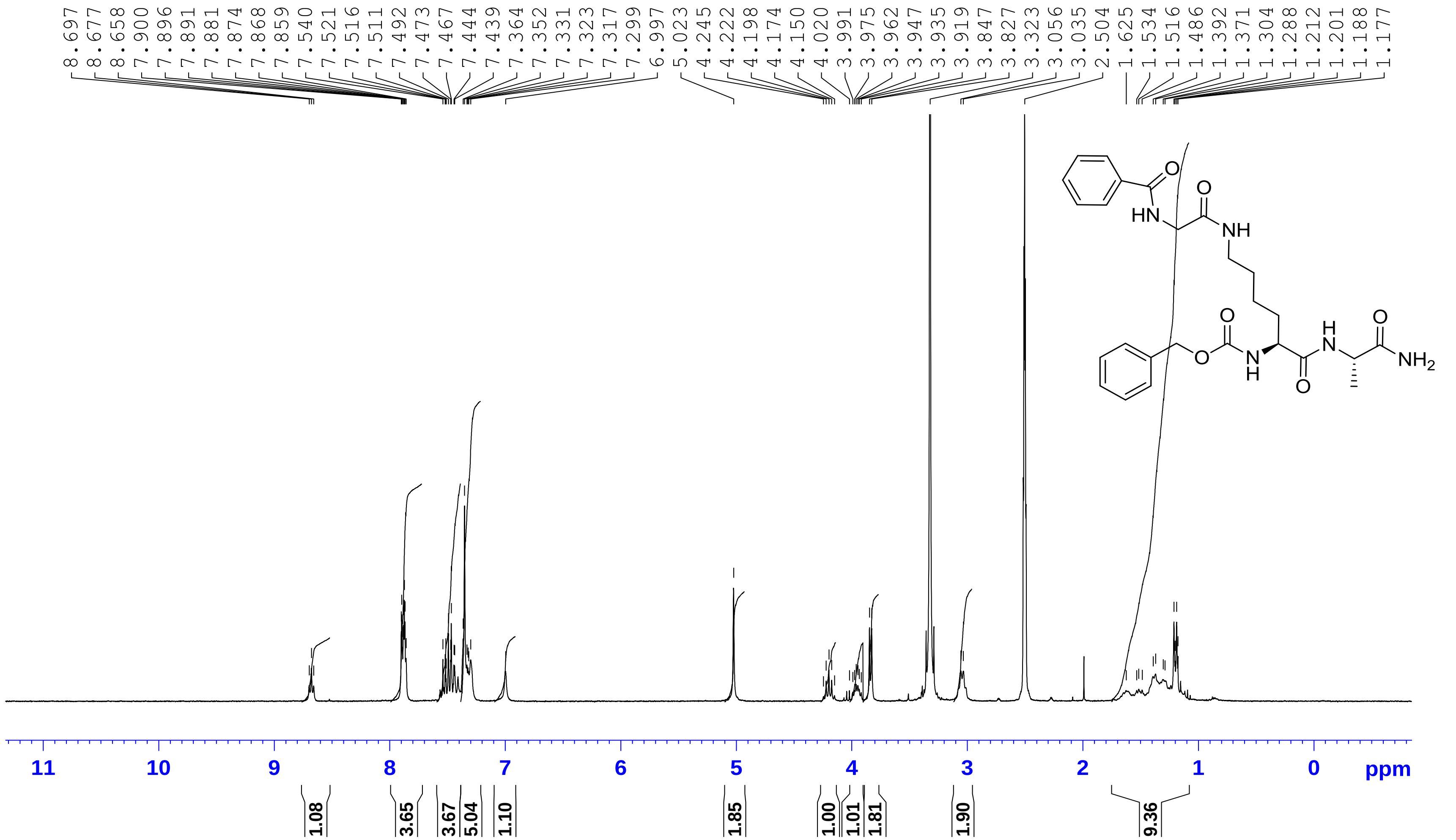

^{13}C NMR spectrum for **28** in DMSO-d_6 at 301.5 K

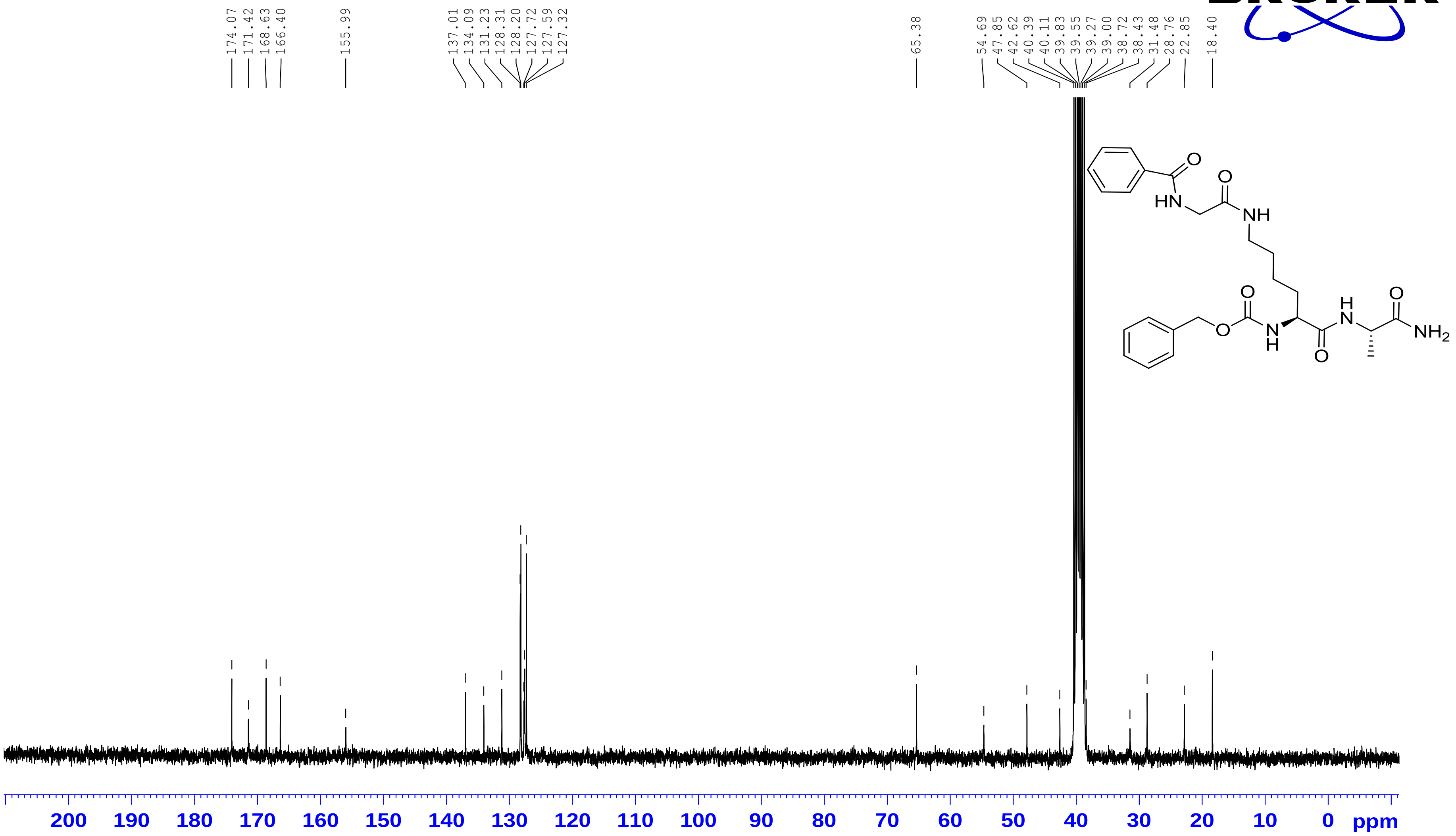

^{1}H NMR spectrum for **36** in DMSO-d_6 at 299.5 K

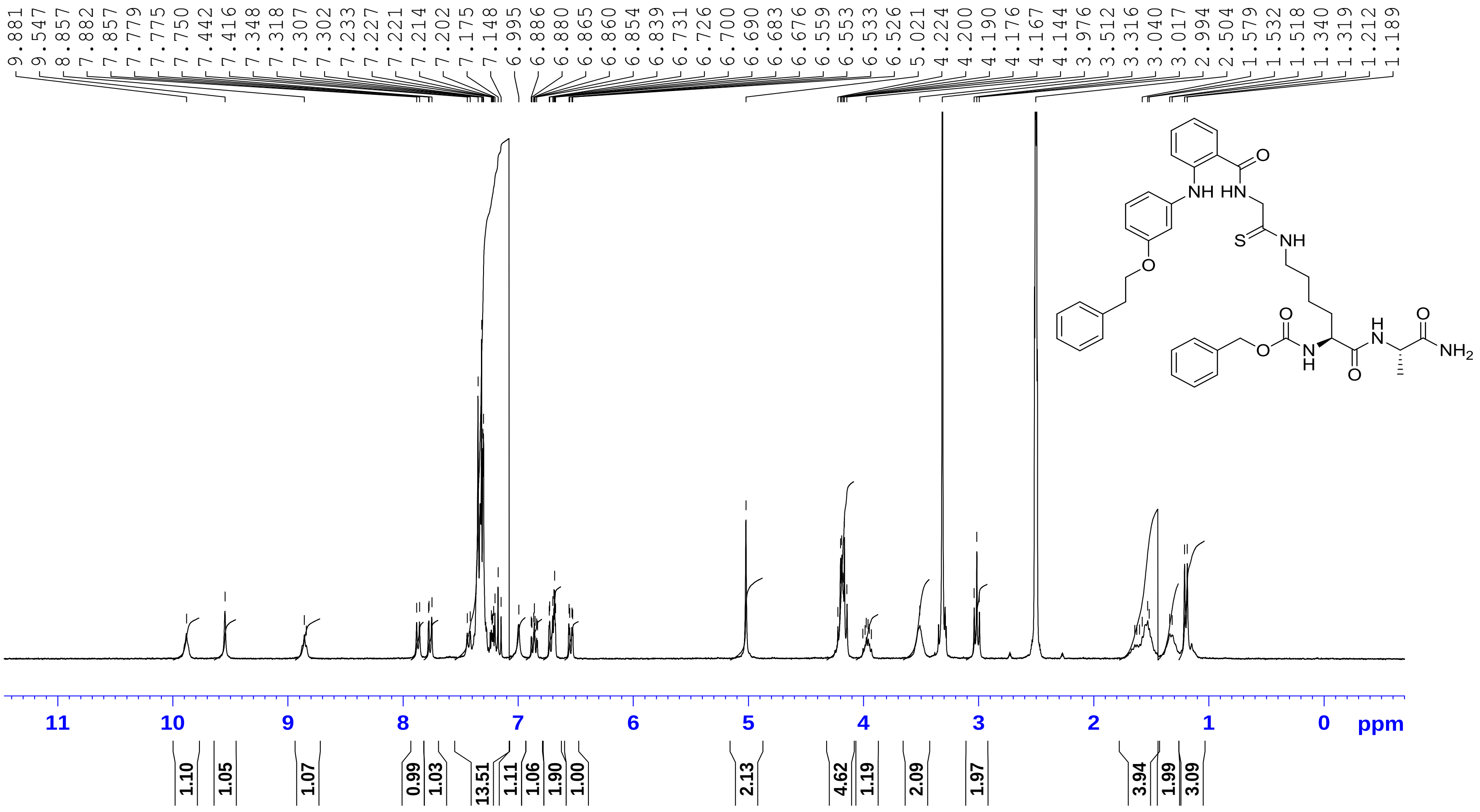

^{13}C NMR spectrum for **36** in DMSO-d_6 at 301.7 K

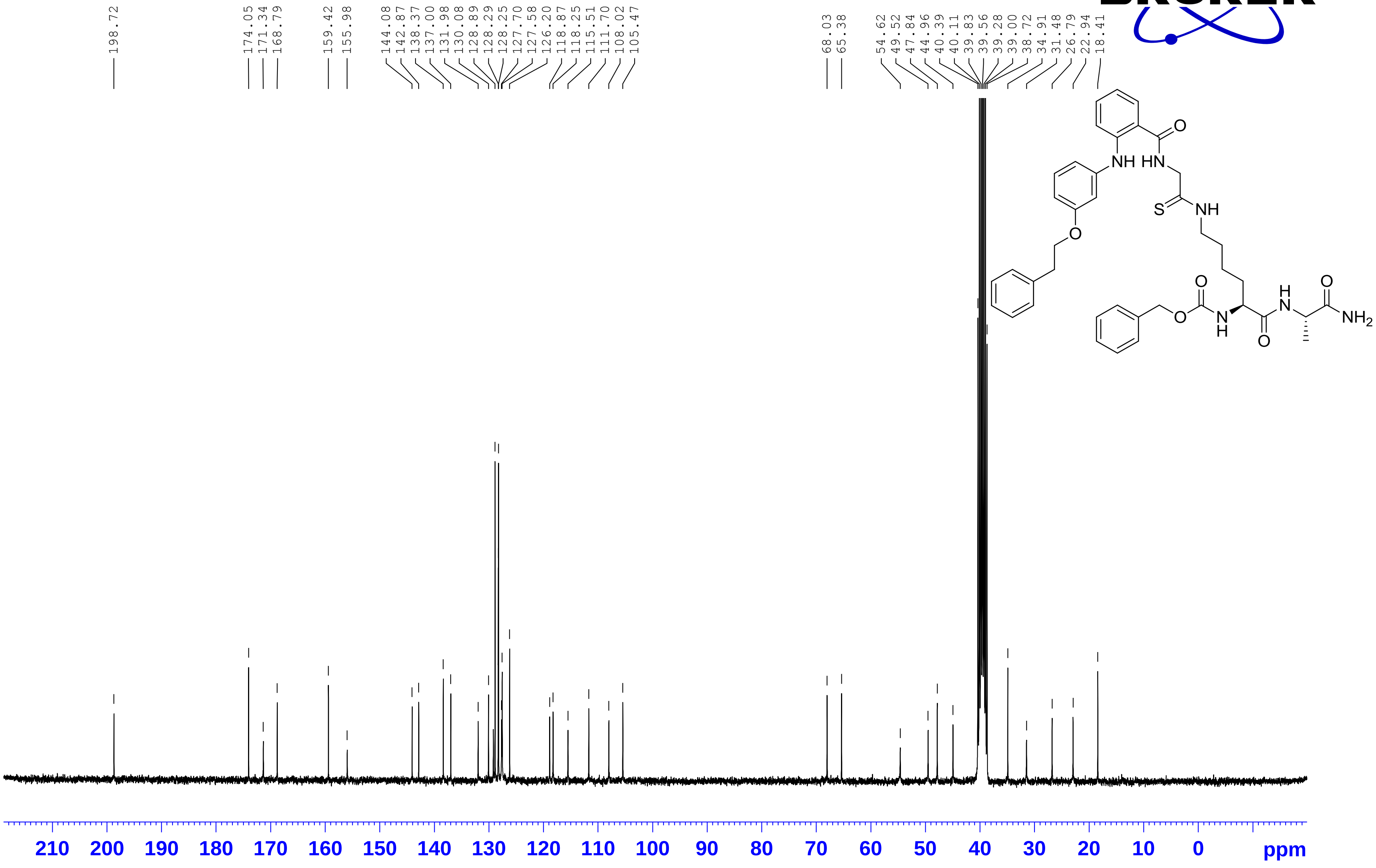

^{1}H NMR spectrum for **41** in DMSO-d_6 at 300.0 K

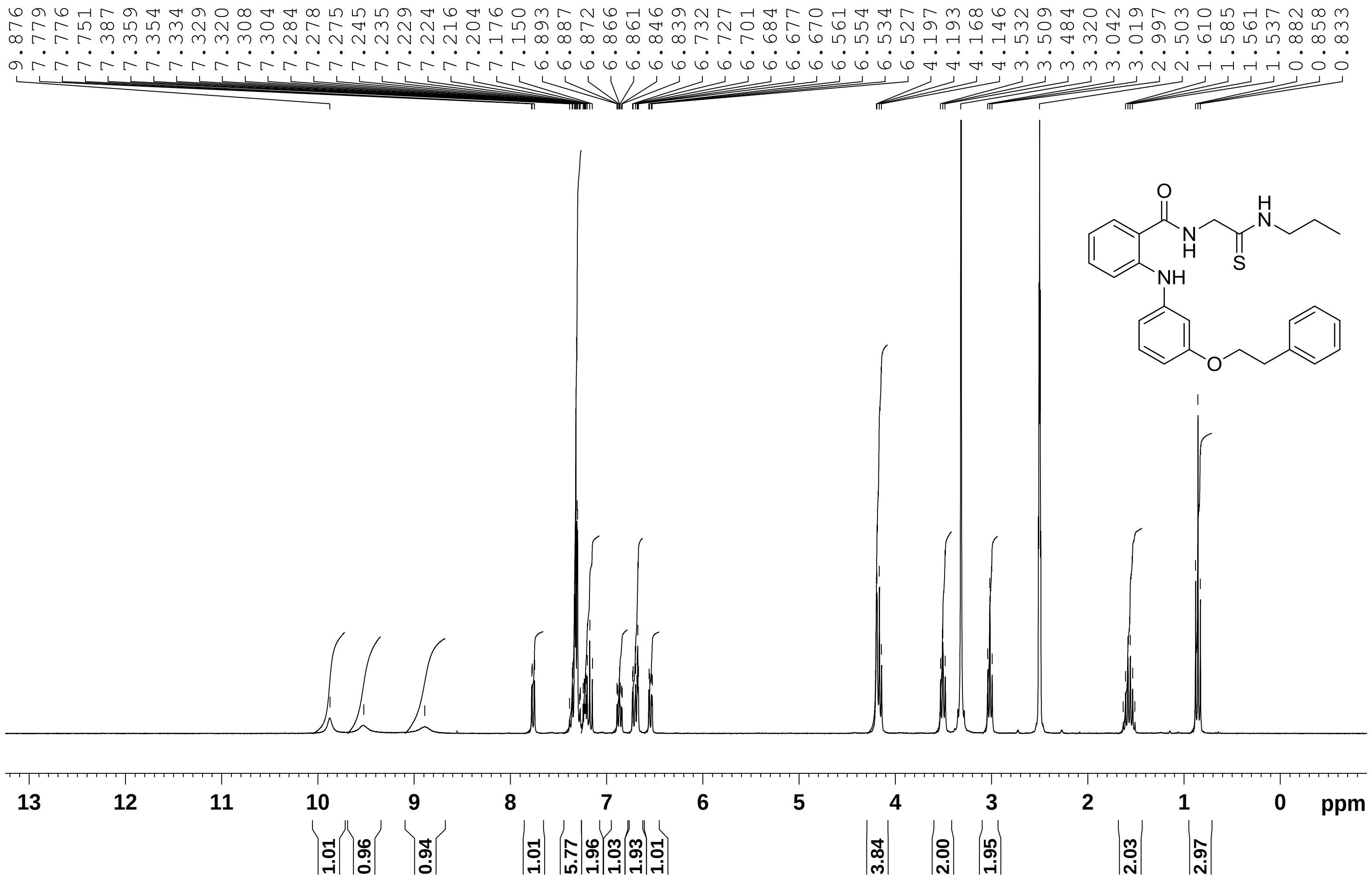

^{13}C-NMR spectra for **41** in DMSO-d_6 at 303.1 K

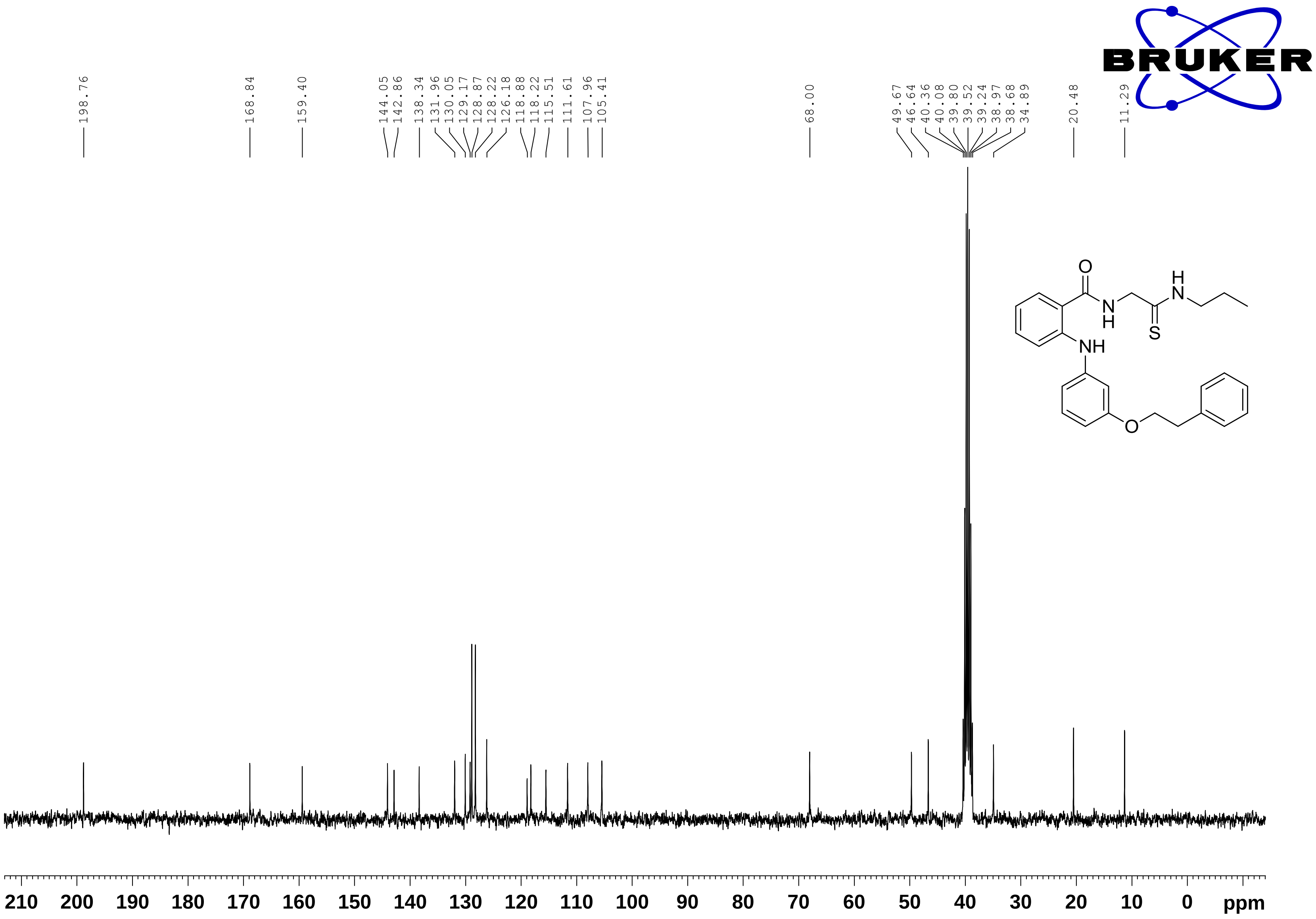

^{1}H NMR spectrum for **42** in DMSO-d_6 at 299.6 K

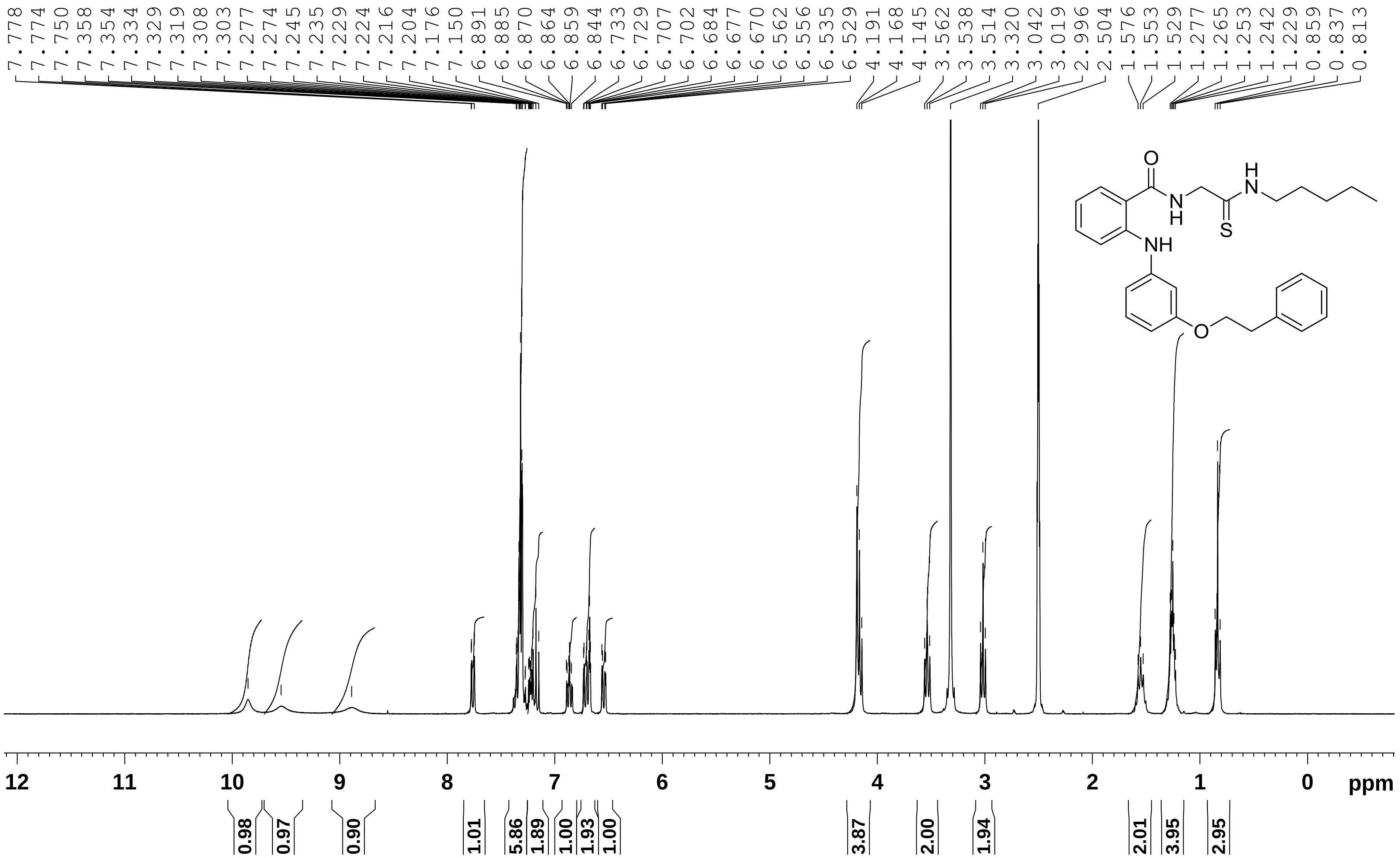

^{13}C-NMR spectra for **42** in DMSO-d_6 at 303.0 K

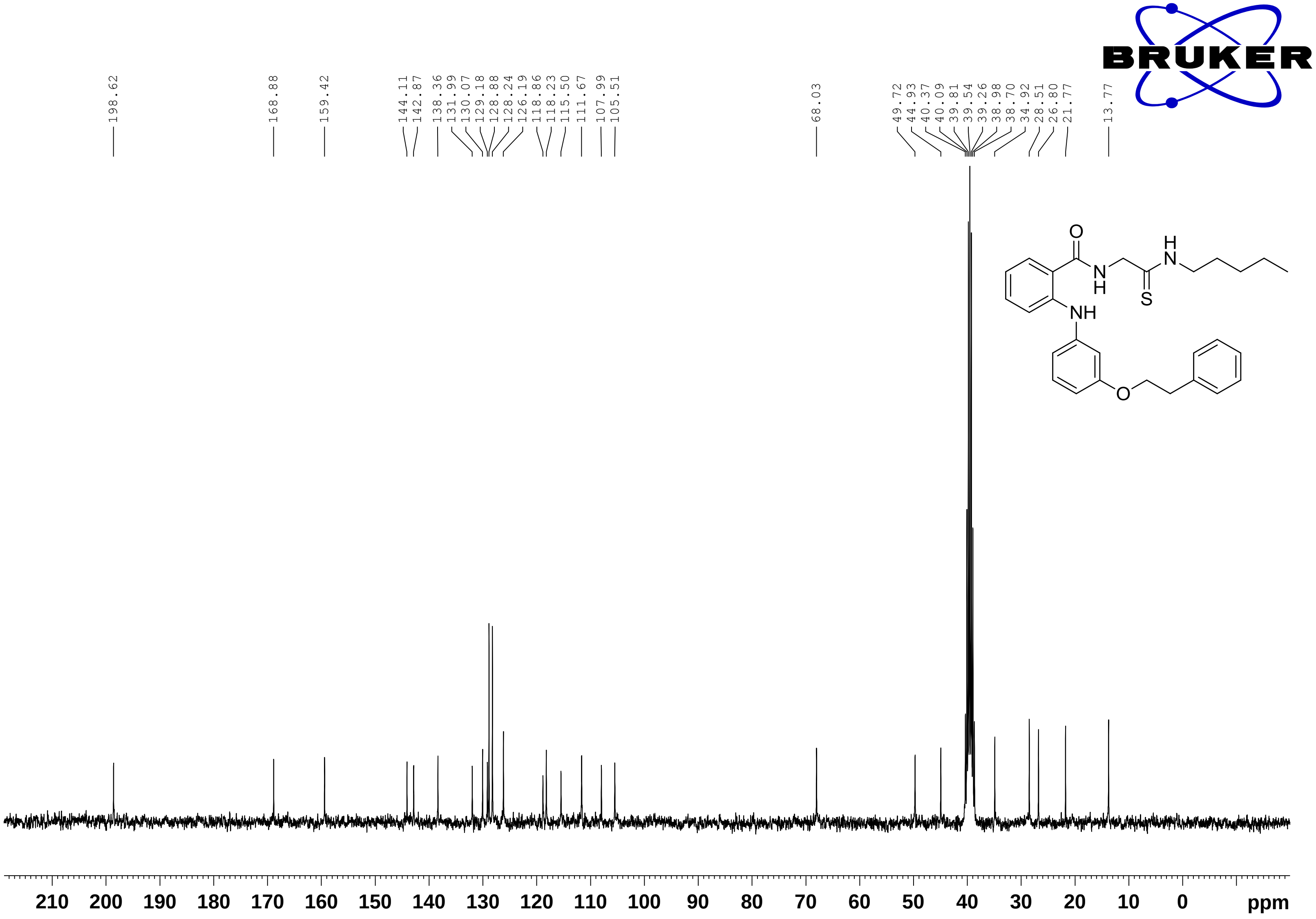

References

(1) K. G. Hoff, J. L. Avalos, K. Sens and C. Wolberger, *Structure*, 2006, **14**, 1231–1240.

(2) W. F. Hawse, K. G. Hoff, D. G. Fatkins, A. Daines, O. V. Zubkova, V. L. Schramm, W. Zheng and C. Wolberger, *Structure*, 2008, **16**, 1368–1377.

(3) B. C. Smith and J. M. Denu, *Biochemistry*, 2007, **46**, 14478–14486.

(4) S. Uchida, K. Hara, A. Kobayashi, K. Otsuki, H. Yamagata, T. Hobara, T. Suzuki, N. Miyata and Y. Watanabe, *Neuron*, 2011, **69**, 359–372.

(5) E. F. Pettersen, T. D. Goddard, C. C. Huang, G. S. Couch, D. M. Greenblatt, E. C. Meng and T. E. Ferrin, *J. Comput. Chem.*, 2004, **25**, 1605–1612.

(6) T. Suzuki, M. N. A. Khan, H. Sawada, E. Imai, Y. Itoh, K. Yamatsuta, N. Tokuda, J. Takeuchi, T. Seko, H. Nakagawa and N. Miyata, *J. Med. Chem.*, 2012, **55**, 5760–5773.

(7) P. Mellini, T. Kokkola, T. Suuronen, H. S. Salo, L. Tolvanen, A. Mai, M. Lahtela-Kakkonen and E. M. Jarho, *J. Med. Chem.*, 2013, **56**, 6681–6695.

(8) T. Rumpf, M. Schiedel, B. Karaman, C. Roessler, B. J. North, A. Lehotzky, J. Oláh, K. I. Ladwein, K. Schmidtkunz, M. Gajer, M. Pannek, C. Steegborn, D. Sinclair, S. Gerhardt, J. Ovádi, M. Schutkowski, W. Sippl, O. Einsle, and M. Jung, *Nat. Commun.*, 2015, **6**, 6263.

(9) A. D. Napper, J. Hixon, T. McDonagh, K. Keavey, J.-F. Pons, J. Barker, W. T. Yau, P. Amouzegh, A. Flegg, E. Hamelin, R. J. Thomas, M. Kates, S. Jones, M. A. Navia, J. O. Saunders, P. S. DiStefano and R. Curtis, *J. Med. Chem.*, 2005, **48**, 8045–8054.
